# Rehabilitation of Motor Function after Stroke: A Multiple Systematic Review Focused on Techniques to Stimulate Upper Extremity Recovery

**DOI:** 10.3389/fnhum.2016.00442

**Published:** 2016-09-13

**Authors:** Samar M. Hatem, Geoffroy Saussez, Margaux della Faille, Vincent Prist, Xue Zhang, Delphine Dispa, Yannick Bleyenheuft

**Affiliations:** ^1^Physical and Rehabilitation Medicine, Brugmann University HospitalBrussels, Belgium; ^2^Systems and Cognitive Neuroscience, Institute of Neuroscience, Université Catholique de LouvainBrussels, Belgium; ^3^Faculty of Medicine and Pharmacy, Faculty of Physical Education and Physiotherapy, Vrije Universiteit BrusselBrussels, Belgium; ^4^Physical and Rehabilitation Medicine, Centre Hospitalier de l'ArdenneLibramont, Belgium; ^5^Movement Control and Neuroplasticity Research Group, Motor Control Laboratory, Department of Kinesiology, Katholieke Universiteit LeuvenLeuven, Belgium; ^6^Physical Medicine and Rehabilitation, Cliniques Universitaires Saint-Luc, Université Catholique de LouvainBrussels, Belgium

**Keywords:** rehabilitation, upper extremity, stroke, review, paresis, systematic review

## Abstract

Stroke is one of the leading causes for disability worldwide. Motor function deficits due to stroke affect the patients' mobility, their limitation in daily life activities, their participation in society and their odds of returning to professional activities. All of these factors contribute to a low overall quality of life. Rehabilitation training is the most effective way to reduce motor impairments in stroke patients. This multiple systematic review focuses both on standard treatment methods and on innovating rehabilitation techniques used to promote upper extremity motor function in stroke patients. A total number of 5712 publications on stroke rehabilitation was systematically reviewed for relevance and quality with regards to upper extremity motor outcome. This procedure yielded 270 publications corresponding to the inclusion criteria of the systematic review. Recent technology-based interventions in stroke rehabilitation including non-invasive brain stimulation, robot-assisted training, and virtual reality immersion are addressed. Finally, a decisional tree based on evidence from the literature and characteristics of stroke patients is proposed. At present, the stroke rehabilitation field faces the challenge to tailor evidence-based treatment strategies to the needs of the individual stroke patient. Interventions can be combined in order to achieve the maximal motor function recovery for each patient. Though the efficacy of some interventions may be under debate, motor skill learning, and some new technological approaches give promising outcome prognosis in stroke motor rehabilitation.

## Introduction

The World Health Organization (WHO) estimates that stroke events in EU countries are likely to increase by 30% between 2000 and 2025 (Truelsen et al., 2006). The most common deficit after stroke is hemiparesis of the contralateral upper limb, with more than 80% of stroke patients experiencing this condition acutely and more than 40% chronically (Cramer et al., 1997). Common manifestations of upper extremity motor impairment include muscle weakness or contracture, changes in muscle tone, joint laxity, and impaired motor control. These impairments induce disabilities in common activities such as reaching, picking up objects, and holding onto objects (for a review on precision grip deficits, see Bleyenheuft and Gordon, 2014).

Motor paresis of the upper extremity may be associated with other neurological manifestations that affect the recovery of motor function and thus require focused therapeutic intervention. Deficits in somatic sensations (body senses such as touch, temperature, pain, and proprioception) after stroke are common with prevalence rates variously reported to be 11–85% (Carey et al., 1993; Yekutiel, 2000; Hunter, 2002). Functionally, the motor problems resulting from sensory deficits after stroke can be summarized as (1) impaired detection of sensory information, (2) disturbed motor tasks performance requiring somatosensory information, and (3) diminished upper extremity rehabilitation outcomes (Hunter, 2002). Sensation is essential for safety even if there is adequate motor recovery (Yekutiel, 2000). Also, up to 50% of patients experience pain of the upper extremity during the first year after stroke, especially shoulder pain and complex regional pain syndrome-type I (CRPS-type I), which may impede adequate early rehabilitation (Jönsson et al., 2006; Kocabas et al., 2007; Sackley et al., 2008; Lundström et al., 2009). Furthermore, joint subluxation and muscle contractures can lead to nociceptive musculoskeletal pain (de Oliveira et al., 2012). Among other complications of stroke the neglect syndrome (Ringman et al., 2004) and spasticity (Sommerfeld et al., 2004; Welmer et al., 2010) affect motor and functional outcomes.

**Figure 1:**
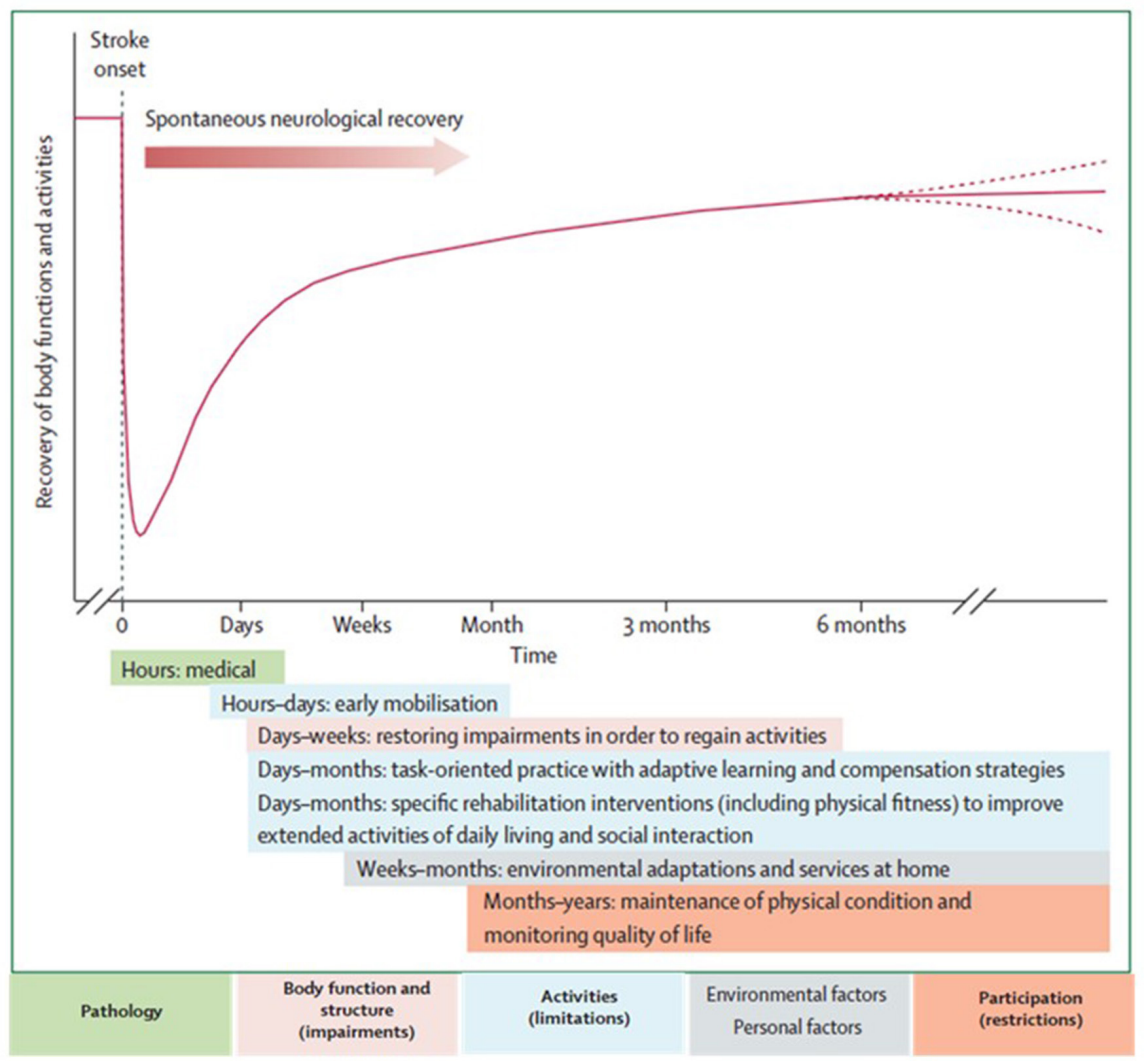
**Hypothetical pattern of recovery after stroke with timing of intervention strategies**. The neurological recovery after stroke displays a nonlinear, logarithmic pattern. The greater part of recovery is reported to take place in the first three months following stroke. Rehabilitation interventions targeting at improving a stroke patients' performance should be implemented according to the phase of neurological recovery. Reprinted from Langhorne et al. (2011), Copyright [2011] by Elsevier. Reprinted with permission.

The neurological recovery after stroke displays a nonlinear, logarithmic pattern (Figure 1; Kwakkel et al., 2006; Langhorne et al., 2011). The greater part of recovery is reported to take place in the first 3 months following stroke (Wade et al., 1983). However, there is evidence that recovery is not limited to this time period; hand and upper extremity recovery has been reported many years after stroke (Carey et al., 1993; Yekutiel and Guttman, 1993). Improvement probably occurs through a complex combination of spontaneous and learning-dependent processes including: restitution, substitution, and compensation (Kwakkel et al., 2004; Langhorne et al., 2011). Until the third month after stroke onset, a variable spontaneous neurological recovery can be considered a confounder of rehabilitation intervention (Kwakkel et al., 2006). In the past, the observation of spontaneous recovery after stroke has misled some authors to believe that recovery of upper extremity function is intrinsic and that little can be done by therapists to influence it (Wade et al., 1983; Heller et al., 1987). Progresses in functional outcome appearing after 3 months seem largely dependent on learning adaptation strategies (Kwakkel et al., 2004). Evidence suggests that neurological repair through brain reorganization supporting true recovery or, alternatively through compensation, may also take place in the subacute and chronic phase after stroke (Krakauer, 2006).

Functional imaging of stroke recovery corroborates this temporal pattern of activation shifts. Shortly after stroke, an initial contralesional shift of activation toward the “unaffected” hemisphere is observed, followed by the activation of learning-related brain structures (including the cerebellum, basal ganglia, and frontal cortices) (Hikosaka et al., 1998; Lehéricy et al., 2005). Finally, two activation patterns are described depending on the degree of recovery (related to the amount of remaining fibers in the impaired corticospinal tract), either a perilesional (refocusing), or a distributed recruitment pattern (Feydy et al., 2002; Ween, 2008). Rehme et al. (2012) confirmed this last assumption and concluded that a good functional outcome relies on the recruitment of the original functional network rather than on contralesional activity. The meta-analysis by Richards et al. (2008) concluded that brain activations increase within the lesioned hemisphere after an upper extremity rehabilitation program. Brain plasticity including reorganization and compensation processes is the base for neurological recovery, as described above, however the exact pathophysiological mechanisms underlying rehabilitation's efficacy remain unclear (Eliassen et al., 2008).

Stroke recovery is heterogeneous in terms of functional outcome. Patients with mild to moderate upper extremity paresis in acute phase have a good prognosis for functional recovery, as 71% of these patients achieve at least some dexterity at 6 months after stroke (Nijland et al., 2010). The prognosis in severely affected patients is poor with about 60% failing to achieve some dexterity at 6 months after stroke (Kwakkel et al., 2003; van Kuijk et al., 2009). Finally, only 5% of patients who initially experienced complete paralysis achieve functional use of their arm. Upper extremity impairments chronically affect the functional independence and satisfaction in 50–70% of all stroke patients. (Bonita and Beaglehole, 1988).

**Figure 2:**
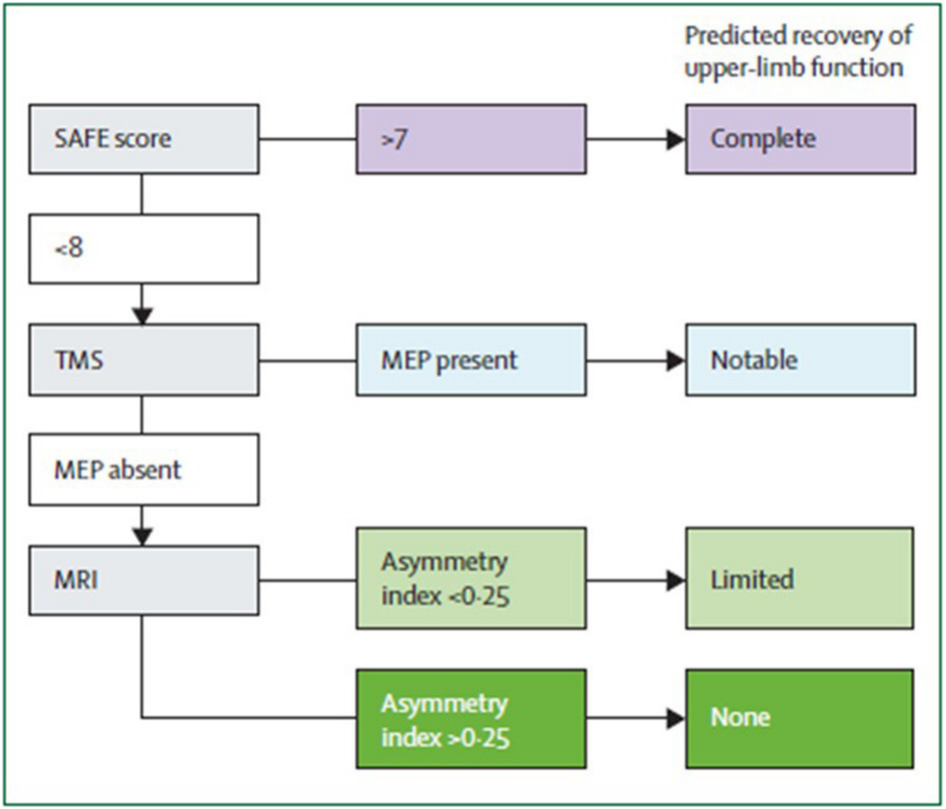
**Suggested sequence of tests to predict the recovery of motor function in patients with subacute stroke (weeks after stroke)**. Although this particular algorithm requires validation, it illustrates a potentially efficient progression from simple to more complex predictive measures. SAFE, sum of muscle force on shoulder abduction and finger extension according to Medical Research Council muscle grades at 72 h after stroke; TMS, transcranial magnetic stimulation; MEP, motor evoked potentials in the affected upper limb; Asymmetry index, asymmetry index of fractional anisotropy in the posterior limbs of the internal capsules measured with diffusion-weighted MRI. From Stinear et al. (2014).

Algorithms have been developed to predict motor function recovery after stroke (Stinear et al., 2007). Predictor variables include age, sex, lesion site, initial motor impairment, motor-evoked potentials, and somatosensory-evoked potentials. Initial measures of upper extremity impairment and function were found to be the most significant predictors of upper extremity recovery (Coupar et al., 2012). Findings so far suggest that the first assessments should be quick and simple, such as bedside tests of motor impairment, with progression to more complex tests if uncertainty remains (Figure 2). Later tests can include neurophysiological assessments and neuroimagery of the motor system integrity.

Interdisciplinary complex rehabilitation interventions represent the mainstay of post-stroke care (Langhorne and Legg, 2003; Langhorne et al., 2011). Stroke rehabilitation aims at providing all possible means to recover lost function and to increase the autonomy of stroke patients taking into account the remaining impairments and disabilities. Carr and Shepherd (2011) suggested that poor upper extremity recovery may be due to the direct impact of the stroke itself as well as to insufficient, inadequate or inappropriate therapeutic interventions. Little information is available, however, to describe what best represents “optimum treatment” (Ballinger et al., 1999). From a theoretical point of view, a stroke rehabilitation program for upper extremity motor impairment should include global motor rehabilitation, electrical brain stimulation, hemispheric subspecialization in motor activities, and multisensory interaction (Johansson, 2011). A recent Cochrane review focussing on the recovery of function and mobility in stroke patients reported the potential benefit of rehabilitation therapy on motor impairments and disabilities, compared with no treatment, in function of the time since stroke (Pollock et al., 2014). While these type of systematic reviews and meta-analyses are very powerful, they only take into account rehabilitation techniques that already have been reported in other systematic reviews and may thus ignore rehabilitation approaches that pertain to the routine clinical setting. Furthermore, in most systematic reviews only randomized controlled trials are reported.

The purpose of the present manuscript was to undertake a systematic review for each of the neurorehabilitation techniques that may be useful in promoting upper extremity motor recovery. The search terms and inclusion criteria of reported trials have been chosen as large as possible in order to detect pertinent information on rehabilitation methods that are currently used in clinical practice, but are uncommonly discussed in systematic reviews (examples: music therapy, motor skill learning, isokinetic muscle strengthening, paired associative stimulation, theta burst stimulation). In some cases, routine clinical treatments that have not been investigated in a randomized controlled way, are still included in the present systematic review if the trial demonstrated sufficient quality evidence. The scientific evidence of each stroke rehabilitation intervention is discussed and presented with a practical recommendation for clinicians working in the field of neurorehabilitation. A decisional tree according to the patient's characteristics is proposed based on scientific evidence available for the different interventions.

## Methods

**Figure 3:**
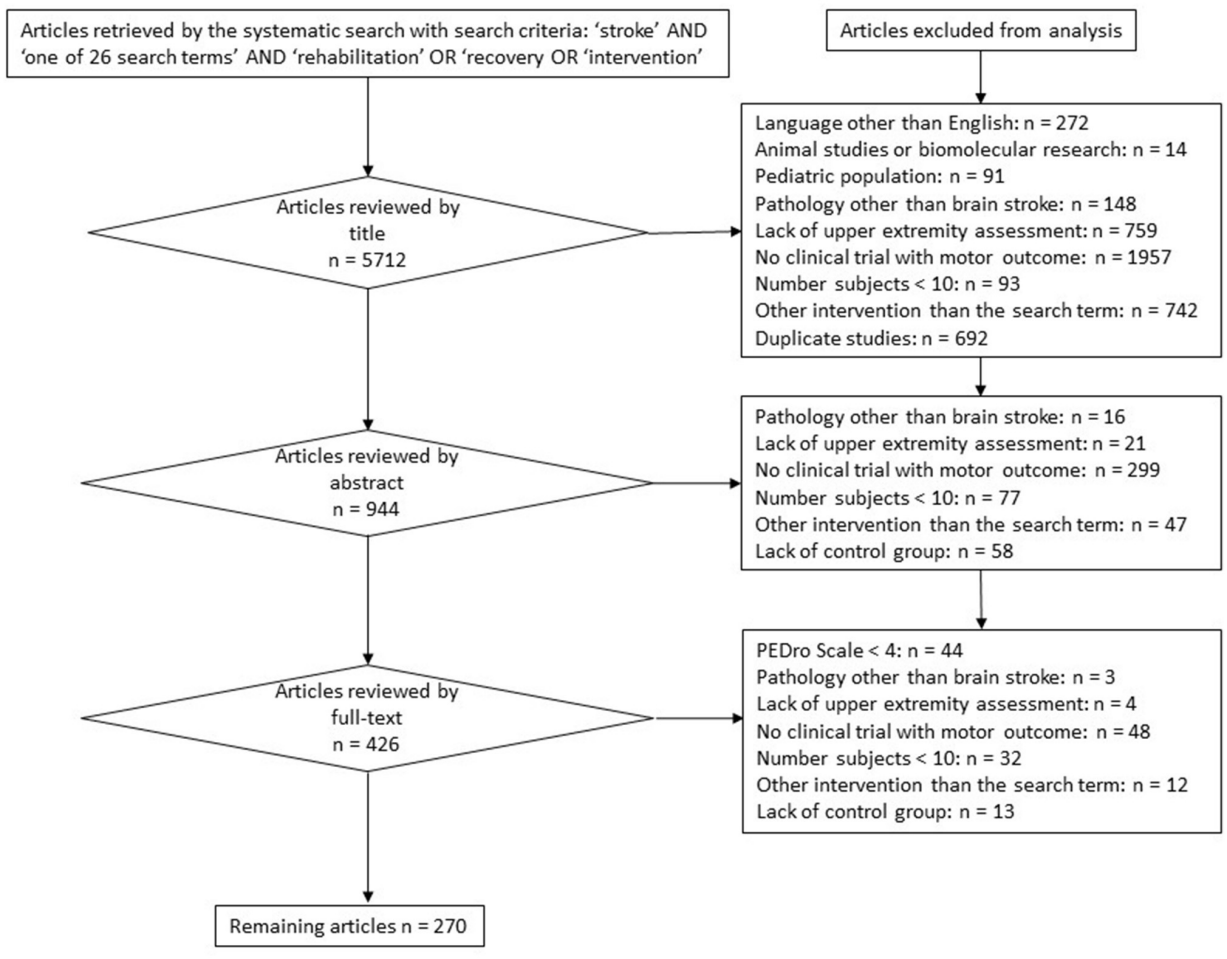
**PRISMA diagram reporting the flowchart, exclusion criteria, and stages of the systematic review**.

This manuscript is based on multiple systematic reviews. Twenty-six different rehabilitation treatment modalities were included and searched for with the following search terms: Bobath, Picard, Perfetti, muscle strengthening, isokinetic muscle strengthening, stretching, bilateral training, forced-use, motor skill learning, constraint induced movement, mirror therapy, motor imagery, motor imitation, movement observation, transcutaneous electrical nerve stimulation, neuromuscular electrical stimulation, positional feedback, repetitive transcranial magnetic stimulation, transcranial direct current stimulation, deep brain stimulation, paired associative stimulation, antidepressants, botulinum toxin, robot-assisted, virtual reality, music. The 26 search terms were chosen by a panel of experts in neurorehabilitation (SH, YB, VP, DD). The systematic database search and article selection was performed by two independent investigators (SH and YB). Each of the 26 search terms was combined with the keyword “stroke” and with each of the following three keywords: “rehabilitation” or “intervention” or “recovery.” The search was performed by a hand search and by using the internet databases: medline and pubmed, retrieving articles from 1971 until May 2015, and yielded a total number of 5712 publications. Exclusion criteria at each stage of the review process are reported in a general prisma diagram. The outcome of this multiple review process includes randomized-controlled trials (RCTs), controlled trials, systematic reviews, and meta-analyses with a PEDro-score higher than or equal to 4 (Maher et al., 2003). The PEDro score was assessed by two independent investigators (GS and MdF) and scored on a scale from 0 to 10. PEDro scores lower than 4/10 were regarded as methodologically low-quality trials (and excluded from the systematic review), scores of 4–7/10 as methodologically moderate-quality trials and scores higher than 7/10 as methodologically high-quality trials. Furthermore, the Oxford level of evidence was assessed for each remaining publication. From a rehabilitation point of view, the phase of stroke was defined as acute within the first month, as subacute between 1 month and 6 months, and as chronic if longer than 6 months after stroke occurrence (Teasell et al., 2014; Hebert et al., 2016). After having excluded trials not corresponding to the inclusion criteria as described in the PRISMA diagram (Figure 3), a qualitative recommendation on the implementation of each rehabilitation intervention is issued, based on the UE motor outcome and on the amount of evidence of the trials remaining in the systematic review. A treatment modality is not recommended as a rehabilitation intervention or as an adjuvant treatment because of a *lack of scientific evidence*, if a total number of less than 500 subjects has been included in trials selected in the systematic review. A treatment modality is not recommended as a rehabilitation intervention because of a *lack of effectiveness*, if (1) it has shown non-superior (similar or inferior) efficacy compared to another rehabilitation intervention and (2) a sufficient amount of evidence is available, defined as a total number of at least 500 subjects included in trials selected in the systematic review. A treatment modality is recommended *as a rehabilitation intervention*, if it has shown superior efficacy compared to another rehabilitation intervention. A treatment modality is recommended *as an adjuvant intervention* for rehabilitation treatment, if it has shown superior efficacy *in combination with another rehabilitation intervention* compared to the other rehabilitation intervention alone. These recommandations as a rehabilitation intervention or as an adjuvant intervention only are issued if a sufficient amount of evidence is available, defined as a total number of at least 500 subjects included in trials selected in the systematic review.

The twenty-six different rehabilitation treatment modalities have been classified in six different chapters in this manuscript: (1) Neurofacilitatory approaches/multiple exercising approaches; (2) Isolated concepts; (3) Motor learning; (4) Interventions based on the hypothesis of mirror neurons and motor imagery; (5) Adjuvant therapies; and (6) Technology-supported training. For each chapter, results of the systematic review are highlighted and in the general discussion, a decisional tree is proposed for therapeutic intervention based on current scientific evidence.

## Results

The systematic search yielded 5712 publications. After the systematic selection of articles following the general flowchart described in Figure 3, the remaining 270 publications (total number of subjects = 41,069) have been included in the systematic review. Their contents, Oxford levels of evidence and PEDro quality scores were assessed and reported in a summary table per rehabilitation technique (Supplementary Tables 1–19). Within each summary table, publications have been ordered by the following criteria: (1) type of publication (first systematic reviews/meta-analyses, then RCT and other types of trials), (2) subtype of rehabilitation technique within the “search term” (example: first rehabilitation technique by itself, then rehabilitation technique in combination with another rehabilitation intervention), (3) in descending chronological order of publication year. In this multiple systematic review, a short description of each rehabilitation technique is followed by a general survey of available evidence and by a clinical recommendation concerning its implementation in stroke rehabilitation with a view to improving the UE motor outcome of stroke patients.

## Neurofacilitatory concepts/multiple exercising approaches

Exercise therapy is a key element of stroke rehabilitation. Exercises performed after stroke may differ with regards to their objectives (goal-directed, task-oriented, repetitive task training) or their technical characteristics (duration, training load, and type of feedback). These specific elements of exercise therapy are described in a Supplementary Material file.

### Bobath concept (supplementary table 1)

The Bobath concept was developed by Berta and Karl Bobath. The Bobath treatment aims at normalizing tone and facilitate volitional movement through handling of specific points (trunk, pelvis, shoulders, hands, and feet) in order to guide patients through the initiation and completion of intended tasks (Bobath, 1990). Both the patient and the therapist need to participate actively during the treatment. The International Bobath Instructors Training Association (IBITA) has introduced the concept of problem solving strategies to the Bobath approach and highlighted its will to have an impact on activity and participation (Lennon and Ashburn, 2000).

The systematic review yielded 8 RCTs (*n* = 475) and 2 systematic reviews/meta-analyses (at least *n* = 209) (Supplementary Table 1). There is moderate- to high-quality evidence indicating that Bobath therapy is similar or inferior to other rehabilitation approaches (meaningful task-specific training, constraint-induced movement therapy, ARM-basis training, motor relearning program, movement science-based physiotherapy) for treating upper limb motor impairment and disabilities in acute, subacute and chronic stroke patients. One moderate-quality RCT indicates that Bobath therapy may be useful in patients with spasticity (Wang et al., 2005).

Based on a sufficient amount of evidence (*n* > 500) indicating the non-superiority of Bobath therapy, at present, there are insufficient arguments for integrating Bobath therapy into stroke rehabilitation with a view to improving UE motor impairments or disabilities.

### Perfetti rehabilitation method

Perfetti's method is a cognitive sensory-motor training focusing on the perception of joint position. The systematic review with the proposed search terms did not yield any publications matching the inclusion criteria. On hand search, one RCT was found including acute stroke patients (*n* = 40) and showing no difference in motor outcomes between Perfetti's method and standard occupational therapy with regards to hand and arm impairments (Chanubol et al., 2012).

Based on a lack of evidence (*n* < 500), at present, there are insufficient arguments for integrating Perfetti's method into stroke rehabilitation with a view to improving UE motor impairments or disabilities.

### Picard rehabilitation method

The systematic review with the search term “Picard” did not yield any publications matching the inclusion criteria.

Based on a lack of evidence (*n* < 500), at present, there are insufficient arguments for integrating Picard rehabilitation into stroke rehabilitation with a view to improving UE motor impairments or disabilities.

## Isolated concepts

In contrast with multiple exercising concepts presented in the previous chapter, specific isolated rehabilitation techniques (sometimes used as part of multiple exercising concepts) have been fully described and their effects tested. These isolated rehabilitation concepts will now be discussed.

### Muscle strengthening exercises (supplementary table 2)

Muscle strengthening techniques are progressive active exercises against resistance for the paretic arm. These exercises can be performed against a manual resistance (exerted by the therapist) or using weight-bearing apparatus. Muscle strengthening and endurance training in stroke rehabilitation for long have been decried for their supposed induction of spasticity, but now have been recovered as an essential part of the rehabilitation programs offered to brain-lesioned patients (Patten et al., 2004; Daly et al., 2005).

The systematic review (Supplementary Table 2) yielded 3 systematic reviews (at least *n* = 517) comparing strengthening exercises of the upper limb either to strengthening exercises of the lower limb or to standard therapy. The total number of subjects in these reviews could not be retrieved. There is moderate-quality evidence indicating that strengthening exercises are useful for increasing UE impairments (strength), without or with poor improvement at the level of disabilities, in acute, subacute, and chronic stroke patients.

Based on a sufficient amount of evidence (*n* > 500) indicating the superiority of muscle strengthening, muscle strengthening exercises appear to be valuable and could be integrated into stroke rehabilitation strategies with a view to improving UE motor impairments.

### Isokinetic muscle strengthening (supplementary table 3)

Isokinetic muscle strengthening uses computer-driven isokinetic dynamometers which allow training for muscle strength or assessing muscle force.

The systematic review (Supplementary Table 3) retrieved one review article: (Hammami et al., 2012). This review article included two studies on isokinetic training of the UE after stroke. None of the studies were CTs (one open study, one clinical case) and the number of included subjects was not retrieved. It appears that there is no consensus on the type of contraction mode (concentric vs. eccentric) that should be used for training the UE, nor on the dosage regimen of training nor on the muscles that should be trained. At present, no studies have examined the usefulness of isokinetic strengthening of wrist and finger muscles. The current evidence is not sufficient to claim the superiority of isokinetic muscle strengthening exercises over conventional strengthening exercises. Randomized controlled studies of isokinetic muscle strengthening of the UE after stroke are needed.

Based on a lack of evidence (*n* < 500), at present, there are insufficient arguments for integrating isokinetic muscle strengthening into stroke rehabilitation with a view to improving UE motor impairments or disabilities.

### Stretching (supplementary table 4)

For years, the prevention of range of joint motion loss, notably due to spasticity, has led to the application of arm stretch positioning during regular physiotherapy (Ada and Canning, 1990). Stretching may be executed by hands-on physical therapy or by application of devices (cast, splint, and taping).

The systematic review (Supplementary Table 4) yielded three RCTs (*n* = 107) and 2 systematic reviews (*n* = 1384). There is moderate- to high-quality evidence indicating that stretching is similar to control rehabilitation approaches for treating upper limb impairments (strength, ROM) and disabilities in subacute and chronic stroke. No significant effects were found of stretching in comparison with control interventions with regards to passive range of motion, pain or activities of daily living. While manual passive stretching has not been proven effective, physical contentions have shown interest for the treatment of spasticity. Gains of range of motion, with an impact on spasticity and motor impairments have been observed through the use of long-term contention, (i.e., taping), notably after injection of botulinum toxin A (Santamato et al., 2014; see Supplementary Table 16 Botulinum toxine). Randomized controlled studies of device-assisted stretching of the UE after stroke are needed.

Based on a sufficient amount of evidence (*n* > 500) indicating the non-superiority of stretching therapy, at present, there are insufficient arguments for integrating stretching into stroke rehabilitation with a view to improving UE motor impairments or disabilities.

### Bilateral training (supplementary table 5)

Bilateral upper extremity training after stroke is based on the premise that movement of the non-paretic upper limb supports movement of the paretic upper limb when performed simultaneously. This type of therapy has a relatively short history arising partly serendipitously (Mudie and Matyas, 1996, 2000) and partly from insights gleaned from the motor control literature. Bilateral training consists of repetitive movements of the upper extremities in a symmetric or asymmetric design. Coupling (or interaction) effects between the two upper extremities have been investigated extensively in rhythmic interlimb-coordination studies involving healthy subjects (Cohen, 1971; Kelso et al., 1979; Swinnen et al., 2002; Ridderikhoff et al., 2005). It is well established that humans show a basic tendency toward in-phase (i.e., symmetrical movements) or anti-phase (i.e., alternating movements) coordination, with a prevalent 1:1 frequency locking mode for upper extremity bilateral movements (Swinnen et al., 2002). Intact transcallosal and interhemispheric connections are a condition sine qua non to exploit these coupling mechanisms in bilateral arm training. According to current evidence, mechanisms underlying improvement from bilateral training include the recruitment of ipsilateral corticospinal pathways, increased control from the contralesional hemisphere and normalization of inhibitory mechanisms. Ipsilateral corticospinal pathways have been demonstrated to exist as parts of the CST that do not cross at the pyramidal decussation. The estimated percentage of uncrossed pathways is 10–20% (Chollet et al., 1991), and some researchers suggest that their activation could be facilitated with bilateral training (Mudie and Matyas, 2000).

Bilateral training can be performed with or without the assistance of an external device. Apart from using bilateral training as a rehabilitation technique *per se*, it can also be used as a priming treatment before other interventions (Stinear et al., 2014). Without the assistance of an external device, the therapist instructs patients to move the impaired upper extremity simultaneously (Kumar et al., 1990) or alternatingly (Whitall et al., 2000; Luft et al., 2004) with the healthy one. Robotic devices used for bilateral arm training, are mostly interactive one-degree of freedom systems such as the Bilateral arm training with rhythmic auditory cueing (BATRAC) (Whitall et al., 2000), the Bi-manu-track (Hesse et al., 2003, 2005, 2007), and the Active and passive bilateral training (APBT) with the Rocker device. These robotic devices are typically used for bilateral arm training and their mechanisms of action are based on the same premises as non-device assisted bilateral arm training. For this reason, the publications concerning robot-based bilateral training are described in this systematic review summary of bilateral training (Supplementary Table 5) and not in the systematic review section of robotic devices (Supplementary Table 17). In contrast with therapies promoting bimanual coordination and motor learning [see Section Motor Skill Learning—Constraint-Induced Movement Therapy (Supplementary Table 7) on motor skill learning], bilateral training exercises typically are not goal-oriented and not associated with motor skill learning techniques.

The systematic review (Supplementary Table 5) yielded 23 RCTs (*n* = 1104), 1 controlled trial (*n* = 23) and 7 meta-analyses/systematic reviews (at least *n* = 2240). There is moderate- to high-quality evidence that bilateral arm training (non-device assisted or device-assisted) is similar or inferior to unilateral arm training or to standard rehabilitation treatment. Any gains are specific for the task that is being trained (motor impairment) and do not extrapolate to upper extremity disabilities in daily life. The efficacy of bilateral arm training does not appear to be specific for a post-stroke phase. One study (*n* = 24) indicates that bilateral training may improve spasticity in chronic stroke (Stoykov et al., 2009), and two other studies failed to report any effect on the modified Ashworth scale for spasticity.

At present, it appears that bilateral training, though based on neurophysiological evidence, does not hold up its promise for clinical purpose. Based on a sufficient amount of evidence (*n* > 500) indicating the non-superiority of bilateral training, at present, there are insufficient arguments for integrating bilateral training into stroke rehabilitation with a view to improving UE motor impairments or disabilities.

### Forced-use (supplementary table 6)

Forced-use consists in favoring the unimanual use of the paretic upper extremity by restraining the non-paretic upper extremity (by a cast, sling, etc.). However, in contrast with constraint-induced movement therapy (CIMT, see section Motor skill learning), forced-use is not associated with specific motor skill learning techniques. Restraint of the non-paretic upper extremity is performed without specific training, or using usual care.

The systematic review (Supplementary Table 6) yielded 3 RCTs (*n* = 96). There is moderate-quality evidence that forced-use is similar to standard rehabilitation therapy or to bimanual training with regards to UE motor impairments or disabilities. Randomized controlled studies of forced-use of the UE after stroke are needed.

Based on a lack of evidence (*n* < 500), at present, there are insufficient arguments for integrating forced-use into stroke rehabilitation with a view to improving UE motor impairments or disabilities.

## Motor skill learning—constraint-induced movement therapy (supplementary table 7)

Constraint-induced movement therapy (CIMT) is a therapeutic approach that applies motor skill learning principles to stroke rehabilitation. CIMT is a specialized task-oriented training approach. Its specific strategy is to induce motor learning (practice specificity, feedback, etc.) and neuroplasticity (practice-induced brain changes arising from repetition, increasing movement complexity, motivation, and reward) with intensive blocks of training. In contrast with forced-used (solely based on the idea of immobilization of the non-paretic arm without specific intervention), CIMT requires both functional training of the affected arm with gradually increasing difficulty levels, and immobilization of the patient's non-affected upper extremity. The original high-intensity protocol of CIMT highlights: (1) repetitive task-oriented practice of the paretic upper limb for 6 h/day during 10 consecutive weekdays; (2) skills achieved in the clinical setting to be translated to the patient's daily real-world environment; (3) constraint of the non-paretic upper extremity to promote the use of the paretic upper extremity during 90% of the waking hours (Morris et al., 2006); (4) shaping (Taub et al., 2005, 2006), through consistent reward of performance thus making use of the possibility of operant conditioning (Krakauer and Shadmehr, 2006) which is an implicit or non-declarative learning process through association (Kandel et al., 2000). Modified CIMT protocols have been described with dosage regimens ranging from 0.5 to 6 h per day.

Functional neuroimaging studies suggest that increased activity in the ipsilesional sensorimotor and primary motor cortex plays a role in the improvement of functional outcome after task-specific rehabilitation (Liepert et al., 2001; Wittenberg et al., 2003; Rossini and Dal Forno, 2004; Schaechter, 2004). Alternatively, it has been suggested that motor recovery after CIMT training may occur because of a balance shift of motor cortical recruitment toward the undamaged contralesional hemisphere (Schaechter, 2004). The latter rehabilitation-induced gains may reflect a progression in the cortical processes (e.g., by unmasking existing less active motor pathways) supporting motor recovery in the early post-stroke phase (Schaechter, 2004).

The systematic review (Supplementary Table 7) yielded 33 RCTs (*n* = 1597), 1 controlled trial (*n* = 41) and 12 systematic reviews/meta-analyses (*n* = 6187).

There is moderate-quality evidence that CIMT (high intensity or modified) is superior to standard rehabilitation approaches, with regards to upper extremity impairments and disabilities. CIMT appears as beneficial in acute (with a lower dosage regimen), subacute and chronic post-stroke phases. Effects of CIMT may persist till 12 months after training. CIMT can be recommended for stroke patients after 3 months, either in its original design or in modified forms, especially if hand movement is possible. In patients without active hand movement, further studies are needed to confirm the benefit of CIMT. Under 3 months after stroke, the dosage of CIMT needs to be lowered.

One moderate-quality trial (*n* = 20) describes the application of motor skill learning outside the context of CIMT (Ausenda and Carnovali, 2011) and shows a significant improvement of hand impairments of both hands (Supplementary Table 7). Further RCTs investigating motor skill learning techniques other than CIMT, and in particular techniques studying bimanual coordination training, are needed in the adult stroke population.

Based on a sufficient amount of evidence (*n* > 500) indicating the superiority of constraint-induced movement therapy, at present, CIMT appears to be valuable and could be integrated into stroke rehabilitation strategies with a view to improving UE motor outcome (impairments and disabilities); taking into account the above-described recommendations.

Based on a lack of evidence (*n* < 500), at present, there are insufficient arguments for integrating motor skill learning techniques other than CIMT, into stroke rehabilitation with a view to improving UE motor impairments or disabilities.

## Interventions based on the hypothesis of mirror neurons and motor imagery

Original rehabilitation approaches for patients with upper extremity motor impairment have been proposed in the last decade, subtended by features of the mirror neuron system and its role in action understanding and imitation. The mirror neuron system is activated during the execution of ecological goal-directed actions, as well as during the observation of the same actions done by other individuals (Gallese et al., 1996; Rizzolatti et al., 1996; Kohler et al., 2002).

### Motor learning, movement observation, and motor imitation (supplementary table 8)

Motor learning is considered crucial for rehabilitation in general. In stroke, motor learning does not refer to the acquisition of new skills, but to the re-learning process of a previously acquired movement pattern. Stroke patients may have lost a significant portion of the brain tissue supporting the neural circuits associated with the execution or learning of movements. This situation is ideally suited for the use of observation/execution matching and motor imitation, which could provide a re-assembly of the incomplete (but not totally lost) networks (Small et al., 2012).

*Movement observation* is a passive method where participants observe another individual's motor performance. It drives the reorganization of motor representations in the primary motor cortex to form a motor memory (Stefan et al., 2005). The neural underpinnings of movement observation are thought to reside within the mirror neuron system (Fadiga et al., 1995; Ertelt et al., 2007; Garrison et al., 2010). In conventional stroke rehabilitation programs, movement observation often is used by physiotherapists for demonstrational purposes. It is easy to apply, even in severely impaired patients.

The systematic review (Supplementary Table 8) yielded 1 RCT (*n* = 102). This trial gives moderate-quality evidence indicating that movement observation is similar to a “sham” procedure with regards to UE motor impairments and disabilities (except the box and block test which was significantly better till 5 months after exposure). More RCTs are needed to ascertain this conclusion.

Based on a lack of evidence (*n* < 500), at present, there are insufficient arguments for integrating movement observation into stroke rehabilitation with a view to improving UE motor impairments or disabilities.

*Motor imitation* is a complex cognitive function that incorporates several stages, including motor observation, motor imagery and motor execution. Motor imitation-based rehabilitation approaches require patients to imitate visually perceived ecological actions. For hand motor therapy, this involves viewing complex manual tasks (e.g., using a telephone). Data show that the basic brain circuitry underlying motor imitation coincides with the circuitry active during movement observation. A direct mapping of an observed action and its motor representation seems to occur through interactions in this circuitry (Small et al., 2012).

The systematic review with the search term “motor imitation” did not yield any publications matching the inclusion criteria.

Based on a lack of evidence (*n* < 500), at present, there are insufficient arguments for integrating graded motor imitation therapies into stroke rehabilitation with a view to improving UE motor impairments or disabilities.

### Mirror therapy (supplementary table 9)

Mirror therapy was described initially as a therapeutic modality for amputee's phantom limb pain (Altschuler et al., 1999; Sathian et al., 2000). The treatment consists of a mirror being placed in the patient's midsagittal plane and reflecting the non-paretic side as if it was the affected one (Ramachandran et al., 1995). By this setup, movements of the non-paretic limb create the visual illusion of normal movements of the paretic limb (Oujamaa et al., 2009). Amongst the advantages of mirror therapy are its ease of administration, the possibility for self-administered home therapy and the applicability in patients with severe motor deficits. Some authors have described “mirror-like” video or computer graphic setups, where a video or computer graphic image of the moving limb is presented (Morganti et al., 2003; Gaggioli et al., 2004; Eng et al., 2007). The mechanisms underlying mirror therapy's effects are supposed to be related to the activity of mirror neurons which discharge in both circumstances of performing a motor act or of simply observing it being performed by another individual (Rizzolatti and Craighero, 2004; Rizzolatti and Sinigaglia, 2010). The precise mechanisms of mirror therapy in stroke patients remain speculative. It has been suggested that the mirror illusion may prevent or reverse a learned nonuse of the paretic extremity (Liepert et al., 1995) as the visual image of the paretic limb is perceived similarly to the patient's own moving limb (Dohle et al., 2004). Furthermore, mirror therapy may stimulate motor recovery directly by modulating cortical excitability.

The systematic review (Supplementary Table 9) yielded 12 RCTs (*n* = 453) and 4 systematic reviews (*n* = 1134). There is moderate-quality evidence that mirror therapy is superior to sham therapy, control therapy (task-oriented training, bimanual exercises, symmetric training) or standard rehabilitation treatment (Supplementary Table 9) with regards to upper extremity impairments and disabilities. Effects of mirror therapy may persist till 6 months after treatment. Mirror therapy appears as beneficial in acute, subacute and chronic post-stroke phases. Mirror therapy does not appear to influence upon the degree of spasticity as measured by the modified Ashworth scale.

Based on a sufficient amount of evidence (*n* > 500) indicating the superiority of mirror therapy, at present, mirror therapy appears to be valuable and could be integrated into stroke rehabilitation strategies with a view to improving UE motor impairments or disabilities.

### Mental practice with motor imagery (supplementary table 10)

Mental practice (MP) is a training method that calls for cognitive rehearsal of activities for the explicit purpose of improving performance of those activities. The movement is not actually produced but is, instead, imagined by the individual (Jackson et al., 2001; Page et al., 2001). A person participates in MP when he or she adheres to a set of imagined task performances (e.g., picking up a cup) or movements (e.g., reaching out with his or her arm). This visualization may occur from the first person or third person perspective, and the protocol defines either the number of imagined repetitions or the amount of time the individual invests in the imagining procedure. The imagined movements or tasks are performed without external visual cueing (e.g., watching performance on a videotape) although the training of the imagined procedure may use this modality (Barclay-Goddard et al., 2011). Mental practice can be combined with physical practice or used by itself.

Hypotheses have been proposed to explain how MP works. The “neuromuscular theory” (Schmidt and Lee, 1999) hypothesizes that an individual engaged in MP repeatedly activates the desired motor program but with the “gain” of the program dampened, thereby rendering the muscle contractions so weak that no movement is observed. Nonetheless, the individual's learning improves from these subthreshold activations of motor programs. Another explanation is that individuals engaged in MP rehearse elements of the task giving thereby the opportunity to predict outcomes of actions based on their previous experience. They thus develop ways to address the outcomes and anticipate courses of actions that they were more likely to use during the real execution of the movement. Functional neuroimaging studies suggest a reorganization of the brain motor network for the unaffected as well as for the affected hemisphere, thus improving the regional connectivity among the motor areas (Bajaj et al., 2015a,b).

The systematic review (Supplementary Table 10) yielded 5 RCTs (*n* = 228) and 5 systematic reviews/meta-analyses (at least *n* = 1266). There is moderate-quality evidence that mental practice with motor imagery in combination with another rehabilitation treatment is superior to the other rehabilitation treatment alone with regards to upper extremity impairments and disabilities. Mental practice with motor imagery appears as beneficial in the subacute and chronic post-stroke phase.

Based on a sufficient amount of evidence (*n* > 500) indicating the superiority of mental practice with motor imagery, at present, mental practice with motor imagery appears to be valuable and could be integrated into stroke rehabilitation strategies with a view to improving UE motor impairments or disabilities.

## Adjuvant therapies

In combination with previously described neurorehabilitation concepts, some complementary techniques may allow potentiating the patient's recovery. Different adjuvant therapies are proposed in the literature and will now be discussed.

### Electrical stimulation of the paretic arm

Therapeutic electrical stimulation after stroke can be divided into two types: (a) sensory electrical stimulation; (b) muscle (or motor) electrical stimulation.

#### Sensory electrical stimulation: high-frequency transcutaneous electrical nerve stimulation (TENS) and electroacupuncture (supplementary table 11)

TENS corresponds to the electrical somatosensory stimulation of a peripheral nerve through the use of cutaneous electrodes. In function of the stimulation, TENS is categorized into high-frequency TENS and low-frequency TENS corresponding to respective stimulation frequencies of 80–100 and 1–5 Hz. High-frequency TENS elicits sensory responses, whereas low-frequency TENS may elicit motor contractions as well. For the latter reason, literature on low-frequency TENS will be discussed in the following section (Muscle electrical stimulation). Electroacupuncture is an electrical stimulation technique based on the application of electrical current at low frequencies (2–3 Hz) during acupuncture needling. The exact mechanisms of action of TENS on motor recovery after stroke are unknown. Most likely, a long-term potentiation-like mechanism in the excitatory glutamatergic connections between the primary sensory and motor cortices mediates the direct effects of repetitive transcutaneous electrical nerve stimulation on corticospinal excitability and motor performance (for review: Veldman et al., 2014).

The systematic review (Supplementary Table 11) yielded seven RCTs (*n* = 347) and 1 systematic review (*n* = 446). There is moderate-quality evidence that high-frequency TENS (100 Hz) in combination with rehabilitation treatment is superior to the rehabilitation treatment alone with regards to upper extremity impairments and disabilities. High-frequency TENS appears as beneficial in the subacute and chronic post-stroke phase. Spasticity appears to diminish with high frequency-TENS.

There is moderate-quality evidence that electroacupuncture (2–3 Hz) in combination with rehabilitation treatment is superior to the rehabilitation treatment alone with regards to upper extremity impairment. Further RCTs are needed to ascertain this conclusion.

Based on a sufficient amount of evidence (*n* > 500) indicating the superiority of high-frequency TENS, at present, high-frequency TENS appears to be valuable and could be integrated as an adjuvant therapy into stroke rehabilitation strategies with a view to improving UE motor impairments and disabilities.

Based on a lack of evidence (*n* < 500), at present, there are insufficient arguments for integrating electroacupuncture as an adjuvant therapy into stroke rehabilitation with a view to improving UE motor impairments or disabilities.

#### Muscle electrical stimulation: low-frequency transcutaneous electrical nerve stimulation and neuromuscular electrical stimulation (un-triggered/simple/passive, EMG-triggered or positional feedback) (supplementary table 12)

Muscle contractions can be elicited by electrical stimulation through surface skin electrodes. Low-frequency TENS over a peripheral nerve induces muscle contractions at stimulation frequencies of 1–5 Hz. Neuromuscular electrical stimulation (NMES) over a muscle (neuromuscular endplate) induces muscle contractions at stimulation frequencies of 10–50 Hz. NMES can be used to elicit simple muscle contractions as a passive technique or can be actively triggered by electromyographic activity (EMG-NMES) or by limb position (position-triggered NMES) (for review: Schuhfried et al., 2012). The two forms of triggered electrical stimulation increase the active participation of the stroke patients in upper extremity task-oriented training. Electromyogram-triggered electrical stimulation combines electromyographic biofeedback with the delivery of electrical stimulation. When the stroke patient attempts the task and the EMG signal of the voluntary contraction exceeds a preset threshold, electrical stimulation is delivered to the target muscle to develop movement through to full range (Francisco et al., 1998; Bolton et al., 2004). This treatment modality is indicated in stroke patients who can voluntarily activate the paretic muscles (at least 2/5 on Medical Research Council scale), but are unable to generate sufficient muscle activation to achieve a movement goal (Francisco et al., 1998). Positional feedback stimulation works on the same pretense as EMG feedback, but relies on the angle of the upper extremity to trigger stimulation, rather than the EMG signal (Bowman et al., 1979).

The systematic review (Supplementary Table 12) yielded 17 RCTs (*n* = 790) and 4 systematic reviews (*n* = 2293).

There is moderate quality evidence that low-frequency TENS (2 Hz) in combination with rehabilitation treatment is superior to the rehabilitation treatment alone with regards to upper extremity impairment. There is no effect of low-frequency TENS on UE disabilities. Low-frequency TENS does not appear to influence upon spasticity. Further RCTs are needed to ascertain this conclusion.

There is moderate-quality evidence that simple/passive NMES in combination with rehabilitation is superior to the rehabilitation treatment alone with regards to upper extremity impairment (strength, range of motion). There is no effect of simple/passive NMES on UE disabilities. Treatment effects have been described in acute and subacute stroke patients. Simple/passive NMES does not appear to influence upon spasticity.

There is moderate quality evidence that EMG-NMES in combination with rehabilitation treatment is similar to the rehabilitation treatment alone or to passive NEMS with regards to upper extremity impairment (strength, range of motion, grip-lift task). However, it is difficult to dissociate EMG-NMES' effects from those of the rehabilitation treatment. There is no effect of EMG- NMES on UE disabilities. Further RCTs are needed to explore the efficacy of EMG-triggered neuromuscular electrical stimulation.

The systematic review with the search term “positional feedback” did not yield any publications matching the inclusion criteria.

Based on a sufficient amount of evidence (*n* > 500) indicating the superiority of passive neuromuscular electrical stimulation, at present, passive NMES appears to be valuable and could be integrated as an adjuvant therapy into stroke rehabilitation strategies with a view to improving UE motor impairments.

Based on a lack of evidence (*n* < 500), at present, there are insufficient arguments for integrating low-frequency TENS, EMG-NMES or positional feedback-NMES as an adjuvant therapy into stroke rehabilitation with a view to improving UE motor impairments or disabilities.

### Non-invasive brain stimulation

Repetitive transcranial magnetic stimulation (rTMS) and transcranial direct current stimulation (tDCS) influence the function of the corticospinal tracts by modulating the corticomotor excitability (Nitsche and Paulus, 2000, 2001; Hummel and Cohen, 2006). In post-stroke patients, abnormal levels of inter-hemispheric inhibition are found to be exerted by the unaffected on the affected motor cortex (Hummel and Cohen, 2006). rTMS and tDCS as non-invasive neuromodulatory therapies have been studied in stroke recovery (for a review: Adeyemo et al., 2012).

#### rTMS (supplementary table 13)

Transcranial magnetic stimulation is a painless, non-invasive technique. The rapidly changing magnetic field initiated by a brief high intensity electric current, passes through a coil over the scalp. It can be delivered via a single pulse, double pulses, paired pulses, and repetitive pulses. The number of sessions is most often one daily session during 5–10 consecutive days. rTMS induces repetitive electrical currents in the brain cortex resulting in long-term changes of the cortical excitability which last beyond the stimulation time (Adeyemo et al., 2012). When the rTMS stimulation frequency is low (1 Hz), the cortical excitability is diminished whereas when the rTMS stimulation frequency is high (3–10 Hz), excitatory effects are obtained. In a rat model, there is evidence that high-frequency rTMS may decrease apoptosis after stroke (Gao et al., 2010). In humans, low-frequency rTMS (inhibitory stimulation) to the unaffected hemisphere could normalize the inhibitory imbalance between hemispheres (Adeyemo et al., 2012). Theta-burst stimulation (TBS) is a specific protocol of rTMS using higher stimulation frequencies (3 pulses at 50 Hz) in an intermittent or in a continuous way, and is considered to suppress cortical activity. The safety and application guidelines of transcranial magnetic stimulation were extensively reviewed by Rossi et al. (2009). Among recent non-invasive stimulation techniques, paired associative stimulation (PAS) introduced by Stefan et al. (2000), consists of repetitive pairing of a peripheral nerve with a non-invasive cortical stimulation achieved by transcranial magnetic stimulation. PAS results in a potentiation of corticospinal excitability lasting 30–60 min beyond the stimulation procedure (Lamy et al., 2010).

The systematic review (Supplementary Table 13) yielded 20 RCTs (*n* = 663), 4 controlled trials (*n* = 97) and 5 systematic reviews (*n* = 1173).

There is moderate-quality evidence that rTMS (alone, not as an adjuvant treatment) is superior to sham rTMS with regards to improving upper extremity impairments. There is no effect of rTMS alone on UE disabilities. Differential effects on UE impairment are obtained according to the type of rTMS that is used (for details: Supplementary Table 13). Long-lasting effects have been obtained on UE impairment up to 1 year after treatment in acute stroke patients. There is moderate- to high-quality evidence that rTMS in combination with another rehabilitation treatment (occupational therapy, physiotherapy, motor training) potentiates the effect of the rehabilitation treatment alone with regards to UE impairment. No evidence has shown an effect of the combined treatment (rTMS + conventional rehabilitation) on UE disabilities. Treatment effects have been described in acute, subacute and chronic stroke patients. Two studies suggest that spasticity may diminish when rTMS is used in combination with either physiotherapy or functional electrical stimulation.

There is moderate- to high-quality evidence that theta-burst stimulation in combination with rTMS or with rehabilitation treatment is superior to sham TBS with regards to upper extremity impairment. There is no effect of TBS on UE disabilities. No evidence is available on the effects of TBS on spasticity. TBS has been studied in chronic stroke patients and evidence in acute or subacute stroke patients is lacking.

The systematic review with the search term “paired associative stimulation” did not yield any publications matching the inclusion criteria.

Based on a sufficient amount of evidence (*n* > 500) indicating the superiority of repetitive transcranial magnetic stimulation, at present, rTMS appears to be valuable and could be integrated as an adjuvant therapy into stroke rehabilitation strategies with a view to improving UE motor outcome (impairments, not disabilities), taking into account safety guidelines and the differential effects of stimulation protocols.

Based on a lack of evidence (*n* < 500), at present, there are insufficient arguments for integrating theta-burst stimulation or paired associative stimulation as an adjuvant therapy into stroke rehabilitation with a view to improving UE impairments or disabilities.

#### tDCS (supplementary table 14)

tDCS is a noninvasive application of weak electrical current to brain tissue. It can be used to manipulate the membrane potential and modulate spontaneous firing rates of neurons in animals and humans (Nitsche and Paulus, 2000). Neuromodulation by tDCS in stroke patients with hemiplegia aims at reducing interhemispheric imbalance and improving brain plasticity (Kandel et al., 2012). tDCS can be applied in several montages: (1) anodal stimulation, with the anodal electrode placed over the affected hemisphere; (2) cathodal stimulation with the cathodal electrode placed over the unaffected hemisphere; (3) bihemispheric stimulation (dual tDCS), combining anodal and cathodal stimulation respectively on the affected and unaffected hemisphere (Schlaug and Renga, 2008).

Several thousand subjects have been stimulated with tDCS without reporting any severe adverse events (Nitsche et al., 2008). Minor side effects of tDCS are well documented (Poreisz et al., 2007) and consist of a sensation of tingling or rash at the electrode site (temporarily at the beginning of the stimulation) or an erythematous skin rash (due to vasodilatation). Safety guidelines for using tDCS have been described by Nitsche et al. (2003) and by Bikson et al. (2009).

The systematic review (Supplementary Table 14) yielded 14 RCTs (*n* = 482) and 4 systematic reviews/meta-analyses (at least *n* = 455).

There is moderate- to high-quality evidence that tDCS (alone, not as an adjuvant treatment) is superior to sham tDCS with regards to improving upper extremity impairment. There is no effect of tDCS alone on UE disabilities. Differential effects on UE impairments are obtained according to the type of tDCS that is used (for details: Supplementary Table 14). Effects of tDCS are observed till 1 week after treatment. There is moderate- to high-quality evidence that tDCS in combination with rehabilitation treatment (occupational therapy, physiotherapy, motor training, task-specific training) potentiates the effect of the rehabilitation treatment alone with regards to UE impairments. Follow-up studies indicate heterogeneous results on UE disabilities at 3 months after acute stroke (Hesse et al., 2011; Khedr et al., 2013). Treatment effects have been described in acute, subacute and chronic stroke patients.

Based on a sufficient amount of evidence (*n* > 500) indicating the superiority of transcranial direct current stimulation, at present, tDCS appears to be valuable and could be integrated as an adjuvant therapy into stroke rehabilitation strategies with a view to improving UE motor outcome (impairments, not disabilities), taking into account safety guidelines and the differential effects of stimulation protocols.

### Invasive brain stimulation

The systematic review with the search term “deep brain stimulation” did not yield any publications matching the inclusion criteria.

Based on a lack of evidence (*n* < 500), at present, there are insufficient arguments for integrating deep brain stimulation as an adjuvant therapy into stroke rehabilitation with a view to improving UE impairments or disabilities.

### Drugs used for stimulating motor recovery

#### Antidepressants (supplementary table 15)

Beyond their ability to improve mood disturbances following stroke, antidepressants can be used to enhance upper extremity motor recovery through their influence on brain neurotransmission. Selective serotonin reuptake inhibitors (SSRI) and noradrenaline reuptake inhibitors (NARI) are the best studied drugs in stroke patients. Other types of drugs have also been assessed for their effects on upper extremity paresis: stimulants (amphetamines and methylphenidate), dopaminergics (levodopa), methylphenidate, trazadone, and nortriptyline (for review: Berends et al., 2009). However, at present there is insufficient evidence to discuss the efficacy of these latter drugs.

The systematic review (Supplementary Table 15) yielded 6 RCTs (*n* = 361), 1 controlled study (*n* = 64), 1 case-control study (*n* = 508) and 2 systematic reviews/meta-analyses (*n* = 5380). There is moderate- to high-quality evidence that antidepressant therapy by SSRIs or NARIs in combination with conventional rehabilitation treatment (occupational therapy, physiotherapy, speech therapy) potentiates the effect of the rehabilitation treatment alone with regards to UE impairments or disabilities. Treatment effects have been described in acute and subacute stroke patients.

Based on a sufficient amount of evidence (*n* > 500) indicating the superiority of antidepressants drugs, at present, antidepressant drug therapy appears to be valuable and could be integrated as an adjuvant therapy into stroke rehabilitation strategies with a view to improving UE motor outcome (impairments and disabilities), in depressed as well as undepressed acute stroke patients.

#### Botulinum toxin (supplementary table 16)

Spasticity occurring after stroke is a velocity-dependent increase in muscle tone due to loss or dysfunction of upper motor neurons. It can develop as early as 1 week after stroke and occurs in up to 50% of stroke patients. In the long term, spasticity provokes tendon contractures and limb deformities causing significant pain and functional impairment (Kaku and Simpson, 2016). The intramuscular injection of botulinum toxin is considered as an efficient treatment to decrease UE spasticity. Different techniques to inject botulinum toxin and different types of botulinum toxin have been described which may result in different outcomes (for review: Chan et al., 2016; Kaku and Simpson, 2016).

The systematic review (Supplementary Table 16) yielded 17 RCTs (*n* = 1583), 1 controlled study (*n* = 59) and 4 systematic reviews (*n* = 4456). There is moderate- to high-quality evidence that botulinum toxin is superior to placebo treatment with regards to UE impairment (spasticity). However, no effect is observed on UE disabilities. There is moderate- to high quality evidence that botulinum toxin in combination with rehabilitation treatment (mCIMT, multidisciplinary rehabilitation, physiotherapy) is superior to placebo treatment in combination with rehabilitation treatment with regards to UE impairment (spasticity). Any effect on UE disabilities appears to depend on the type of concomitant rehabilitation treatment, and not on botulinum toxin itself. Short-term (1–3 months) treatment effects of botulinum toxin on spasticity have been described in acute, subacute and chronic stroke patients.

Based on a sufficient amount of evidence (*n* > 500) indicating the superiority of botulinum toxin injection, at present, botulinum toxin appears to be valuable by itself with a view to improving UE motor impairment (spasticity) and could be integrated as an adjuvant therapy into stroke rehabilitation strategies with a view of improving UE motor disabilities.

## Technology supported training

For many health professionals working in stroke rehabilitation, the future lies within the development of technology-supported training for upper extremity recovery. Promising new technologies will be discussed in the light of current evidence for their use in clinical settings.

### Robot-assisted therapy for the paretic upper extremity (supplementary table 17)

A robot is defined as a re-programmable, multi-functional manipulator designed to move material, parts or specialized devices through variable programmed motions in order to accomplish a task (Pignolo, 2009). An increasing number of robotic devices have become available for post-stroke rehabilitation (Stein, 2012). Robotic therapy used for upper extremity rehabilitation combines three basic components: (1) a motorized mechanical component to which the hand is attached that provides passive, active-assisted or active-resisted movement of the hand to the target; (2) performance-related visual feedback via a screen; (3) an interactive computer program that monitors and incrementally progresses the training such as to motivate the stroke patient (Fasoli et al., 2004; Hidler et al., 2005). The main advantages of using robot-assisted therapy are to deliver high-dosage and high-intensity training (Sivan et al., 2011). Most robotic devices are tailored for elbow and shoulder movements. There is a lack of robotic training devices for finger and wrist movements. Existing upper extremity robotic systems can be classified in passive systems (stabilizing limb), active systems (actuators moving limb) and interactive systems (for review: Riener et al., 2005). Upper extremity robotic interactive systems can be classified by the degrees of freedom (DOF) in which they allow movement to occur or by the type of skeleton (end-effector vs. exoskeleton; for review: Chang and Kim, 2013).

The systematic review (Supplementary Table 17) yielded 11 RCTs (*n* = 478), 1 controlled trial (*n* = 47) and 6 systematic reviews (*n* = 2587). There is moderate-quality evidence that robot-assisted therapy for the paretic UE is similar or inferior to standard rehabilitation treatment. Any gains that are obtained are specific to the task that is being trained (motor impairment) and do not extrapolate to upper extremity disabilities in daily life. The efficacy of robot-based therapy of the paretic upper extremity does not appear to be specific for a post-stroke phase.

Based on a sufficient amount of evidence (*n* > 500) indicating the non-superiority of robot-assisted therapy, at present, there are insufficient arguments for integrating robot-assisted therapy for the paretic upper extremity into stroke rehabilitation with a view to improving UE motor impairments or disabilities.

### Virtual reality, virtual reality immersion, and gaming (supplementary table 18)

Virtual reality computerized techniques allow subjects to interact with a virtual environment. Task-oriented training with robotic devices (as discussed in the section robot-based arm therapy) frequently is based on the interaction with a two-dimensional virtual environment presented on a computer or television screen.

Virtual reality immersion techniques are based on the conjunct use of a computer-generated three-dimensional graphical environments (Riva, 2003; Oujamaa et al., 2009) and visual, auditory, or haptic devices. The operator is supposed to experience the computer-generated environment as if it were a part of the real world. Users can interact with a virtual environment through the use of standard input devices such as a keyboard and mouse, or through multimodal devices such as a wired glove. Because of the playful aspect of the training, subjects tend to be more motivated in virtual reality settings than in conventional rehabilitation settings (Jang et al., 2005).

The so-called “serious gaming” may increase patient's adherence and self-management, aid physical and psychological recovery, and enhance patient's and clinician's knowledge in a range of contexts (Kato et al., 2008). Gaming literature emphasizes its potential to increase: patient motivation, learning through repetition in an enriched environment, confidence through reinforcement and immediate feedback, and positivity through achievement and social interaction (Krichevets et al., 1995; Fitzgerald et al., 2004).

The systematic review (Supplementary Table 18) yielded 10 RCTs (*n* = 697) and 4 systematic reviews (*n* = 760). There is moderate-quality evidence that virtual reality is similar to standard rehabilitation treatment with regards to UE impairment and disabilities. There is moderate-quality evidence that virtual reality combined with another rehabilitation treatment (tDCS, conventional rehabilitation) is superior to the other rehabilitation treatment alone with regards to UE impairments and activities. Treatment effects have been described in chronic stroke patients.

There is moderate-quality evidence that virtual immersion is superior to standard rehabilitation treatment with regards to UE impairment and disabilities (only two RCTs available). There is moderate-quality evidence that serious gaming is superior to standard rehabilitation treatment or recreational therapy with regards to UE impairment (only two RCTs available). However, further RCTs are needed to ascertain treatment effects of virtual immersion and serious gaming.

Based on a lack of evidence (*n* < 500), at present, there are insufficient arguments for integrating virtual reality (without another rehabilitation treatment), virtual immersion or serious gaming into stroke rehabilitation with a view to improving UE motor impairments or disabilities.

Based on a sufficient amount of evidence (*n* > 500) indicating the superiority of virtual reality as an adjuvant therapy, at present, virtual reality combined with another rehabilitation treatment appears to be valuable and could be integrated as an adjuvant therapy into stroke rehabilitation strategies with a view to improving UE motor impairment and disabilities.

### Music-supported therapy (MST) (supplementary table 19)

Neurologic music therapy (NMT) aims at improving cognitive, sensory and motor function in neurological patients through the therapeutic application of music. Passive music-supported therapy includes auditory-motor synchronization, an entrainment function with rhythmic auditory cueing of movement execution as well as motivational aspects (Mitchell et al., 2008; Thaut et al., 2008). Active music-supported therapy uses musical instruments or specifically designed haptic devices to train fine and gross movements of the paretic upper extremity (Rodriguez-Fornells et al., 2012).

The systematic review (Supplementary Table 19) yielded 2 RCTs (*n* = 74) and 2 systematic reviews (reporting the same results on *n* = 41). There is moderate quality evidence that passive music-supported therapy is similar to standard rehabilitation treatment with regards to UE impairment. There is moderate quality evidence that active-music supported therapy is superior to standard rehabilitation treatment with regards to UE impairment. Further RCTs are needed to ascertain these conclusions. Treatment effects have been described in acute, subacute and chronic stroke patients.

Based on a lack of evidence (*n* < 500), at present, there are insufficient arguments for integrating passive or active music-supported therapy into stroke rehabilitation with a view to improving UE motor impairments or disabilities.

## General discussion

**Figure 4:**
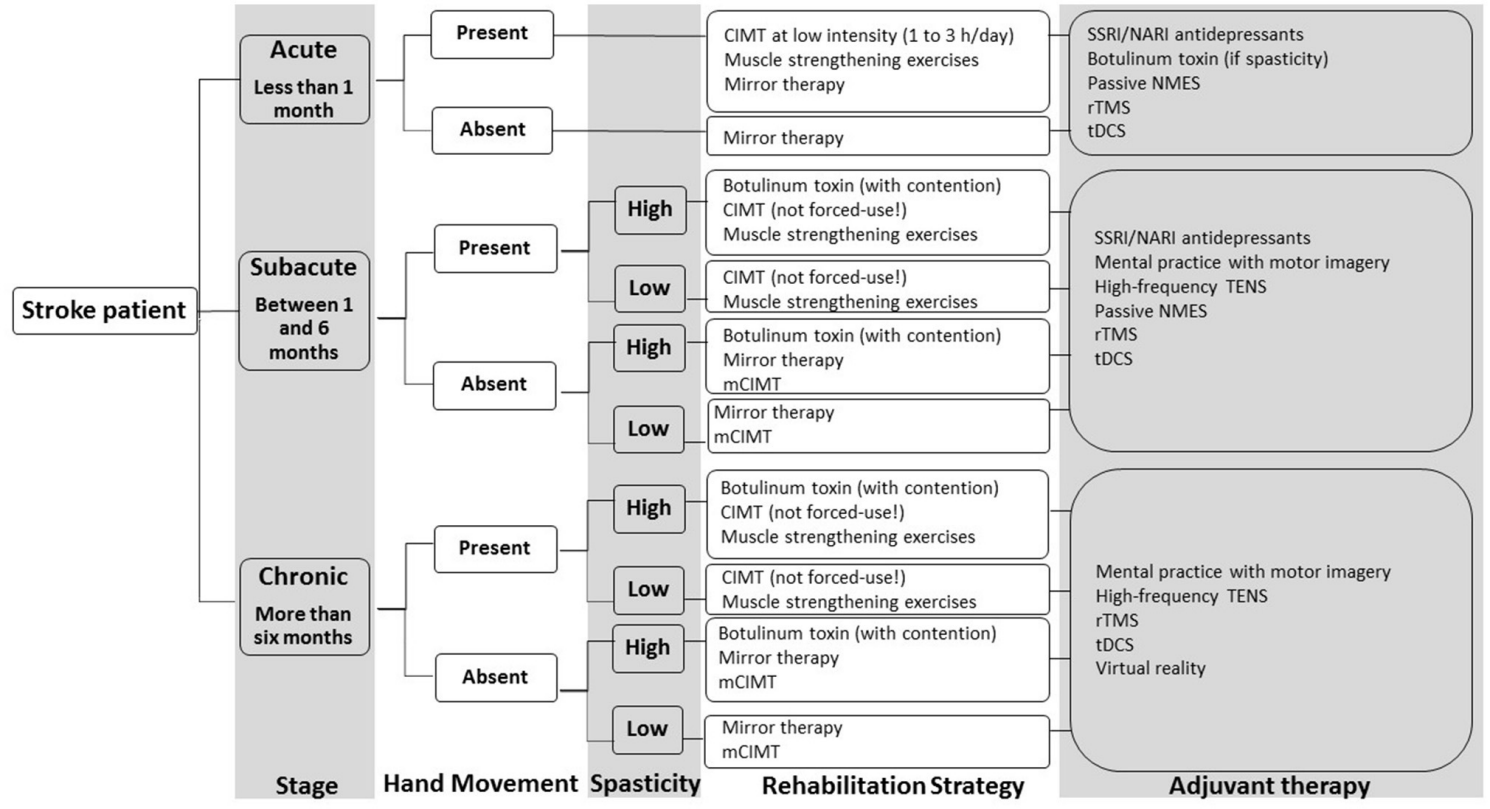
**Decisional tree for upper extremity rehabilitation after stroke based on the conclusions of the multiple systematic review**. Abbreviations: SSRI, selective serotonin reuptake inhibitor; NARI, noradrenalin reuptake inhibitor; CIMT, constraint-induced movement therapy; mCIMT, modified constraint-induced movement therapy; tDCS, transcranial direct current stimulation; rTMS, repetitive transcranial magnetic stimulation, NMES, neuromuscular electrical stimulation, TENS, transcutaneous electrical nerve stimulation.

This review focused on rehabilitation techniques stimulating motor recovery of the upper extremity after stroke. Neurorehabilitation approaches were divided into six different chapters, as well as discussed and recommended on the basis of current scientific evidence. A total number of 5712 publications on stroke rehabilitation was systematically reviewed for relevance and quality with regards to upper extremity motor outcome. This procedure yielded 270 publications corresponding to the inclusion criteria of the systematic review. Based on the current level of evidence for each rehabilitation intervention, a decisional tree for upper extremity rehabilitation after stroke is proposed as a clinical tool for choosing a specific patient's intervention (Figure 4). The decisional tree is based on the stage of stroke, the presence of hand movement and the presence of spasticity. In function of these three patient's characteristics (stage, hand movement, spasticity), specific rehabilitation approaches as well as adjuvant rehabilitation techniques are recommended.

The main findings of this multiple systematic review concerning rehabilitation techniques focusing on the UE motor outcome, may be summarized as follows.

Rehabilitation approaches recommended as a main rehabilitation intervention on the basis of current evidence for improving UE motor outcome, are: muscle strengthening exercises (impairments), constraint-induced movement therapy (impairments and disabilities), mirror therapy (impairments and disabilities), botulinum toxin (as an intervention *per se*: impairments).

Rehabilitation approaches recommended as an adjuvant therapy (combined with another rehabilitation treatment) on the basis of current evidence for improving UE motor outcome, are: mental practice with motor imagery (impairments and disabilities), high frequency-transcutaneous electrical nerve stimulation (impairments and disabilities), passive neuromuscular electrical stimulation (impairments), repetitive transcranial magnetic stimulation (impairments), transcranial direct current stimulation (impairments), SSRI and NARI antidepressants (impairments and disabilities), botulinum toxin (as an adjuvant intervention: disabilities), virtual reality (impairments and disabilities).

Rehabilitation approaches that are not recommended on the basis of current evidence because scientific data do not show their efficacy for UE motor outcome, are: Bobath concept, manual passive stretching, bilateral training (device- or non-device-assisted, task-oriented), robot-assisted therapy for the paretic upper extremity (task-oriented).

Rehabilitation approaches that are not recommended on the basis of current evidence because there is insufficient scientific data available with regards to UE motor outcome, are: Perfetti method, Picard method, isokinetic muscle strengthening, device-assisted stretching (contention, splint, cast, taping), motor skill learning techniques (other than CIMT), movement observation, motor imitation, electroacupuncture, low-frequency TENS, electromyography-triggered neuromuscular electrical stimulation, position-triggered neuromuscular electrical stimulation, theta-burst stimulation, paired associative stimulation, deep brain stimulation, virtual immersion, serious gaming, passive music-supported therapy, active music-supported therapy. Large RCTs are needed to confirm preliminary data in these fields.

According to the stage of stroke, some rehabilitation concepts may be more appropriate than others. In acute stroke patients, the following rehabilitation approaches have been studied and are recommended: muscle strengthening exercises, constraint-induced movement therapy (with a lower dosage regimen), mirror therapy, passive neuromuscular electrical stimulation, repetitive transcranial magnetic stimulation, transcranial direct current stimulation, SSRI and NARI antidepressants, botulinum toxin.

In subacute stroke patients, the following rehabilitation approaches have been studied and are recommended: muscle strengthening exercises, constraint-induced movement, mirror therapy, mental practice with motor imagery, high frequency-transcutaneous electrical nerve stimulation, passive neuromuscular electrical stimulation, repetitive transcranial magnetic stimulation, transcranial direct current stimulation, SSRI and NARI antidepressants, botulinum toxin.

In chronic stroke patients, the following rehabilitation approaches have been studied and are recommended: muscle strengthening exercises, constraint-induced movement therapy, mirror therapy, mental practice with motor imagery, high frequency-transcutaneous electrical nerve stimulation, repetitive transcranial magnetic stimulation, transcranial direct current stimulation, botulinum toxin, virtual reality.

For some neurorehabilitation approaches, the severity of initial motor deficit may impact upon the feasibility and effectiveness of the intervention. This is apparent for muscle strengthening exercises, constraint-induced movement therapy and virtual reality interfaces.

The following neurorehabilitation approaches may modulate the degree of spasticity: botulinum toxin (with or without physical contention), and in a lesser way: repetitive transcranial magnetic stimulation, high frequency-transcutaneous electrical nerve stimulation and transcranial direct current stimulation.

The following neurorehabilitation approaches that are effective upon the UE motor outcome, do not impact upon the degree of spasticity: muscle strengthening exercises, passive neuromuscular electrical stimulation, mirror therapy, constraint-induced movement therapy, virtual reality.

The decisional tree proposed in this manuscript (Figure 4) is based on the current scientific evidence as found in this multiple systematic review. At present, it reflects how scientific data should underpin the rehabilitation strategy after stroke and how clinical rehabilitation interventions can be chosen in function of an individual patient's characteristics. Specific reasons may exclude a patient from the proposed treatment strategy. As an example, before starting non-invasive brain stimulation safety issues need to be considered in function of the medical history and medical status of the patient.

This systematic review may present some limitations. Though the investigators aimed at providing a large overview of current rehabilitation techniques for the UE, the specified choice of search terms may have excluded clinical rehabilitation strategies that are unusual in Western-European countries. This review does not include some recent technological advances making their way into clinical rehabilitation such as brain-computer interface based technologies (for review: Soekadar et al., 2015; van Dokkum et al., 2015; Remsik et al., 2016) and functional electrical stimulation of the upper extremity (for review: Quandt and Hummel, 2014; Vafadar et al., 2015). It also has to be acknowledged that the methodology of this “multiple” systematic reviews paper allowed to include techniques that are unfrequently reported in reviews because of a lack of RCTs or SR (examples: music therapy, motor skill learning, isokinetic muscle strengthening, paired associative stimulation, theta burst stimulation). Thus, this qualitative systematic review may have reported effects in fields where few studies are published. Therefore, a very conservative line was adopted with regards to the recommendations. The recommendations on each rehabilitation intervention depended on the average quality of data, the total amount of evidence (number of subjects included in selected studies) and the average qualitatively reported results of trials (see Methods Section). Long term effects of stroke rehabilitation could not be described in detail in this systematic review as they have been investigated in few publications and need to be clarified in future RCTs and meta-analyses. Some of the rehabilitation concepts that are discussed in the present paper may be effective on neurological outcomes other than motor recovery of the UE. Thus, results of the present paper always should be discussed in the light of the inclusion criteria and methodology of the systematic search.

Overall, evidence of this “multiple” systematic review indicated that the functional recovery from stroke is positively influenced by goal-specific sensorimotor input through training or everyday use of the arm and hand. Task-oriented training optimizes the UE motor function related to the targeted motor task (“you gain what you train”), but subsequent improvements of motor impairment do not transfer to improving motor disabilities in activities of daily living. As an example, the lack of effectiveness of bilateral arm training (non-goal-oriented repetitive task movements) stands in contrast with the significant improvement of motor impairments and disabilities by constraint-induced movement therapy applying the premises of goal-oriented motor skill learning techniques. It can be hypothesized that a functional bimanual intensive training without constraint (as has been described in children with congenital hemiplegia, Charles and Gordon, 2006; Gordon et al., 2007) could be a future pathway for adult stroke neurorehabilitation research. It also seems that the impact of rehabilitation technology on functional outcome could be optimized by offering more chances to the nervous system to experience “real” and repetitive activity-related adequate sensory-motor input during training of upper limb movement, instead of task-specific exercises.

To conclude, many clinical and research interventions are available to promote upper extremity motor function in stroke patients. Furthermore, interventions can be combined in order to achieve the maximal motor function recovery for each patient. At present, the stroke rehabilitation field faces the challenge to tailor training to the needs of the individual stroke patient. Though the effects of some interventions are under debate, some specific rehabilitation approaches give promising motor outcome prognosis for the upper extremity after stroke.

## Author contributions

SH chose the research's subject, determined the methodology of the systematic review, chose the search terms, performed the systematic search, performed and supervised the systematic review and wrote and reviewed the manuscript. GS performed the systematic review, checked the reference list, and compiled the abbreviations' list. MD performed the systematic review. VP chose the search terms and participated in writing the manuscript. XZ participated in writing the manuscript. DD chose the search terms and participated in writing the manuscript. YB determined the methodology of the systematic review, chose the search terms, performed the systematic search, performed and supervised the systematic review and participated in writing the manuscript.

## Funding

SH is supported by Fonds De La Recherche Scientifique—FNRS (Belgium) as a postdoctorate clinical master specialist.

### Conflict of interest statement

The authors declare that the research was conducted in the absence of any commercial or financial relationships that could be construed as a potential conflict of interest.
