# Supplementary material for: Rehabilitation of Motor Function after Stroke: A Multiple Systematic Review Focused on Techniques to Stimulate Upper Extremity Recovery
Source: Front Hum Neurosci. 2016 Sep 13;10:442. doi: 10.3389/fnhum.2016.00442 (PMC5020059; doi:10.3389/fnhum.2016.00442)
Supplement: Supplementary file 1 [file DataSheet1.docx]

**Supplementary file:**

**Exercise therapy**

The key principle of most rehabilitation strategies is to induce adaptive brain plasticity by providing repetitive, gradual, adequate and intense modulatory input to influence cortical reorganization as early as possible after stroke onset (Langhorne et al., 2011). Some rehabilitation strategies such as stretching or strength training, aim at modulating peripheral mechanisms as well.

Current clinical programs of stroke rehabilitation combine a variety of motor interventions such as the repeated practice of everyday tasks, constraint-induced movement therapy (CIMT), bilateral arm training (BAT), electrical stimulation, mirror therapy, mental practice/rehearsal, biofeedback, robot-assisted arm training, Bobath approach, etc. These motor rehabilitation strategies may be associated with diverse adjunctive treatments such as functional electrical stimulation compensating for motor command deficit, the injection of botulinum toxin for spasticity or prismatic adaptation for neglect syndrome. In the early rehabilitation phase, preventive measures such as therapeutic positioning of the arm, strapping to avoid glenohumeral subluxation and shoulder pain, air splints to reduce oedema and upper limb orthoses to prevent and manage length-associated changes in muscles and connective tissue, may prove crucial in maintaining adequate passive mobility of the upper limb while awaiting recovery of voluntary motor control (Veerbeek et al., 2014).

Exercise therapy is the basis of stroke rehabilitation and will therefore be discussed in depth in this supplementary material file. Exercises performed after stroke, differ by their objective (goal-directed, task-oriented, repetitive task training) and by their technical characteristics (duration, training load and type of feedback).

Goal-directed approach

A goal-directed approach of therapeutic exercise includes several essential components: 1) the selection of the patient's goal from a choice that is guided to be "SMART" (= Specific, Measurable, Attainable, Realistic and Time specified), 2) the analysis of the performance regarding the selected goal, 3) the identification of variables that limit the performance and the identification of the patient’s constraints as a basis for selecting the treatment strategy, 4) the analysis of the intervention and patient's performance leading to structurally offered feedback that supports motor learning, 5) the conscious involvement of the subject to learn from feedback via restoration of cognitive processes that are associated with functional movement and 6) the finding of strategies to determine individually the most effective solutions (Mastos et al., 2007). Goal attainment scaling (GAS) is an efficient tool for the above described process and evaluation of training outcome. Indeed, the patient together with the therapist define a goal as well as a range of possible outcomes for it, on a scale from 0 (expected result) +/- 2. This implies that progresses are rated relative to the goal set at baseline (Kiresuk et al., 1994; Mastos et al., 2007). “Client-centered” training increases the level of “active” participation of the patient in the rehabilitation process (Wressle et al., 2002). This has a positive influence on patient motivation which is an important factor for motor learning, as attention during training is enhanced and exercise repetition and treatment compliance are stimulated (Timmermans et al., 2009).

Task-oriented training

“Task-oriented training”, also named task-specific training, goal-directed training or functional task practice is, at present, a poorly defined concept (Timmermans et al., 2010; Timmermans et al., 2009; Van Peppen et al., 2004). Some studies consider single-joint and/or single-plane movement exercises to be task-oriented training (e.g. reaching, pointing) (Cirstea et al., 2006) whereas other studies consider task-oriented training to focus on meaningful complex movements with real-life object manipulation and environment (i.e. drinking coffee or walking) (Baskett et al., 1999). Task-oriented training addresses different levels of the International Classification of Functioning and Handicap (ICF) as are function, activity and participation levels (World Health Organization, 2001). Patients should be actively involved in the participation and the tasks should be challenging and progressively adaptive.  Subjects learn by solving problems that are task-specific such as anticipatory locomotor adjustments, cognitive processing and effective goal-oriented movement strategies. Task-oriented rehabilitation differs from repetitive training because the latter method usually divides a task into its components and trains individual movements within the task, whereas task-oriented treatment focuses on acquiring a skill rather than exercising individual movements. If a technological aid (such as a robotic arm) is involved in the training, its application should allow the patient to remain actively participative. Working with a clear functional goal is a manner of goal setting that increases the effectiveness of rehabilitation (Wade, 2009). Even after finishing the training, patients are more likely to keep on achieving these functional goals and therefore to obtain better results at follow-up.

Task-oriented training has been applied throughout all stages of stroke. Winstein et al (2004) reported that less impaired stroke patients benefited more from task-oriented training than from standard post-stroke rehabilitation care in the acute stage. There are controversial data on the effect of task-oriented training when compared to Bobath treatment in acute stroke (Langhammer and Stanghelle 2000, van Vliet 2005). In both subacute and chronic stages, task-oriented upper limb training did not show significant improvement when comparing to a control group receiving standard treatment for the upper limb (Blennerhassett and Dite, 2004; Higgins et al., 2006). Despite this unsupportive evidence, it is generally agreed on that stroke rehabilitation programs should be based on task-oriented repetitive training (Oujamaa et al., 2009).

Repetitive task training (RTT)

Repetition is an important principle in motor learning which reflects the Hebbian learning rule that interneuronal connections are strengthened when simultaneously active i.e. with long term potentiation (Hebb, 1949). RTT refers to an active motor sequence performed repetitively within a single training session and aimed at achieving a clear functional goal. Each repetition is not expected to be identical to the previous one. Instead, it is suggested that implementing slight variations between repetitions is more successful (Lee et al., 1991). RTT combines functional activities with intensity of practice. Rehabilitative physiotherapy treatment favoring high-intensity repetitive task-oriented and task-specific training in all phases post stroke has proved to be essential in reducing motor impairments (Veerbeek et al., 2014). RTT potentially represents a resource-efficient component of stroke rehabilitation including delivery in a group setting or self-initiated practice in the home environment.

Duration of exercise therapy

At least 900–1200 minutes of additional therapy delivered over a 4- to 6-week period following acute stroke seems required to impact significantly on levels of impairment and/or function. A meta-analysis of 10 studies favored the use of additional exercise therapy to improve function at 6 months, using the Barthel Index as an outcome measure. Other specific outcome measures concerning upper limb function (Action Research arm Test, Motricity Index, Fugl-Meyer scale) did not yield significant summary effect sizes (SESs) (Galvin et al., 2008).

Training load of exercise therapy

The training load for the patient is determined by the total time spent on the therapeutic activity, the number of repetitions, the difficulty of the activity in terms of coordination, muscle activity type and resistance load, and the exercise intensity, i.e. the number of repetitions per time unit (Dromerick et al., 2006; Kisner and Colby, 2007). The training load should be tailored to the individual patient's capabilities and to predefined treatment goals. As an example, training goals can be an increase in muscle strength, endurance or coordination (Kerr, 1998; Mangnie, 1989). Training load needs to exceed the person's metabolic muscle capacity (overload principle) to improve muscle performance (McArdle and Jackson, 2000). When improvement of muscle strength is the goal, the training load should be such that fatigue is induced after 6 to 12 exercise repetitions. When training muscle endurance or coordination is the objective, many repetitions should be used (40–50 or more) against a submaximal load (Kisner and Colby, 2007).

Varying the type of exercises when training a specific task, improves retention of learning effects (Krakauer, 2006). Training schedules, crucial for inducing training effects, are too often determined on an empirical basis (Dromerick et al., 2006). The training environment may also influence the outcome as motor skills are shown to improve more in a natural environment than when trained out of context (Ma et al., 1999; Wu et al., 2000).

Feedback

Feedback on correct motor performance enhances motivation, while feedback on incorrect exercise performance facilitates skill improvement. Though limited evidence is available for stroke patients, positive effects of feedback have been shown on motor learning (van Vliet and Wulf, 2006). Feedback from any skill performance is acquired through task-intrinsic and task-extrinsic feedback mechanisms. Task-intrinsic feedback is provided through visual, tactile, proprioceptive and auditory input to the patient. Brain damage may impair intrinsic feedback mechanisms of stroke patients, implying that they have to rely predominantly on extrinsic or augmented feedback including verbal encouragement, charts, tones, videocamera material or computer-generated kinematic characteristics (e.g. avatar). Extrinsic feedback can be categorized as knowledge of results (KR) or knowledge of performance (KP), summary feedback (overview of results of previous trials) or average feedback (average of results of previous trials), bandwidth feedback, qualitative or quantitative feedback and can be given during or at the end of the motor task (terminal feedback) (Shumway-Cook and Woollacott, 2007; Carr and Shephard, 2003). Choosing appropriate and patient-customized feedback is complex and depends on the location and the type of the brain lesion (Boyd and Winstein, 2003; Shumway-Cook and Woollacott, 2007). Feedback that is provided simultaneously with movement execution, has not been demonstrated to support motor learning (Verschueren et al., 1997). It seems more effective to give average or summary feedback than to give feedback after each trial (Wulf et al., 1993; Winstein and Knecht, 1990), because feedback after each trial discourages variation in learning strategies (e.g. problem solving-activities), leads to feedback dependency and possibly to an attention-capacity overload (Winstein and Knecht, 1990). KR is externally presented information about outcome of skill performance or about goal achievement. KP is information about the movement characteristics that led to the performance. Though both kinds of feedback are valuable (Bilodeau et al., 1959; Siverman and Subramaniam, 1999), there is some evidence that the use of KP during repetitive movement practice results in better motor outcomes for skills learning in general (Kernodle and Carlton, 1992; Zubiaur et al., 1999) and after stroke (Cirstea et al., 2006). Van Dijk et al. (2005) performed a systematic literature search to assess the effectiveness of augmented feedback, i.e. electromyographic biofeedback, kinetic feedback, kinematic feedback or knowledge of results, and found little evidence favoring any type of augmented feedback over the other.

**References**:

Baskett, J. J., Broad, J. B., Reekie, G., Hocking, C., and Green, G. (1999). Shared responsibility for ongoing rehabilitation: a new approach to home-based therapy after stroke. doi:10.1191/026921599701532090.

Bilodeau, E. A., Bilodeau, I. M., and Schumsky, D. A. (1959). Some effects of introducing and withdrawing knowledge of results early and late in practice. *J. Exp. Psychol.* 58, 142–144. doi:10.1037/h0040262.

Blennerhassett, J., and Dite, W. (2004). Additional task-related practice improves mobility and upper limb function early after stroke: a randomised controlled trial. doi:15574110.

Boyd, L. A., and Winstein, C. J. (2003). Impact of explicit information on implicit motor-sequence learning following middle cerebral artery stroke.

Carr, J., and Shephard, R. (2003). *Stroke rehabilitation. Guidelines for exercise and training to optimize motor skill*. Edinburgh: Elsevier Science Limited.

Cirstea, C. M., Ptito, A., and Levin, M. F. (2006). Feedback and cognition in arm motor skill reacquisition after stroke. *Stroke* 37, 1237–1242. doi:10.1161/01.STR.0000217417.89347.63.

Dromerick, A. W., Lum, P. S., and Hidler, J. (2006). Activity-Based Therapies. *NeuroRx* 3, 428–438. doi:10.1016/j.nurx.2006.07.004.

Galvin, R., Murphy, B., Cusack, T., and Stokes, E. (2008). The Impact of Increased Duration of Exercise Therapy on Functional Recovery Following Stroke — What Is the Evidence? *Top. Stroke Rehabil.* 15, 365–377.

Hebb, D. (1949). *Organization of behavior: a neuropsychological theroy*. New York: John Wiley.

Higgins, J., Salbach, N. M., Wood-Dauphinee, S., Richards, C. L., Côté, R., and Mayo, N. E. (2006). The effect of a task-oriented intervention on arm function in people with stroke: a randomized controlled trial. *Clin. Rehabil.* 20, 296–310. doi:10.1191/0269215505cr943oa.

Kernodle, M. W., and Carlton, L. G. (1992). Information feedback and the learning multiple-degree-of-freedom activities. *J. Mot. Behav.* 24, 187–196. doi:10.1080/00222895.1992.9941614.

Kerr, K. (1998). *Exercise in Rehabilitation. In Rehabilitation of movement: theoretical basis of clinical practice*. , eds. Pitt-Brooke Juditch HR, L. Jane, and K. Kate London: W.B.

Kiresuk, T., Smith, A., and Cardillo, J. (1994). *Goal Attainment Scaling: Applicaitons, Theory, and Measurement*. Hillsdale, NewJersey: Lawrence Erlbaum Associates.

Kisner, C., and Colby, L. (2007). *Therapeutic Exercise: Foundations and Techniques*. 5th ed. Phiadelphia: F.A.Davis Company.

Krakauer, J. W., and Shadmehr, R. (2006). Consolidation of motor memory. *Trends Neurosci.* 29, 58–64. doi:10.1016/j.tins.2005.10.003.

Langhammer, B., and Stanghelle, J. K. (2000). Bobath or motor relearning programme? A comparison of two different approaches of physiotherapy in stroke rehabilitation: a randomized controlled study. doi:10.1191/0269215500cr338oa.

Langhorne, P., Bernhardt, J., and Kwakkel, G. (2011). Stroke rehabilitation. *Lancet* 377, 1693–1702. doi:10.1016/S0140-6736(11)60325-5.

Lee, T. D., Swanson, L. R., and Hall, A. L. (1991). What is repeated in a repetition? Effects of practice conditions on motor skill acquisition. *Phys. Ther.* 71, 150–156.

Ma, H., Trombly, C., and Robinson-Podolski, C. (1999). The effect of context on skill acquisition and transfer. *Am J Occup Ther* 53, 138–144.

Mangnie, R. (1989). Manufacturer’s: Thank Goodness There's No FDA in Rehabilitation. *J Orthop Sport. Phys Ther* 11, 1.

Mastos, M., Miller, K., Eliasson, A. C., and Imms, C. (2007). Goal-directed training: linking theories of treatment to clinical practice for improved functional activities in daily life. doi:10.1177/0269215506073494.

McArdle, A., and Jackson, M. J. (2000). Exercise, oxidative stress and ageing. *J. Anat.* 197 Pt 4, 539–541. doi:10.1046/j.1469-7580.2000.19740539.x Krakauer, J. W., and Shadmehr, R. (2006). Consolidation of motor memory. *Trends Neurosci.* 29, 58–64. doi:10.1016/j.tins.2005.10.003.

Oujamaa, L., Relave, I., Froger, J., Mottet, D., and Pelissier, J. Y. (2009). Rehabilitation of arm function after stroke. Literature review. *Ann. Phys. Rehabil. Med.* 52, 269–293. doi:10.1016/j.rehab.2008.10.003.

Shumway-Cook, A., and Woollacott, M. (2007). *Motor learning and recovery of function. In: Motor Control. Translating Research Into Clinical Practice.* Philadelphia: PA: Lippincott Williams & Wilkins.

Siverman, S. A., and Subramaniam, P. (1999). Feedback and practice in physical education: Interrelationships with task structures and student skill level. *J. Hum. Mov.* 36, 203–224.

Timmermans, A. A. A., Seelen, H. A. M., Willmann, R. D., Bakx, W., de Ruyter, B., Lanfermann, G., and Kingma, H. (2009). Arm and hand skills: training preferences after stroke. *Disabil. Rehabil.* 31, 1344–1352. doi:10.1080/09638280902823664.

Timmermans, A. A. A., Spooren, A., Kingma, H., and HA, S. (2010). Influence of task-oriented training content on skilled arm-hand performance in stroke: a systematic review. *Neurorehabil. Neural Repair* 24, 858–70.

van Dijk, H., Jannink, M. J. A., and Hermens, H. J. (2005). Effect of augmented feedback on motor function of the affected upper extremity in rehabilitation patients: a systematic review of randomized controlled trials. *J. Rehabil. Med.* 37, 202–211. doi:10.1080/16501970510030165.

van Peppen, R. P. S., Kwakkel, G., Wood-Dauphinee, S., Hendriks, H. J. M., Van der Wees, P. J., and Dekker, J. (2004). The impact of physical therapy on functional outcomes after stroke: what’s the evidence? *Clin. Rehabil.* 18, 833–862. doi:10.1191/0269215504cr843oa.

van Vliet, P. M., and Wulf, G. (2006). Extrinsic feedback for motor learning after stroke: what is the evidence? *disabil Rehabil* 28, 831–40.

van Vliet, P. M., Lincoln, N. B., and Foxall, A. (2005). Comparison of Bobath based and movement science based treatment for stroke: a randomised controlled trial. doi:10.1136/jnnp.2004.040436.

Veerbeek, J. M., van Wegen, E., van Peppen, R., van Der Wees, P. J., Hendriks, E., Rietberg, M., and Kwakkel, G. (2014). What is the evidence for physical therapy poststroke? A systematic review and meta-analysis. *PLoS One* 9. doi:10.1371/journal.pone.0087987.

Verschueren, S. M. P., Swinnen, S. P., Dom, R., and De Weerdt, W. (1997). Interlimb coordination in patients with Parkinson’s disease: Motor learning deficits and the importance of augmented information feedback. *Exp. Brain Res.* 113, 497–508. doi:10.1007/PL00005602.

Wade, D. T. (2009). Goal setting in rehabilitation: an overview of what, why and how. *Clin. Rehabil.* 23, 291–295. doi:10.1177/0269215509103551.

Winstein, C. J., and Knecht, H. (1990). Movement science and its relevance tophysical therapy. *Phys Ther* 70, 759–762.

Winstein, C. J., Rose, D. K., Tan, S. M., Lewthwaite, R., Chui, H. C., and Azen, S. P. (2004). A randomized controlled comparison of upper-extremity rehabilitation strategies in acute stroke: A pilot study of immediate and long-term outcomes. *Arch. Phys. Med. Rehabil.* 85, 620–628. doi:10.1016/j.apmr.2003.06.027.

Wressle, E., Eeg-Olofsson, A.-M., Marcusson, J., and Henriksson, C. (2002). Improved client participation in the rehabilitation process using a client-centred goal formulation structure. *J. Rehabil. Med.* 34, 5–11. doi:10.1080/165019702317242640.

Wu, C. Y., Trombly, C. A., Lin, K. C., and Tickle-Degnen, L. (2000). A kinematic study of contextual effects on reaching performance in persons with and without stroke: Influences of object availability. *Arch. Phys. Med. Rehabil.* 81, 95–101. doi:10.1053/apmr.2000.0810095.

Wulf, G., Schmidt, R. A., and Deubel, H. (1993). Reduced feedback frequency enhances generalized motor program learning but not parameterization learning. *J. Exp. Psychol. Learn. Mem. Cogn.* 19, 1134–1150. doi:10.1037/0278-7393.19.5.1134.

Zubiaur, M., Oña, A., and Delgado, J. (1999). Learning volleyball serves: a preliminary study of the effects of knowledge of performance and of results. *Percept. Mot. Skills* 89, 223–232. doi:10.2466/pms.1999.89.1.223.
