# Supplementary material for: Rehabilitation of Motor Function after Stroke: A Multiple Systematic Review Focused on Techniques to Stimulate Upper Extremity Recovery
Source: Front Hum Neurosci. 2016 Sep 13;10:442. doi: 10.3389/fnhum.2016.00442 (PMC5020059; doi:10.3389/fnhum.2016.00442)
Supplement: Supplementary file 2 [file Table1.docx]

*Research topic*

*Muscular synergies underlying complex upper-limb movements: from fundamental research to clinical application*

**Rehabilitation of motor function after stroke: a multiple systematic review focused on techniques to stimulate upper extremity recovery**

Samar M. Hatem^1,2,3^*, Geoffroy Saussez^2^, Margaux della Faille^2^, Vincent Prist^4^, Xue Zhang^5^, Delphine Dispa^2,6^, Yannick Bleyenheuft^2^

^1^ Physical and Rehabilitation Medicine, Brugmann University Hospital, Brussels, Belgium

^2^ Systems and Cognitive Neuroscience, Institute of Neuroscience, Université catholique de Louvain, Brussels, Belgium

^3^ Faculty of Medicine and Pharmacy, Faculty of Physical Education and Physiotherapy, Vrije Universiteit Brussel, Brussels, Belgium

^4^ Physical and Rehabilitation Medicine, Centre Hospitalier de l’Ardenne, Libramont, Belgium

^5^ Motor Control Laboratory, Movement Control and Neuroplasticity Research Group, Department of Kinesiology, KU Leuven, Leuven, Belgium

^6^ Physical Medicine and Rehabilitation, Cliniques universitaires Saint-Luc, Université catholique de Louvain, Brussels, Belgium

* Corresponding author:

Samar M. Hatem, MD, PhD

Physical and Rehabilitation Medicine

Brugmann University Hospital

B-1020 Brussels - Belgium

Tel: + 32 2 477 26 24

Email: samar.hatem@chu-brugmann.be

| **Table 1: Bobath therapy** | |  |  |  |  |  |  |  |  |  |
| --- | --- | --- | --- | --- | --- | --- | --- | --- | --- | --- |
| **Intervention** | **Dosage** | **Design** | **Control** | **Number of subjects (treatment / control)** | **Outcome measures** | **Results** | **Authors** | **Time post-stroke** | **Oxford evidence level** | **PEDro Score (/10)** |
| Bobath concept therapy |  | SR | control intervention | 1 review (4 RCTs 209 subjects) | upper limb impairment, activity restriction | This review found low quality or no sufficient evidence to support any change in current clinical practice. | Pollock et al., 2014 | miscellaneous | 1a | NA |
| Bobath approach |  | SR and Meta-analysis | placebo, control and/or usual care | 1 review (2 RCTs) and 3 RCTs | upper limb function: arm function tests (ARAT, FAT,…), impairment scales (FMA, MI,…) | Unknown effectiveness on arm [6 trials; 292 participants] and hand [3 trials; 208 participants] function. | Langhorne et al., 2009 | miscellaneous | 1a | NA |
| Meaningful task-specific training | 1h/d/4-5d wk/4wk | RCT | Bobath concept therapy + Brunnstrom movement therapy | 103 (51/52) | FMA, ARAT, GWMFT, MAL (AOU and QOM) | MTST group improved significantly on all outcomes in comparison with the Bobath concept therapy group. | Arya et al., 2012 | 4-24 wk | 1b | 8 |
| Bobath concept therapy + home training exercise programme | 1h/d/5d wk/2wk | RCT | CIMT 3h/d, 5d/wk during 2wk + restraining of the non-affected UE 90% of waking hours/2wk | 24 (11/13) | MAL-28 (AOU and QOM), WMFT, MESASP, FIM | Both groups showed significant improvements on MAL-28, WMFT, MESASP and FIM. CIMT group performed significantly better on MAL-28, AOU and QOM than Bobath. | Huseyinsinoglu et al., 2012 | 3-24 mo | 1b | 7 |
| Bobath concept therapy | 40min/d and 5d/wk for the duration of the hospital stay | RCT | Motor relearning group | 61 (28/33) | mAS, SMES, BI, NHP (10yr follow-up) | Both groups improved in function on the mAS but the motor relearning group showed significantly greater improvement than Bobath concept group on the mAS and SMES (only on arm lying, sitting and hand items). | Langhammer & Stanghelle, 2011 | ? | 1b | 7 |
| Augmented exercise therapy time as Bobath therapy or Augmented exercise therapy time as Arm BASIS training | 4wk | RCT | Absence of augmented exercise therapy time | 62 (21/21 /20 no AETT) | ARAT, FMA, AS | Results showed significant improvements on the FMA in all groups. No time x AETT/noAETT interaction was found but a significant time x type of intervention interaction was found with superior treatment effects using the Arm BASIS training than Bobath therapy. No significant changes were detected on resistance to passive motion and somatosensory function. Pain and passive joint motion deteriorated less in the arm Basis training than in the Bobath group. | Platz et al., 2005 | 3wk-6mo | 1b | 8 |
| Bobath concept therapy | median of 23min/weekday/±3wk | RCT | Movement science based PT | 120 (60/60) | RMA, mAS , 10HPT, NSA, BI, EADLS | No significant difference was found between both groups on all outcomes. | van Vliet et al., 2005 | ≤ 2 wk | 1b | 6 |
| Bobath concept therapy [10 with spasticity (Brunnstrom "stage 2-3) recovery (Brunnstrom stage 4-5)] | 40min/d/5 d wk/4wk 10 with relative" | RCT | Orthopaedic approach [11 with spasticity Brunnstrom stage 2-3; 12 with relative recovery Brunnstrom stage 4-5] | 44 (21/23) | SIAS, mAS , BBS, SIS | For patients with spasticity: Both groups improved significantly on the SIAS. There were significantly better results for the Bobath group than for the orthopaedic approach on the SIAS and the SIS. For patient with relative recovery: both groups significantly improved on the BBS and the SIS but only the Bobath group significantly improved on the mAS. Overall, Bobath treatment performed significantly better on mAS, SIS and BBS. No improvements were found on the SIAS. | Wang et al., 2005 | ? ("recent" stroke) | 1b | 6 |
| Bobath concept therapy | 40min/d/5d wk for all the hospitalization | RCT | Motor relearning group | 61 (28/33) | mAS , SMES, BI, NHP, BBS (1yr and 4yr follow-up) | No significant differences between groups. | Langhammer & Stanghelle, 2003 | ? | 1b | 4 |
| Bobath concept therapy | 40min/d/5d wk for all the hospitalization | RCT | Motor relearning group | 61 (28/33) | mAS , SMES, BI, NHP | Both groups improved on mAS and SMES at 3 months post-stroke (third evaluation) but the MRP group improved more than the Bobath group on mAS and SMES at second evaluation. Both groups showed similar and significant improvements on the BI. The MRP group performed significantly better on some BI items No differences were found for NHP. | Langhammer & Stanghelle, 2000 | ? | 1b | 7 |
| Arya, K. N., R. Verma, R. K. Garg, V. P. Sharma, M. Agarwal, and G. G. Aggarwal. "Meaningful Task-Specific Training (Mtst) for Stroke Rehabilitation: A Randomized Controlled Trial." [In eng]. *Top Stroke Rehabil* 19, no. 3 (May-Jun 2012): 193-211.  Huseyinsinoglu, B. E., A. R. Ozdincler, and Y. Krespi. "Bobath Concept Versus Constraint-Induced Movement Therapy to Improve Arm Functional Recovery in Stroke Patients: A Randomized Controlled Trial." [In eng]. *Clin Rehabil* 26, no. 8 (Aug 2012): 705-15.  Langhammer, B., and J. K. Stanghelle. "Can Physiotherapy after Stroke Based on the Bobath Concept Result in Improved Quality of Movement Compared to the Motor Relearning Programme." [In eng]. *Physiother Res Int* 16, no. 2 (Jun 2011): 69-80.  Langhammer, B., and J. K. Stanghelle. "Bobath or Motor Relearning Programme? A Comparison of Two Different Approaches of Physiotherapy in Stroke Rehabilitation: A Randomized Controlled Study." [In eng]. *Clin Rehabil* 14, no. 4 (Aug 2000): 361-9.  Langhammer, B., and J. K. Stanghelle. "Bobath or Motor Relearning Programme? A Follow-up One and Four Years Post Stroke." [In eng]. *Clin Rehabil* 17, no. 7 (Nov 2003): 731-4.  Langhorne, P., F. Coupar, and A. Pollock. "Motor Recovery after Stroke: A Systematic Review." [In eng]. *Lancet Neurol* 8, no. 8 (Aug 2009): 741-54.  Platz, T., C. Eickhof, S. van Kaick, U. Engel, C. Pinkowski, S. Kalok, and M. Pause. "Impairment-Oriented Training or Bobath Therapy for Severe Arm Paresis after Stroke: A Single-Blind, Multicentre Randomized Controlled Trial." [In eng]. *Clin Rehabil* 19, no. 7 (Oct 2005): 714-24.  Pollock, A., S. E. Farmer, M. C. Brady, P. Langhorne, G. E. Mead, J. Mehrholz, and F. van Wijck. "Interventions for Improving Upper Limb Function after Stroke." [In eng]. *Cochrane Database Syst Rev* 11 (2014): Cd010820.  van Vliet, P. M., N. B. Lincoln, and A. Foxall. "Comparison of Bobath Based and Movement Science Based Treatment for Stroke: A Randomised Controlled Trial." [In eng]. *J Neurol Neurosurg Psychiatry* 76, no. 4 (Apr 2005): 503-8.  Wang, R. Y., H. I. Chen, C. Y. Chen, and Y. R. Yang. "Efficacy of Bobath Versus Orthopaedic Approach on Impairment and Function at Different Motor Recovery Stages after Stroke: A Randomized Controlled Study." [In eng]. *Clin Rehabil* 19, no. 2 (Mar 2005): 155-64. | | | | | | | | | | |

| **Table 2: Muscle strengthening** | | | |  |  |  |  |  |  |  |
| --- | --- | --- | --- | --- | --- | --- | --- | --- | --- | --- |
| **Intervention** | **Dosage** | **Design** | **Control** | **Number of subjects (treatment / control)** | **Outcome measures** | **Results** | **Authors** | **Time post-stroke** | **Oxford evidence level** | **Pedro Score (/10)** |
| muscles strenghtenning |  | SR | control treatment | 1 review (13 trials; 517 participants) | upper limb function, grip strength, ADLs | Low-quality evidence exists regarding a beneficial effect on upper limb function [11 trials; 465 participants] and grip strength [6 trials; 306 participants]. | Pollock et al., 2014 | miscellaneous | 1a | NA |
| Strengthening interventions (PRE, MRE, ES) | 6w, 5 days/w, 45-90 min/day | SR: RCTs or almost-RCTs | conventional therapy | ? | Strength, spasticity, Activity (BBT, BI, FMA, NHPT) | Strength training superior over control treatment (meta-analysis) to increase UE strength both in acute and chronic stroke | Ada et al., 2006 | acute and chronic | 1a | 3 to 8 |
| Strengthening paretic muscles | 30-90 min/day, 2 to 5 x/week 2 to 6 weeks | SR of 52 studies of which 1 RCT and 1 CCT on strengthening | self-control or strengthening on LE | ? | Grip strength, force peak, FMA | Grip strength and force peak increased. No change at activity level (FMA). | Van Peppen et al., 2004 | 3 months - 4 years | 1a | 4 to 7 |
| Ada, L., S. Dorsch, and C. G. Canning. "Strengthening Interventions Increase Strength and Improve Activity after Stroke: A Systematic Review." [In eng]. *Aust J Physiother* 52, no. 4 (2006): 241-8.  Pollock, A., S. E. Farmer, M. C. Brady, P. Langhorne, G. E. Mead, J. Mehrholz, and F. van Wijck. "Interventions for Improving Upper Limb Function after Stroke." [In eng]. *Cochrane Database Syst Rev* 11 (2014): Cd010820.  Van Peppen, R. P., G. Kwakkel, S. Wood-Dauphinee, H. J. Hendriks, P. J. Van der Wees, and J. Dekker. "The Impact of Physical Therapy on Functional Outcomes after Stroke: What's the Evidence?" [In eng]. *Clin Rehabil* 18, no. 8 (Dec 2004): 833-62. | | | | | | | | | | |

| **Table 3: Isokinetic muscle strengthening** | | | |  |  |  |  |  |  |  |
| --- | --- | --- | --- | --- | --- | --- | --- | --- | --- | --- |
| **Intervention** | **Dosage** | **Design** | **Control** | **Number of subjects (treatment / control)** | **Outcome measures** | **Results** | **Authors** | **Time post-stroke** | **Oxford evidence level** | **Pedro Score (/10)** |
| isokinetic strengthening exercise |  | review | Conventional rehabilitation or concentric training or placebo (passive mobilization) | 6 trials of which 2 studies focusing on UE [1 case report and 1 open study] | FMA, AS, WMFT, FAS, FIM FAT, strength (grip, push-pull,..) | Increase of UE strength (shoulder and elbow muscles, grip strength, push and pull) Increase of activity level FMA but also on the WMFT, FAS, FIM [case report]. No changes were found on the FAT and the AS. | Hammami et al., 2012 | miscellaneous | 1a | NA |
| Hammami, N., F. O. Coroian, M. Julia, M. Amri, D. Mottet, C. Herisson, and I. Laffont. "Isokinetic Muscle Strengthening after Acquired Cerebral Damage: A Literature Review." *Ann Phys Rehabil Med* 55, no. 4 (May 2012): 279-91. | | | | | | | | | | |

| **Table 4: Stretching** | |  |  |  |  |  |  |  |  |  |
| --- | --- | --- | --- | --- | --- | --- | --- | --- | --- | --- |
| **Intervention** | **Dosage** | **Design** | **Control** | **Number of subjects (treatment / control)** | **Outcome measures** | **Results** | **Authors** | **Time post-stroke** | **Oxford evidence level** | **Pedro Score (/10)** |
| Stretching (passive, positioning, splinting and/or serial casting) |  | SR | no intervention or usual care or active interventions (PT, passive stretching, BT) | 5 reviews (55 trials) (1298 subjects) | UE imprairments, ADLs | No effect differences between stretching or control on upper limb spasticity, joint mobility or ADLs. Low quality evidence or no benefit of shoulder support. | Pollock et al., 2014 | miscellaneous | 1a | NA |
| Hands-on physical intervention or treatment component schedule (or other if one of them is in control group) |  | SR | Hands-on physical intervention or treatment component schedule (or other if one of them is in treated group) | 3 RCTs (86 subjects) | JMTT, FTT, ARAT, TPDT, JTTHF, VRS | Results showed limited evidence of benefit of stretching on upper-limb post-stroke rehabilitation. | Winter et al., 2011 | miscellaneous | 1a | NA |
| Arm stretch positioning + four-channel motor amplitude NMES + MD stroke rehabilitation (not standardized) | 2x45min/d/5d wk/8wk Total time= 60h of positioning + 51h of NMES/TENS | RCT | sham stretch positioning procedure + sham conventional TENS with minimal sensory sensation (on the forearm only) | 46 (23/23) | passive ROM (using a fluid-filled goniometer), ShoulderQ (presence of pain + timing and severity), LASIS, TS, FMA, shoulder subluxation palpation | No significant difference was found between groups for all tests. Significant improvements in passive ROM were found in both groups. | de Jong et al., 2013 | 2-8 wk | 1b | 8 |
| Finger flexors stretching protocol | 30/30sec stretch-relax during 20min x2/d/6d wk/3wk | RCT | no intervention | 21 (11/10) | mAS | Significant improvements were found for mAS in the stretched group and no improvements in the no intervention group | Jung et al., 2011 | ≥6 mo | 1b | 5 |
| Wrist and finger flexors stretching + usual upper limb rehabilitation (not standardized but no stretching) | 30min/d/5d wk/4wk + 5d/wk | RCT | usual upper limb rehabilitation (not standardized but no stretching) | 40 (20/20) | wrist and fingers flexors contracture (using the Harvey torque-controlled measures procedure), VAS, MAS (3 upper-limb items) | Though the stretching group tended to improve compared to the control group, no significant difference was found between groups for any test. | Horsley et al., 2007 | at beginning of the rehabilitation program (acute post-stroke) | 1b | 7 |
| de Jong, L. D., P. U. Dijkstra, J. Gerritsen, A. C. Geurts, and K. Postema. "Combined Arm Stretch Positioning and Neuromuscular Electrical Stimulation During Rehabilitation Does Not Improve Range of Motion, Shoulder Pain or Function in Patients after Stroke: A Randomised Trial." [In eng]. *J Physiother* 59, no. 4 (Dec 2013): 245-54.  Horsley, S. A., R. D. Herbert, and L. Ada. "Four Weeks of Daily Stretch Has Little or No Effect on Wrist Contracture after Stroke: A Randomised Controlled Trial." [In eng]. *Aust J Physiother* 53, no. 4 (2007): 239-45.  Jung, Y. J., J. H. Hong, H. G. Kwon, J. C. Song, C. Kim, S. Park, Y. K. Kim, S. H. Ahn, and S. H. Jang. "The Effect of a Stretching Device on Hand Spasticity in Chronic Hemiparetic Stroke Patients." [In eng]. NeuroRehabilitation 29, no. 1 (2011): 53-9. DOI: [10.3233/NRE-2011-0677](http://dx.doi.org/10.3233/NRE-2011-0677)  Pollock, A., S. E. Farmer, M. C. Brady, P. Langhorne, G. E. Mead, J. Mehrholz, and F. van Wijck. "Interventions for Improving Upper Limb Function after Stroke." [In eng]. *Cochrane Database Syst Rev* 11 (2014): Cd010820.  Winter, J., S. Hunter, J. Sim, and P. Crome. "Hands-on Therapy Interventions for Upper Limb Motor Dysfunction Following Stroke." [In eng]. Cochrane Database Syst Rev 6 (2011): Cd006609. | | | | | | | | | | |

| **Table 5 : Bilateral training** | |  |  |  | |  |  | | |  | |  | |  | |  | |  |
| --- | --- | --- | --- | --- | --- | --- | --- | --- | --- | --- | --- | --- | --- | --- | --- | --- | --- | --- |
| **Intervention** | **Dosage** | **Design** | **Control** | **Number of subjects (treatment / control)** | | **Outcome measures** | **Results** | | | **Authors** | | **Time post-stroke** | | **Oxford evidence level** | | **Pedro Score (/10)** | |  |
| Bilateral arm training |  | SR | usual care, control intervention or unilateral arm training | 2 reviews (18 trials, 549 participants) (9 trials, 452 participants) | | upper limb function, ADLs | Moderate-quality evidence of greater effect of unilateral than bilateral arm training on upper limb function [6 trials; 375 participants] and ADLs [3 trials; 146 participants] No difference was found between bilateral and unilateral arm training [4 trials; 228 participants]. Low-quality evidence was related to bilateral arm training compared with other interventions for UE function, impairment and ADLs outcomes | | | Pollock et al., 2014 | | miscellaneous | | 1a | | NA | |  |
| Unilateral and bilateral training |  | SR - Meta Analysis | usual care, control intervention or unilateral arm training | 1 meta analysis (452 participants, 9 study) | | upper limb function, upper limb impairment | Unilateral and bilateral training are similarly effective. Intervention success may depend on severity of upper limb paresis and time of intervention post-stroke. | | | van Delden et al., 2012 | | miscellaneous | | 1a | | 5 to 8 | |  |
| Bilateral therapy interventions |  | SR | usual care, control intervention or unilateral arm training | 1 SR (9 articles) | | upper limb function, upper limb impairment | There is some evidence that bilateral therapy improves function in adults with chronic stroke, however more quality RCTs are required to strengthen this evidence. | | | Latimer et al., 2010 | | >6months | | 1a | | NA | |  |
| Bilateral arm training |  | Meta Analysis | usual care, control intervention or unilateral arm training | 1 meta analysis (366 participants, 25 studies) | | upper limb function, upper limb impairment | Moderator variable analysis on bilateral training revealed two large and significant effects: BATRAC and EMG-triggered neuromuscular stimulation. Strong evidence supporting bilateral arm training with the caveat that two coupled protocols, rhythmic alternating movements and active stimulation, are most effective. | | | Cauraugh et al., 2010 | | miscellaneous | | 1a | | NA | |  |
| Simultaneous bilateral training |  | SR | usual care, control intervention  or unilateral arm training | 1 SR (421 participants, 14 studies) | | upper limb function, ADLs, upper limb impairment | Insufficient good quality evidence to make recommendations about the relative effect of simultaneous bilateral training compared to placebo, no intervention or usual care. Bilateral training may be no more (or less) effective than usual care or other upper limb interventions for performance in ADL functional movement of the upper limb or motor impairment outcomes. | | | Coupar et al., 2010 | | miscellaneous | | 1a | | NA | |  |
| Bilateral arm training |  | SR and Meta-analysis | placebo, control and/or usual care | 2 RCTs | | upper limb function: arm function tests (ARAT, FAT,…), impairment scales (FMA, MI,…) | Unknown effectiveness on arm [2 trials; 118 participants] and hand [1 trial; 106 participants] function. | | | Langhorne et al., 2009 | | miscellaneous | | 1a | | NA | |  |
| Bilateral movement training |  | SR - Meta Analysis | usual care, control intervention or unilateral arm training | 1 meta analysis (11 studies) | | FM, BBT, kinematic performance | Bilateral movement training was beneficial for improving motor recovery post-stroke. Bilateral movements alone or in combination with auxiliary sensory feedback are effective stroke rehabilitation protocols. | | | Stewart et al., 2006 | | miscellaneous sub-acute/ chronic | | 1a | | NA | |  |
| Strength training | 12 sessions/6weeks  (A) TOT_ST (B) TOT | RCT | 12 sessions/6weeks (C) | 20 (10, 10) | | The Upper Extremity Performance Test, shoulder flexor and handgrip strength, shoulder active range of motion, muscle tone, FMA | TOT_ST demonstrated better scores relating to unilateral tasks and in the quality aspects of bilateral movements. TOT_ST had highest muscle force gain, active range of motion and FMA. | | | da Silva et al., 2015 | | 6months-5years | | 1b | | 8 | |  |
| Bilateral movement therapy | Intensive program of precision grip training with bilateral movements 1h (3xweek)/4weeks | Crossover RCT | Intensive program of precision grip training with unilateral movements1h (3xweek)/4weeks | 10 | | grip-lift force coordination, digital dexterity, manual ability, level of satisfaction | No objective improvement in the measured variables after 8 weeks of specific intensive training. Precision grip training was shown to not generate significant differences in outcome measures. | | | Dispa et al,. 2013 | | >6months | | 2b | | 7 | |  |
| Bilateral training | Supervised BAT 20mins (5 days/week)/6weeks | RCT | Control group 20mins (5 days/week)/6weeks | 106 (56, 50) | | Action Research Arm Test, 9HPT | BAT group showed significantly greater change in dexterity during the intervention phase at 0 to 6 weeks compared to control group. The effect was lost for overall recovery at 0 to 18 weeks. No correlation between ipsilesional and contralesional recovery. | | | Morris & Van Wijck 2012 | | 2-4weeks | | 1b | | 7 | |  |
| Bilateral training | Test group:  10 sessions  (3 days) | RCT | Control group:  10 sessions (3 days) | 20 (10, 10) | | 9HPT | In the test group, speed for 9HPT with paretic hand after training was faster than value at baseline. No signifcant difference in the control group. | | | Ausenda & Carnovali, 2011 | | <6months | | 1b | | 7 | |  |
| Bilateral Arm Training | Bilateral therapy 2h (5xweek)/3weeks: (A) dCIMT (B) Bilateral arm training | RCT | (C) CT 2h (5xweek)/3weeks (C) | 66 (22, 22, 22) | | WMFT, MAL | dCIMT and BAT smoother reaching trajectories in the unilateral and bilateral tasks than the CT group. BAT, not dCIMT, generated greater force at movement initiation than CT during the unilateral and bilateral tasks. dCIMT decreased WMFT time + higher functional ability scores. MAL: better performance of the affected arm than BAT and CT. | | | Wu C.Y. et al., 2011 | | >6months | | 1b | | 6 | |  |
| Bilateral arm training | BAT 2hours (5days/week)/3weeks | RCT | Control group 2hours (5days/week)/3weeks | 33 (16, 17) | | FMA, FIM, MAL | BAT group showed better temporal and spatial efficiency during unilateral and bilateral tasks and less online error correction only during bilateral task than the control group. BAT group showed significantly greater improvement in the FMA than the control group but not in the FIM and MAL. | | | Lin et al., 2010 | | 6 to 67 months | | 1b | | 6 | |  |
| Bilateral Load Training | Coupled bilateral load training 90mins/4days: (A) with a load on the unimpaired hand (B) with no load on the unimpaired hand | RCT | (C) control (no stimulation assistance or load) | 30 (10, 10, 10) | | BBT, rapid muscle onset in a reaction time task, sustained contraction task | Both coupled bilateral no load and load groups moved more blocks and demonstrated more in sustained contraction task. Faster motor reaction times across test sessions for coupled bilateral load group provided additional evidence for improved motor capabilities. | | | Cauraugh et al., 2009 | | chronic stroke (average 4.82 years) | | 2b | | 6 | |  |
| Bilateral training | bilateral training 1h (3xweek)/8weeks | RCT | unilateral training 1h (3xweek)/8weeks | 24 (12, 12) | | MAS, MSS, Muscle strenght | Both groups showed significant improvements on MSS and strength. Bilateral group significantly greater improvement on MAS. | | | Stoykov et al., 2009 | | >6months | | 1b | | 5 | |  |
| Bilateral Arm Training | 2h (5xweek)/3weeks:  (A) CIMT (B) bilateral training | RCT | (C) CT :2h (5xweek)/3weeks (C) | 60 (20, 20, 20) | | FMA, FIM, MAL, SIS | CIMT and BAT groups showed better performance in FMA. BAT group exhibited greater gains in FMA scores, than the CIT and control groups. Enhanced performance in the MAL in CIT, FIM and SIS. | | | Lin et al., 2009a | | >6months | | 1b | | 7 | |  |
| Bilateral and Unilateral Upper-Limb | Bilateral Training 20mins (5xweeks)/6weeks | RCT | Unilateral training 20mins (5xweeks)/6weeks | 106 (50, 56) | | ARAT, 9HPT Rivermead Motor Assessment upper-limb scale, Modified Barthel Index, Hospital Anxiety and Depression Scale, Nottingham Health Profile | No significant differences at short-term. Only significant between-group difference: 9HPT and ARAT which was lower for the bilateral training group. Baseline severity significantly influenced improvement in upper-limb outcomes - irrespective of the treatment group. | | | Morris et al., 2008 | | 2-4weeks | | 1b | | 8 | |  |
| Bilateral task training | arm retraining program 15/20 sessions (45mins)/6weeks | RCT | CT 15/20 sessions (45mins)/6weeks | 41 (20, 21) | | motor function, grip strength, gross and fine manual dexterity and motor coordination, ADL, IADL | Experimental and control groups improved in the study, the statistical analyses did not show any difference between the groups. | | | Desrosiers et al., 2005 | | 10days - 2months | | 2a | | 6 | |  |
| Bilateral training Upper Extremity | Experimental group:  1 session (30mins) | RCT | Control Group:  1 session (30mins) | 36 (9, 9, 9, 9) | | isometric contractions of muscles, BIT, MAS | Increases in muscle activity were seen in experimental and control groups during bilateral practices in both actions. Bilateral effect failed to generalize to subsequent trials. | | | Mudie & Matyas, 2000 | | Acute & Chronic | | 2b | | 5 | |  |
| Bilateral Arm Training + functional electrical stimulation | BAT + FES 1 session (2xweek)/3weeks | CT | BAT 1 session (2xweek)/3weeks | 23 (10, 13) | | FMA-UE, ARAT, MAL | Favorable trend towards improvement in control group (BAT with FES) existed after treatment and at follow-up. | | | Wu F.C. et al., 2011 | | miscellaneous | | 2b | | 6 | |  |
| Robot-Assisted Arm Training + tDCS anode - lesioned hemisphere cathode - nonlesioned hemisphere | (C) AT +30sessions Sham tDCS (20mins)/6weeks | RCT | (A) AT + 30 sessions anodal tDCS (20 min)/6wks (B) AT + 30 sessions cathodal tDCS (20min)/6wks | 96 (32, 32, 32) | | FMS, UL muscle strength, BI, BBT, MRC, MAS | FMS improved in all patients at 6 weeks. No between-group differences; initial versus finish FMS scores. No significant differences between groups at 3 months. | | | Hesse et al., 2011 | | 3-8 weeks | | 1b | | 6 | |  |
| Unilateral and bilateral training | 60mins therapy (3xdays/6weeks) (A)mCIMT (B)mBATRAC | RCT | DMCT 60mins therapy (3xdays/6weeks) | 60 (22, 19, 19) | | ARAT | All groups demonstrated a significant improvement on the ARAT -persisted at 6 weeks follow-up. No significant differences in scores on ARAT were found between groups post-intervention and at follow-up. | | | van Delden et al., 2013 | | 1-6 months | | 1b | | 6 | |  |
| Bilateral Arm Training | Bilateral therapy 90to105mins (5 days/week)/4weeks : (A) therapist-based (B) robot-based | RCT | (C) control 90to105mins (5 days/week)/4weeks (C) | 42 (14, 14, 14) | | FMA, MAL, SIS | Significant effects in kinematic variables, distal part of upper limb, and in some aspects of quality of life in favor of TBAT and RBAT, compared to CT. TBAT significantly better temporal efficiency and smoothness, straighter trunk motion, compared to other groups. RBAT increased shoulder flexion compared to other groups. | | | Wu et al., 2012c | | >6months | | 1b | | 8 | |  |
| Bilateral Arm Training | BATRAC: (3xweek)/6weeks | RCT | DMTE (3xweek)/6wk | 111 (55, 56) | | FM, WMFT | Improvements of UE function in both groups and 4 months later. Satisfaction was higher after BATRAC than DMTE. BATRAC led to significantly higher increase in activation in ipsilesional precentral, anterior cingulate and postcentral gyri, supplementary motor area, contralesional superior frontal gyrus. Activation change correlated with improvement in the WMFT. | | | Whitall et al., 2011 | | >6months | | 1b | | 7 | |  |
| Robot-assisted therapy | Bi-Manu-Track 90 to 105mins (5 days/week)/4weeks | RCT | Dose-matched active control therapy 90to105mins (5 days/week)/4weeks | 20 (10, 10) | | Arm activity ratio, FMA, Functional Independence Measure, MAL, ABILHAND questionnaire | Robot-assisted therapy group significantly increased motor function, hemiplegic arm activity and bilateral arm coordination compared to control group. | | | Liao et al., 2012 | | >6 months | | 1b | | 7 | |  |
| Robot-assisted therapy (RAT) + functional-based activities (A) or RAT + distributed CIMT (B) | (A)Bi-Manu-Track 90min/day + functional activities 15-20 min/day/5 days wk/2 weeks or (B) Bi-Manu-Track 90-105min/day + dCIMT 6h/day /5 days wk/ 2 weeks | RCT | Conventional occupational therapy 90-105min/day/5 days wk/4 weeks | 48 (16/16/16) | | FMA, WMFT, MAL, accelerometers | RAT+dCIMT had greater improvement on FMA total score than RAT alone or CR alone. RAT+dCIMT and RAT better on FMA distal score than CR. RAT+dCIMt improved more on WMFT-FAS. | | | Hsieh et al., 2014 | | ≥ 6 mo | | 1b | | 8 | |  |
| Robot-assisted rehabilitation | Bi-Manu-Track 90to105mins (5 days/week)/4weeks Unilateral robot-assisted training or Bilateral robot-assisted training | RCT | CT 90to105mins (5 days/week)/4weeks | 53 (18, 18, 17) | WMFT, MAL, ABILHAND Questionnaire normalized movement time, normalized movement units, arm-trunk contribution slope | | | BRT and CT groups elicited significantly larger slope values at the start of bilateral reaching than the URT group. URT led to significantly better effects on WMFT-Time than BRT Differences in arm control kinematics and performance the MAL and ABILHAND in all groups were not significant. | Wu et al., 2013b | | 6months - 5years | | 1b | | 7 | |  |  |
| Robot assisted therapy: High-intensity or Low-intensity | Bi-Manu-Track 90 to 105min/day, 5 days/wk, 4 wks: (A) HI: 750 to 1000 mvmts  (B) 375 to 500 mvmts + after training 15 to 20 min functional activities for transfer, | RCT | control neurodevelopment therapy: same intensity 90 to 105min/day, 5 days/wk, 4 wks | 54 (18hi/18li/17ctrl) | FMA, MRC, MAL, SIS | | | All groups improved. FMA improved significantly more in HI-RT than LI-RT and control. Other measures did not differ between groups. After HI-RT, patients with moderate deficits more improvement than severe or mild deficits. | Hsieh et al., 2012 | | > 6 mo | | 1b | | 7 | |  |  |
| Robot-assisted therapy | Puma 560 (using Mirror Image Movement Enabler system for bilateral training) 50min/day/3-4 days wk/4 weeks + 5min of tone normalization before and after session; Bilateral robot-based therapy- Unilateral robot-based therapy-Combined robot-based therapy | RCT | Conventional therapy + outpatient therapies if already enrolled at the time of study acceptance | 30 (9uni/5bil/ 10 comb/6ctrl) | FMA, MSS FIM, MPE, mAS | | | Combined RAT more improvement than control group in FMA and MSS, but no difference after 6 months. | Lum et al., 2006 | | 1 - 5 mo | | 1a | | 4 | |  |  |
| Ausenda, C., and M. Carnovali. "Transfer of Motor Skill Learning from the Healthy Hand to the Paretic Hand in Stroke Patients: A Randomized Controlled Trial." [In eng]. *Eur J Phys Rehabil Med* 47, no. 3 (Sep 2011): 417-25.  Cauraugh, J. H., N. Lodha, S. K. Naik, and J. J. Summers. "Bilateral Movement Training and Stroke Motor Recovery Progress: A Structured Review and Meta-Analysis." [In eng]. Hum Mov Sci 29, no. 5 (Oct 2010): 853-70.  Cauraugh, J. H., S. A. Coombes, N. Lodha, S. K. Naik, and J. J. Summers. "Upper Extremity Improvements in Chronic Stroke: Coupled Bilateral Load Training." [In eng]. Restor Neurol Neurosci 27, no. 1 (2009): 17-25. DOI:[10.3233/RNN-2009-0455](http://dx.doi.org/10.3233/RNN-2009-0455)  Coupar, F., A. Pollock, F. van Wijck, J. Morris, and P. Langhorne. "Simultaneous Bilateral Training for Improving Arm Function after Stroke." [In eng]. *Cochrane Database Syst Rev* 4 (2010): Cd006432.  da Silva, P. B., F. N. Antunes, P. Graef, F. Cechetti, and S. Pagnussat Ade. "Strength Training Associated with Task-Oriented Training to Enhance Upper-Limb Motor Function in Elderly Patients with Mild Impairment after Stroke: A Randomized Controlled Trial." [In eng]. *Am J Phys Med Rehabil* 94, no. 1 (Jan 2015): 11-9.  Desrosiers, J., D. Bourbonnais, H. Corriveau, S. Gosselin, and G. Bravo. "Effectiveness of Unilateral and Symmetrical Bilateral Task Training for Arm During the Subacute Phase after Stroke: A Randomized Controlled Trial." [In eng]. *Clin Rehabil* 19, no. 6 (Sep 2005): 581-93.  Dispa, D., T. Lejeune, and J. L. Thonnard. "The Effect of Repetitive Rhythmic Precision Grip Task-Oriented Rehabilitation in Chronic Stroke Patients: A Pilot Study." [In eng]. *Int J Rehabil Res* 36, no. 1 (Mar 2013): 81-7.  Hesse, S., A. Waldner, J. Mehrholz, C. Tomelleri, M. Pohl, and C. Werner. "Combined Transcranial Direct Current Stimulation and Robot-Assisted Arm Training in Subacute Stroke Patients: An Exploratory, Randomized Multicenter Trial." [In eng]. *Neurorehabil Neural Repair* 25, no. 9 (Nov-Dec 2011): 838-46.  Hsieh, Y. W., C. Y. Wu, K. C. Lin, G. Yao, K. Y. Wu, and Y. J. Chang. "Dose-Response Relationship of Robot-Assisted Stroke Motor Rehabilitation: The Impact of Initial Motor Status." [In eng]. *Stroke* 43, no. 10 (Oct 2012): 2729-34.  Hsieh, Y. W., K. C. Lin, Y. S. Horng, C. Y. Wu, T. C. Wu, and F. L. Ku. "Sequential Combination of Robot-Assisted Therapy and Constraint-Induced Therapy in Stroke Rehabilitation: A Randomized Controlled Trial." [In eng]. *J Neurol* 261, no. 5 (May 2014): 1037-45.  Langhorne, P., F. Coupar, and A. Pollock. "Motor Recovery after Stroke: A Systematic Review." [In eng]. *Lancet Neurol* 8, no. 8 (Aug 2009): 741-54.  Latimer, C. P., J. Keeling, B. Lin, M. Henderson, and L. A. Hale. "The Impact of Bilateral Therapy on Upper Limb Function after Chronic Stroke: A Systematic Review." [In eng]. *Disabil Rehabil* 32, no. 15 (2010): 1221-31.  Liao, W. W., C. Y. Wu, Y. W. Hsieh, K. C. Lin, and W. Y. Chang. "Effects of Robot-Assisted Upper Limb Rehabilitation on Daily Function and Real-World Arm Activity in Patients with Chronic Stroke: A Randomized Controlled Trial." [In eng]. *Clin Rehabil* 26, no. 2 (Feb 2012): 111-20.  Lin, K. C., Y. A. Chen, C. L. Chen, C. Y. Wu, and Y. F. Chang. "The Effects of Bilateral Arm Training on Motor Control and Functional Performance in Chronic Stroke: A Randomized Controlled Study." [In eng]. *Neurorehabil Neural Repair* 24, no. 1 (Jan 2010): 42-51.  Lin, K. C., Y. F. Chang, C. Y. Wu, and Y. A. Chen. "Effects of Constraint-Induced Therapy Versus Bilateral Arm Training on Motor Performance, Daily Functions, and Quality of Life in Stroke Survivors." [In eng]. *Neurorehabil Neural Repair* 23, no. 5 (Jun 2009a): 441-8.  Lum, P. S., C. G. Burgar, M. Van der Loos, P. C. Shor, M. Majmundar, and R. Yap. "Mime Robotic Device for Upper-Limb Neurorehabilitation in Subacute Stroke Subjects: A Follow-up Study." [In eng]. *J Rehabil Res Dev* 43, no. 5 (Aug-Sep 2006): 631-42.  Morris, J. H., and F. Van Wijck. "Responses of the Less Affected Arm to Bilateral Upper Limb Task Training in Early Rehabilitation after Stroke: A Randomized Controlled Trial." [In eng]. *Arch Phys Med Rehabil* 93, no. 7 (Jul 2012): 1129-37.  Morris, J. H., F. van Wijck, S. Joice, S. A. Ogston, I. Cole, and R. S. MacWalter. "A Comparison of Bilateral and Unilateral Upper-Limb Task Training in Early Poststroke Rehabilitation: A Randomized Controlled Trial." [In eng]. *Arch Phys Med Rehabil* 89, no. 7 (Jul 2008): 1237-45.  Mudie, M. H., and T. A. Matyas. "Can Simultaneous Bilateral Movement Involve the Undamaged Hemisphere in Reconstruction of Neural Networks Damaged by Stroke?" [In eng]. *Disabil Rehabil* 22, no. 1-2 (Jan 10-20 2000): 23-37.  Pollock, A., S. E. Farmer, M. C. Brady, P. Langhorne, G. E. Mead, J. Mehrholz, and F. van Wijck. "Interventions for Improving Upper Limb Function after Stroke." [In eng]. *Cochrane Database Syst Rev* 11 (2014): Cd010820.  Stewart, K. C., J. H. Cauraugh, and J. J. Summers. "Bilateral Movement Training and Stroke Rehabilitation: A Systematic Review and Meta-Analysis." [In eng]. *J Neurol Sci* 244, no. 1-2 (May 15 2006): 89-95.  Stoykov, M. E., G. N. Lewis, and D. M. Corcos. "Comparison of Bilateral and Unilateral Training for Upper Extremity Hemiparesis in Stroke." [In eng]. *Neurorehabil Neural Repair* 23, no. 9 (Nov 2009): 945-53.  van Delden, A. L., C. L. Peper, K. N. Nienhuys, N. I. Zijp, P. J. Beek, and G. Kwakkel. "Unilateral Versus Bilateral Upper Limb Training after Stroke: The Upper Limb Training after Stroke Clinical Trial." [In eng]. *Stroke* 44, no. 9 (Sep 2013): 2613-6.  van Delden, A. L., C. L. Peper, P. J. Beek, and G. Kwakkel. "Unilateral Versus Bilateral Upper Limb Exercise Therapy after Stroke: A Systematic Review." [In eng]. *J Rehabil Med* 44, no. 2 (Feb 2012): 106-17.  Whitall, J., S. M. Waller, J. D. Sorkin, L. W. Forrester, R. F. Macko, D. F. Hanley, A. P. Goldberg, and A. Luft. "Bilateral and Unilateral Arm Training Improve Motor Function through Differing Neuroplastic Mechanisms: A Single-Blinded Randomized Controlled Trial." [In eng]. Neurorehabil Neural Repair 25, no. 2 (Feb 2011): 118-29.  Wu, C. Y., C. L. Yang, L. L. Chuang, K. C. Lin, H. C. Chen, M. D. Chen, and W. C. Huang. "Effect of Therapist-Based Versus Robot-Assisted Bilateral Arm Training on Motor Control, Functional Performance, and Quality of Life after Chronic Stroke: A Clinical Trial." [In eng]. *Phys Ther* 92, no. 8 (Aug 2012c): 1006-16.  Wu, C. Y., C. L. Yang, M. D. Chen, K. C. Lin, and L. L. Wu. "Unilateral Versus Bilateral Robot-Assisted Rehabilitation on Arm-Trunk Control and Functions Post Stroke: A Randomized Controlled Trial." [In eng]. *J Neuroeng Rehabil* 10 (2013b): 35.  Wu, C. Y., L. L. Chuang, K. C. Lin, H. C. Chen, and P. K. Tsay. "Randomized Trial of Distributed Constraint-Induced Therapy Versus Bilateral Arm Training for the Rehabilitation of Upper-Limb Motor Control and Function after Stroke." [In eng]. *Neurorehabil Neural Repair* 25, no. 2 (Feb 2011): 130-9.  Wu, F. C., Y. T. Lin, T. S. Kuo, J. J. Luh, and J. S. Lai. "Clinical Effects of Combined Bilateral Arm Training with Functional Electrical Stimulation in Patients with Stroke." [In eng]. *IEEE Int Conf Rehabil Robot* 2011 (2011): 5975367. | | | | | | | | | | | | | | | | |  |  |

| **Table 6 : Forced-use** | |  |  | |  |  |  | |  |  |  |  | |  |
| --- | --- | --- | --- | --- | --- | --- | --- | --- | --- | --- | --- | --- | --- | --- |
| **Intervention** | **Dosage** | **Design** | | **Control** | **Number of subjects (treatment / control)** | **Outcome measures** | **Results** | **Authors** | | **Time post-stroke** | **Oxford evidence level** | | **Pedro Score (/10)** | |
| Standard rehabilitation + restraining of the non paretic arm with a sling | 30-45min/d/5d wk/2wk + restraining 6h/d/2wk | RCT | standard rehabilitation | | 30 (15/15) | FMA, ARAT, mAS, 16HPT, MAS, grip strength ratio (paretic vs non-paretic hand) | Both groups significantly improved in the FMA, MAS, 16HPT and the grip strength ratio. No statistical differences were found between groups. | | Hammer & Lindmark, 2009a | 1-6 mo | 1b | 7 | |  |
| Standard rehabilitation + restraining of the non paretic arm with a sling | 30-45min/d/5d wk/2wk + restraining 6h/d/2wk | RCT | standard rehabilitation | | 30 (15/15) | MAL (AOU and QOM) | No differences were found between groups (tendency in favor of the restraining group) | | Hammer & Lindmark, 2009b | 1-6 mo | 1b | 7 | |  |
| Forced-use treatment using a resting splint restraining the healthy arm | 6h/d/5d wk/2wk (+ patients encouraged to wear the splint at home) | RCT | intensive bimanual training (neurodeveloppmental techniques) | | 66 | RAP, ARAT, FMA, MAL (AOU and QOM) | No significant differences were found between groups on the RAP, FMA and MAL-QOM. Forced-used group improved significantly more than bimanual group on the ARAT and the MAL-AOU. | | van der Lee et al., 1999 | ≥ 1 yr | 1b | 6 | |  |
| Hammer, A., and B. Lindmark. "Is Forced Use of the Paretic Upper Limb Beneficial? A Randomized Pilot Study During Subacute Post-Stroke Recovery." [In eng]. *Clin Rehabil* 23, no. 5 (May 2009a): 424-33.  Hammer, A. M., and B. Lindmark. "Effects of Forced Use on Arm Function in the Subacute Phase after Stroke: A Randomized, Clinical Pilot Study." [In eng]. *Phys Ther* 89, no. 6 (Jun 2009b): 526-39.  van der Lee, J. H., R. C. Wagenaar, G. J. Lankhorst, T. W. Vogelaar, W. L. Deville, and L. M. Bouter. "Forced Use of the Upper Extremity in Chronic Stroke Patients: Results from a Single-Blind Randomized Clinical Trial." [In eng]. *Stroke* 30, no. 11 (Nov 1999): 2369-75. | | | | | | | | | | | | | |  |

| **Table 7: Constraint-induced movement therapy** | | | |  |  |  |  |  |  |  |
| --- | --- | --- | --- | --- | --- | --- | --- | --- | --- | --- |
| **Intervention** | **Dosage** | **Design** | **Control** | **Number of subjects (treatment / control)** | **Outcome measures** | **Results** | **Authors** | **Time post-stroke** | **Oxford evidence level** | **Pedro Score (/10)** |
| CIMT |  | SR | control treatment | 2 reviews (37 trials) (1293 subjects) | upper limb function, ADLs | There is moderate-quality evidence showing better effect on upper limb function with CIMT than with control (pooled data from 14 trials/477 subjects). Low quality evidence shows better improvements in ADLs with CIMT than control (methodological limitations within reviews). | Pollock et al., 2014 | miscellaneous | 1a | NA |
| CIMT | 2-7h/d for 8-28d | Meta-analysis | other intervention | 23 trials (21 RCTs and 2 CT) (906 subjects) | Arm motor function (ARAT, FMA, mAS, WMFT,...), arm motor activity (MAL, WMFT, 9HPT,…), ADLs (BI, FIM, SIS,…), participation(SIS, FAI) | CIMT showed greater improvements than other intervention in arm motor function and motor activities. | Thrane et al., 2014 | miscellaneous | 1a | ≥4 |
| CIMT | 10d - 10wk (most commonly 3wk of therapy) | SR Meta-analysis | traditional rehabilitation | 16 RCTs (572 subjects) | MAL (AOU and QOM) [11 studies], FMA [7 studies], FIM [6 studies], ARAT [5 studies], WMFT [3 studies] | A significant treatment effect was found on the MAL (AOU and QOM), ARAT and FMA tests in the CIMT group. No significant treatment effect was found on the WMFT and the FIM. | McIntyre et al., 2012 | ≥ 6 mo | 1a | ≥4 |
| HI-CIMT or LO-CIMT | ≥3h/d + restraining less affected limb for 90% of waking hours or ≤3h/d + restraining less affect limb for less than 90% of waking hours | SR | usual care (traditional UE therapies) | 5 RCTs (106 subjects) | FMA, ARAT, GPT, MAL (AOU and QOM) | FMA, ARAT, MAL (QOM and AOU) were significantly better in LO-CIMT and better but not significantly in HI-CIMT, vs usual care group. HI-CIMT group significantly better than control on GPT. | Nijland et al., 2011 | miscellaneous (acute and subacute stroke patients. ±10 first wk) | 1a | ≥6 |
| CIMT |  | SR and Meta-analysis | placebo, control and/or usual care | 22 RCTs | upper limb function: arm function tests (ARAT, FAT,…), impairment scales (FMA, MI,…) | CIMT seems to be beneficial on arm function (21 trials analyzed; 508 subjects analyzed) | Langhorne et al., 2009 | miscellaneous | 1a | NA |
| mCIMT |  | SR Meta-analysis | dose-matched control intervention | 44 trials (1397 subjects) | Upper limb function, QOL, ADLs | Significant positive effects of mCIMT for motor function, muscle tone, arm-hand activities, self-reported amount of arm-hand use in daily life, self-reported quality of arm-hand movement in daily life, and basic ADL | Kwakkel et al., 2015 | Miscellaneous | 1a | > 4 |
| mCIMT | 20 to 30 h CIMT over 2 to 10 weeks | Meta-analysis | dose-matched control intervention | 15 RCTs (432 subjects) | Upper limb motor capacity, upper limb abilities, FIM, MAL | Compared to control interventions of equal duration and dose, CIMT produced greater improvements in a variety of indicators of UL function in adult survivors of a stroke with residual movement of their upper limb. | Stevenson et al., 2012 | miscellaneous | 1a | 6 |
| (m)CIMT | 60-72h/wk/2wk or 20-56h/wk/2wk or 30h/wk/3wk or 15-30h/wk/10wk | SR Meta-analysis | control or no treatment | 30 RCTs | MAL (AOU and QOM), ARAT, WMFT, FIM, SIS, BI | Original CIMT (±6h/d/5d wk/2wk) showed better results on WMFT and MAL (AOU and QOM) than control or no treatment groups. | Peurala et al., 2012 | miscellaneous | 1a | NA |
| mCIMT | 30min-3h/d +≤6h restraining unaffected UE/d | SR Meta-analysis | traditional rehabilitation | 13 RCTs (278 subjects) (143 mCIMT/ 135 trad rehab) | FMA [6 studies], ARAT [5 studies], FIM [3 studies], BI [1 study] MAL (AOU and QOM) [6 studies], WMFT [2 studies], SIS [1 study] Kinematic analysis variables | mCIMT showed significantly better results than traditional rehabilitation on the FMA, WMFT, MAL (AOU and QOM), ARAT and SIS. Meta-analysis showed a statistical significance on the FIM between the groups with an MD of 7. No differences were found on the kinematic variables or the BI. | Shi et al., 2011 | miscellaneous | 1a | NA |
| CIMT, mCIMT or FU | 5-45h/wk/2-10wk + 5-8h restraining time/d | SR Meta-analysis | other rehabilitative techniques, OT and PT | 18 trials (RCTs and quasi-RCTs) (674 subjects) | disability (FIM, BI) [9 studies], arm motor function (ARAT, WMFT, EMF, MAS) [14 studies] | No significant effect of CIMT was found using the disability assessments. CIMT showed a moderate effect on upper limb function | Corbetta et al., 2010 | miscellaneous | 1a | NA |
| (m)CIMT |  | SR | conventional rehabilitation and/or no treatment | 9 RCTs (243 subjects) (113/130) | ARAT [8 studies], MAL (AOU and QOM) [5 studies], FMA [5 studies], WMFT [2 studies] | Data were summarized in a narrative way. All studies showed positive results in favor of (m)CIMT groups. | Bonaiuti et al., 2007 | miscellaneous | 1a | NA |
| (m)CIMT |  | SR | alternative treatment or no treatment | 14 RCTs (286 subjects) | Upper limb function, QOL, ADLs | CIMT showed greater improvements on upper limb function than alternative or no treatment group. No evidence was found on the QOL and the independence with ADLs. | Hakkennes & Keating, 2005 | miscellaneous | 1a | 3-7 |
| CIMT | 6h/d/6d wk/15d | RCT | LF-rTMS + intensive OT + self-exercise (1200 1Hz pulses on the nonlesional hemisphere primary motor area) 22 sessions/15 days (20min LF-rTMS + 60min OT + 60 min self-exercises /day) | 66 (22/44) | FMA, WMFT, FAS | Both groups showed significant improvements in all tests, but LF-rTMS + intensive OT group showed significantly better improvements than CIMT group. | Abo et al., 2014 | ≥ 12 mo | 1b | 6 |
| mCIMT or mBATRAC | 60min/d/3d wk/6wk + encouraged to perform ADLs according to their allocated treatment (mCIMT were asked to wear a mitt for 6h/d/5d wk) | RCT | Dose-matched conventional treatment | 60 (22mCIMT/ 19mBATRAC/ 19DCMT) | ARAT, MI, FMA, 9HPT, EmNSA, MAL (AOU and QOM), SIS | All groups significantly improved in all tests but no significant difference was found between groups. | van Delden et al., 2013 | 1-6 mo | 1b | 6 |
| CIMT + transfer package (to facilitate therapeutic gains to real-world activities) | 4.5h CIMT + 0.5h transfer package/d or 5h CIMT/d /5d wk/2wk | quasi-RCT | CIMT | 23 (13/10) | FMA, MAL (AOU) | CIMT + transfer group showed significant improvements on FMA and MAL (AOU). Results of the MAL (AOU) also significantly improved between post-test and 6 months follow-up. Similar improvements were found for the CIMT group but no significant improvements were observed between post-test and 6 months follow-up test. | Takebayashi et al., 2013 | ≥ 180 d | 2a | 5 |
| mCIMT (4h mitt/day/ 4 wk) + self-training program (adjusted exercises) | 4h/wk/4 wk + expected 2-3h/d/4 wk | RCT | mBAT + self-training program (adjusted exercises) | 30 (14/16) | ARAT, 9HPT , MAL | Both groups significantly improved in ARAT and MAL, but no difference was found between groups. No significant improvement was found on 9HPT for both groups. | Brunner et al., 2012 | 2-16 wk | 1b | 7 |
| CIMT | 3h/d/5d wk/2wk + CIMT patients wear a safety mitt 90% of waking hours/12 d | RCT | Bobath concept therapy 1h/d/5d wk/2wk + home training exercise program | 24 (13/11) | MAL-28 (AOU and QOM), WMFT, MESASP, FIM | Both groups showed significant improvements in MAL-28, WMFT, MESASP and FIM. CIMT group performed significantly better on MAL-28 than Bobath group (for AOU as well as QOM). | Huseyinsinoglu et al., 2012 | 3-24 mo | 1b | 7 |
| dCIMT or dCIMT-TR + other routine rehabilitation | 2h/d/5d wk/3wk | RCT | control therapy (neurodeveloppmental principles + other routine rehabilitation | 57 (19dCIMT/20 dCIMT-TR/18ctrl) | ARAT, MAL, FAI, SIS | dCIMT and dCIMT-TR performed significantly better on ARAT, MAL (QOM) and FAI, than control group. dCIMT-TR improved more on ARAT grip subscale than dCIMT and control groups. dCIMT improved more than control on MAL AOU and more than dCIMT-TR and control on SIS. | Wu et al., 2012a | ≥ 6 mo | 1b | 6 |
| dCIMT or dCIMT-TR + other routine rehabilitation | 2h/d/5d wk/3wk | RCT | control therapy (neurodeveloppmental principles + other routine rehabilitation | 45 (15/15/15) | FMA, MAL (AOU and QOM), kinematic analyses | All groups improved significantly in FMA and MAL (AOU and QOM). dCIMT-TR group significantly better than control group in FMA (distal and total score). dCIMT-TR and dCIT groups were significantly better than controls for the MAL (AOU and QOM). dCIMT-TR group performed significantly better in kinematic analyses (grasping control and trunk compensation). | Wu et al., 2012b | ≥ 6 mo | 1b | 8 |
| mCIMT | 1h/d 5d/wk for 2 wks + mitten on unaffected arm for 4h/day | RCT | Control therapy: same amount of time | 28 (9/19) | Functional tasks for 30 sec: transfer pegs to board, grasp rubber ball, eating | The mCIMT showed significantly higher scores on the three functional tasks compared to the control group. | Treger et al.,2012 | Subacute: | 1b | 8 |
| reduced-intensity mCIMT (10min of passive mobilization + 40 min mCIMT + 10min standart ADLs acivities) | 1h individual treatment session/d + 1h household activities/d /5d wk/2wk + restraining non affected UE 12h of waking h/d/5d wk/2wk (mCIMT group) | RCT | conventional therapy for UE (20min passive mobilization and stretching + 30min active exercices + 10min standart ADLs activities) | 66 (34/32) | WMFT, MAL (AOU and QOM), AS | mCIMT group showed significantly larger improvements than control group on the MAL (AOU and QOM) and the WMFT (quality of movement) after treatment and at 3 months. No difference was found on the WMFT (test performance). Both group significantly improved on the AS but mCIMT groups showed significantly larger improvements than controls at 3 months follow-up. | Smania et al., 2012 | 3-24 mo | 1b | 9 |
| mCIMT | 3h unimanual OT sessions/5d wk/4wk + restraining of the less-affected hand for 90% of waking hours | RCT | CR (45min OT sessions/5d wk/4wk) or ICR (3h OT sessions/5d wk/4wk) | 30 (10/10/10) | WMFT (FA score and MT performance) | All groups showed significant improvements on the WMFT-FA score. On the WMFT -MT performance), only the CIMT group showed significant improvements at every assessment sessions. No significant improvements were found for the CR group. Analyses showed a significant group x time interaction. CIMT group performed significantly better on WMFT (FA and MT performance) than CR group. No significant differences were found on WMFT (FA and MT performance) results between CR and ICR groups or between ICR and CIMT groups. | Wang Q. et al., 2011 | miscellaneous (acute/subacute stroke patients) | 1b | 5 |
| dCIMT | 2h/d/5d wk/3wk + 6h restraining nonaffected UE/d/3wk | RCT | BAT or control treatment (neurodevelopmental treatment, etc) | 66 (22/22/22) | WMFT, MAL (AOU and QOM), kinematic variables | dCIMT group showed significantly higher gains on the MAL (AOU and QOM) than the BAT and the control treatment groups. dCIMT group improved more than control treatment group on the WMFT. No significant difference was found between the BAT and control treatment groups on MAL or WMFT. Both dCIMT and BAT groups showed significantly larger improvements on kinematic variables than the control treatment group. | Wu et al., 2011 | ≥ 6 mo | 1b | 7 |
| early CIMT (3-9mo post-stroke) | 1.5-4.5h/d/10d + restraining non affected UE 90% of waking hours/2wk | RCT | delayed CIMT (15-21mo post-stoke) + option to receive any rehabilitative therapy except CIMT during the year before CIMT | 222 (106/116) | WMFT, MAL (AOU and QOM), SIS | Both groups significantly improved on all tests but early CIMT showed greater improvement 12 months after therapy on WMFT, MAL (AOU and QOM) and SIS. No significant difference was found between groups 24mo after enrollment on WMFT and MAL (AOU and QOM). | Wolf et al., 2010 | 3-9 mo | 1b | 4 |
| shortened CIMT + wearing a mitt | 3h/d/5d wk/2wk + mitt wearing during 80-90% of waking hours/2wk | RCT | shortened CIMT but not wearing a mitt | 24 (12/12) | SHFT, MAS, MAL (AOU and | Both groups improved on all tests and significant improvements were maintained 12mo after therapy. No significant difference was found between groups on all tests at any time. | Brogårdh & Lexell, 2010 | 1-3 mo | 1b | 6 |
| mCIMT + Botulinum Toxin type A (Dysport 200U/mL) | 2h/d/3d wk + 5h/d of restraining nonaffected UE /3 mo + 1 injection session at beginning 1000U injected per affected UE | RCT | conventional rehabilitation (neurodevelopmental techniques) 1h PT+ 1h OT/d/3d wk/3 mo + Botulinum Toxin type A (200U/mL) | 32 (16/16) | mAS, MAL, ARAT | No difference in mAS improvements between groups (similar significant improvements).BT+mCIMT group was significant better than control on MAL (AOU and QOM) and on ARAT. | Sun et al., 2010 | ≥ 1 yr | 1b | 7 |
| Home-based CIMT | Restraining nonaffected UE 6-7 h/d  + 40 min PT 3x/wk during 4 wks | RCT | Traditional rehabilitation without CIMT | 43 (27/17) able to extend their fingers | BI | CIMT group had significantly higher score on BI than control group. Result preserved till 6 months follow-up. | Azab et al., 2009 | < 6 mo | 1b | 7 |
| shortened CIMT + wearing a mitt | 3h/d/5d wk/2wk + mitt wearing during 80-90% of waking hours/2wk | RCT | shortened CIMT but not wearing a mitt | 24 (12/12) | MAS, SHFT, MAL (AOU and QOM), 2-PDT | Both groups showed similar significant improvements (no significant differences between groups) on MAS, SHFT and MAL (AOU and QOM) after therapy and at 3mo. No significant improvement was found on the 2-PDT after shortened CIMT for both groups. | Brogårdh et al., 2009 | 1-3 mo | 1b | 7 |
| LI-CIMT or HI-CIMT | 2h/d + restraining nonaffected UE 6h/d 5d wk/2wk or 3h/d + restraining nonaffected UE during 90% waking hours/d 5d wk/2wk | RCT | traditional OT therapy 1h of ADLs retraining + 1h BAT 5d wk/2wk | 52 (19LI/16HI/ 17ctrl) | ARAT, FIM, NIHSS, SIS, | All groups improved significantly on ARAT, FIM, SIS, but HI-CIMT showed significantly lower gains than LI-CIMT or traditional OT groups in ARAT and SIS. | Dromerick et al., 2009 | ≤ 28 d | 1b | 7 |
| dCIMT or BAT + all other routine interdisciplinary stroke rehabilitation | 2h/d + restraining nonaffected UE 6h/d 5d wk/3wk or 2h/d/5d wk/3wk | RCT | control intervention (training for hand function, coordination, balance, movements of the affected, non-affected and both UL) + all other routine interdisciplinary stroke rehabilitation | 60 (20/20/20) | FMA, FIM, MAL (AOU and QOM), SIS | dCIMT and BAT groups significantly better than control on the FMA. dCIT group performed significantly better than control and BAT on the MAL (AOU and QOM), the SIS, and the locomotion subtest of the FIM. BAT group improved significantly more than control in the proximal part of the FMA. | Lin et al., 2009b | ≥ 6 mo | 1b | 8 |
| CIMT + all other routine interdisciplinary stroke rehabilitation | 2h/d + restraining nonaffected UE 5h/d 5d wk/3wk | RCT | conventional intervention with same duration of hand restraining + all other routine interdisciplinary stroke rehabilitation | 32 (16/16) | FMA, FIM, MAL, NEADL, SIS | CIMT group significantly better on the FMA, FIM, NEADL mobility domain and SIS than the control conventional intervention group. No difference was found between the MAL improvements of both groups. | Lin et al., 2009a | 6-40 mo | 1b | 7 |
| CIMT (functional training of the affected limb during the 2h sessions) | 2h/d + restraining nonaffected UE 3h/d 5d wk/3wk | RCT | control traditional intervention (neurodevelopmental techniques, weight bearing, fine motor dexterity during the 2h sessions) | 22 (12/10) | FMA, FIM, MAL, NEADL | Significant improvements were found in the CIMT vs the control group on the FMA and FIM tests. No difference was found for the MAL and NEADL total scores. | Lin et al., 2008 | ? | 1b | 7 |
| mCIMT | 4h/d/5d wk + restraining of the nonaffected UE during 90% of waking hours/7d wk /2wk | RCT | conventional rehabilitation (OT and PT using a combination of neurodevelopmental techniques) | 48 (28/20) | MAL (AOU and QOM), ARAT, mBI, 9HPT, | mCIMT group showed significantly higher improvements than control group on the MAL (AOU and MAL), ARAT and 9HPT. No significant differences were observed on the mBI. | Myint et al., 2008 | 2-16 wk | 1b | 7 |
| mCIT or Rehabilitation program | 30min/day/3d wk/10wk + restraining nonaffected UE 5h/d or 30min/day/3d wk/10wk | RCT | no treatment | 35 (13mCIT/12rehab 10notreat) | ARAT, FMA, MAL (AOU and QOM) | Significant treatment effects were found on the MAL (both AOU and QOM) and the ARAT with significant higher performance for the mCIT group. No significant treatment effect was found on the FMA. | Page et al., 2008 | ≥ 12 mo | 1b | 6 |
| CIMT | 1 CIMT session/d/5d wk/2wk + restraining of the nonaffected UE 90% of waking hours/ 2wk | RCT | usual and customary care | 30 (17/13) | WMFT, TMS | Both groups improved similarly in time- and force-based measures of the WMFT at 4 mo follow-up. No difference was found for the weight lifted, but a significant difference was found on grip strength in favor of CIMT group. | Sawaki et al., 2008 | 3-9 mo | 1b | 5 |
| CIMT | 6h/d/5d wk/2wk + restraining nonaffected UE 90% of waking hours | RCT | traditional rehabilitation | 30 (18/12) | WMFT, MAL (AOU and QOM), FIM SIS | CIMT group showed significantly larger improvements on WMFT at post-treatment evaluation than control group. Both groups showed similar significant improvements on all tests at 6mo follow-up. | Dahl et al., 2008 | ≥ 2 wk | 1b | 7 |
| mCIMT + all other routine interdisciplinary stroke rehabilitation | 2h/d/5d wk/3wk + 6h/d of restraining nonaffected UE/5d wk/3wk | RCT | traditional rehabilitation | 30 (15/15) | MAL (AOU and QOM), FIM, kinematic variables | mCIMT group significantly more improved than the traditional rehabilitation group on the MAL (on AOU and on QOM) and on the FIM. mCIMT group also significantly more improved than controls on kinematic variables (greater spatial and temporal movement efficiency). | Wu et al., 2007c | 12-36 mo | 1b | 7 |
| CIMT | 3h/d/6d wk/14-15d + restraining of the nonaffected UE 90% of waking hours | RCT | intensive traditional therapy | 23 (10/13) | FMA, GPT, MAL (AOU and QOM), | Both groups showed similar significant improvements on FMA (no significant difference but a trend in favor of CIMT). No significant differences were found on the GPT and the MAL-AOU. CIMT group improved significantly more than control group on the MAL-QOM. | Boake et al., 2007 | ≤ 14 d | 1b | 5 |
| mCIMT | 2h/d/5d wk + restraining nonaffected UE 6h/d/7d wk /3 wk | RCT | traditional rehabilitation (strengthening, balance, fine motor dexterity training, functional task practice, stretching) 2h/d/5d wk/3wk | 32 (17/15) | MAL (AOU and QOM), FIM | CIMT group showed significantly larger improvements than traditional rehabilitation on MAL (AOU and QOM) and FIM. | Lin et al., 2007 | ≥ 12 mo | 1b | 7 |
| CIMT + routine interdisciplinary stroke rehabilitation | 2h/d/5d wk/3wk + restraining nonaffected UE 6h/d/7d wk/3wk + 1.5h/d/5d wk/3wk | RCT | traditional intervention (neurodevelopmental therapy, stretching, weight bearing, fine motor dexterity training) + routine interdisciplinary stroke rehabilitation | 47 (24/23) | FMA, MAL (AOU and QOM) | Significantly larger improvements were found in the CIMT group vs the traditional intervention group on the FMA and the MAL (AOU and QOM). | Wu et al., 2007a | miscellaneous | 1b | 6 |
| mCIMT + routine interdisciplinary stroke rehabilitation | 2h/d/5d wk/3wk + restraining nonaffected UE 6h/d/7d wk/3wk | RCT | traditional intervention (neurodevelopmental therapy, stretching, weight bearing, fine motor dexterity training) + routine interdisciplinary stroke rehabilitation | 26 (13/13) | FMA, FIM, MAL (AOU and QOM), SIS | mCIMT group showed significantly larger improvements than control group on all tests. | Wu et al., 2007b | miscellaneous | 1b | 6 |
| shortened CIMT | 1h in-clinic CIMT/d + 5h unsupervised home practice/day | RCT | traditional CIMT 6h in-clinic CIMT/d | 39 (19/20) | WMFT, MAL (AOU and QOM) | Even if no differences were found between groups on the WMFT, greater gains were found on the MAL (AOU and QOM) for the traditional CIMT vs shortened CIMT group. | Richards et al., 2006 | miscellaneous | 1b | 6 |
| CIMT | 6h/d/5d wk + restraining nonaffected UE for 90% of waking hours/7d wk /2wk | CT | placebo (general fitness program: strength, stamina, balance, cognitive challenging games, relaxation) 6h/d/5d wk/2wk | 41 (21/20) | MAL (AOU and QOM), AAUT, WMFT | CIMT group showed significantly larger improvements than placebo on all tests. | Taub et al., 2006 | ≥ 1 y | 2a | 4 |
| CIMT | 6h/d/5d wk/2wk | RCT | usual and customary care (no treatment, application of orthotics, home and clinic-based OT or PT) | 222 (106/116) | WMFT, MAL (AOU and QOM), SIS | CIMT group improved significantly more than the control group on the WMFT and the MAL (on the AOU and the QOM). No significant difference was found on the SIS. | Wolf et al., 2006 | 3-9 mo | 1b | 6 |
| mCIMT | 6h/d/2wk | RCT | regular rehabilitation program (PT and/or OT) | 30 (13/17) | WMFT | mCIMT group showed larger improvements in WMFT than regular rehabilitation group (significantly better for 6/15 items of the WMFT). | Yen et al., 2005 | ? | 1b | 5 |
| CIMT + routine interdisciplinary stroke rehabilitation | 2h/d/5d wk/2wk + restraining of the nonaffected UE 6h/d/2wk | RCT | standard OT treatment and a circuit -training program (bilateral functional and ROM exercises) + routine interdisciplinary stroke rehabilitation | 23 (12/11) | ARAT, BI, FIM | CIMT group improved significantly more than control group on the ARAT, but not significantly on the BI and the FIM. | Dromerick et al., 2000 | ≤ 14 d | 1b | 6 |
| Motor skill learning | 10 repetitions of the NHPT with the non-paretic hand/d/3d | RCT | no exercise | 20 (10/10) | ST (only 3 bimanual items on the 20 items), NHPT | MSL group significantly improved on both hands using the NHPT and the control group only showed significant improvements on the non-paretic hand The ST showed significant improvements only on the MSL group (1/3 item) | Ausenda & Carnovali, 2011 | ≤ 6 mo | 1b | 6 |
| Abo, M., W. Kakuda, R. Momosaki, H. Harashima, M. Kojima, S. Watanabe, T. Sato*, et al.* "Randomized, Multicenter, Comparative Study of Neuro Versus Cimt in Poststroke Patients with Upper Limb Hemiparesis: The Neuro-Verify Study." [In eng]. *Int J Stroke* 9, no. 5 (Jul 2014): 607-12.  Ausenda, C., and M. Carnovali. "Transfer of Motor Skill Learning from the Healthy Hand to the Paretic Hand in Stroke Patients: A Randomized Controlled Trial." [In eng]. *Eur J Phys Rehabil Med* 47, no. 3 (Sep 2011): 417-25.  Azab, M., M. Al-Jarrah, M. Nazzal, M. Maayah, M. Abu Sammour, and M. Jamous. “Effectiveness of Constraint-Induced Movement Therapy (CIMT) as Home-Based Therapy on Barthel Index in Patients with Chronic Stroke”. [In eng.]. *Topics in Stroke Rehabilitation* 16, no. 3 (2009):207-11.  Boake, C., E. A. Noser, T. Ro, S. Baraniuk, M. Gaber, R. Johnson, E. T. Salmeron, et al. "Constraint-Induced Movement Therapy During Early Stroke Rehabilitation." [In eng]. Neurorehabil Neural Repair 21, no. 1 (Jan-Feb 2007): 14-24.  Bonaiuti, D., L. Rebasti, and P. Sioli. "The Constraint Induced Movement Therapy: A Systematic Review of Randomised Controlled Trials on the Adult Stroke Patients." [In eng]. Eura Medicophys 43, no. 2 (Jun 2007): 139-46.  Brogardh, C., and J. Lexell. "A 1-Year Follow-up after Shortened Constraint-Induced Movement Therapy with and without Mitt Poststroke." [In eng]. *Arch Phys Med Rehabil* 91, no. 3 (Mar 2010): 460-4.  Brogardh, C., M. Vestling, and B. H. Sjolund. "Shortened Constraint-Induced Movement Therapy in Subacute Stroke - No Effect of Using a Restraint: A Randomized Controlled Study with Independent Observers." [In eng]. *J Rehabil Med* 41, no. 4 (Mar 2009): 231-6.  Brunner, I. C., J. S. Skouen, and L. I. Strand. "Is Modified Constraint-Induced Movement Therapy More Effective Than Bimanual Training in Improving Arm Motor Function in the Subacute Phase Post Stroke? A Randomized Controlled Trial." [In eng]. *Clin Rehabil* 26, no. 12 (Dec 2012): 1078-86.  Corbetta, D., V. Sirtori, L. Moja, and R. Gatti. "Constraint-Induced Movement Therapy in Stroke Patients: Systematic Review and Meta-Analysis." [In eng]. *Eur J Phys Rehabil Med* 46, no. 4 (Dec 2010): 537-44.  Dahl, A. E., T. Askim, R. Stock, E. Langorgen, S. Lydersen, and B. Indredavik. "Short- and Long-Term Outcome of Constraint-Induced Movement Therapy after Stroke: A Randomized Controlled Feasibility Trial." [In eng]. *Clin Rehabil* 22, no. 5 (May 2008): 436-47.  Dromerick A.W., Lang C.E., Birkenmeier R.L., Wagner J.M., Miller J.P., Videen T.O., Powers W.J., Wolf S.L., Edwards D.F. (2009). Very early constraint-induced movement during stroke rehabilitation (VECTORS): a single-center RCT.*Neurology.* 73(3):195–201. doi: 10.1212/WNL.0b013e3181ab2b27.  Dromerick, A. W., D. F. Edwards, and M. Hahn. "Does the Application of Constraint-Induced Movement Therapy During Acute Rehabilitation Reduce Arm Impairment after Ischemic Stroke?" [In eng]. *Stroke* 31, no. 12 (Dec 2000): 2984-8.  Hakkennes, S., and J. L. Keating. "Constraint-Induced Movement Therapy Following Stroke: A Systematic Review of Randomised Controlled Trials." [In eng]. *Aust J Physiother* 51, no. 4 (2005): 221-31.  Huseyinsinoglu, B. E., A. R. Ozdincler, and Y. Krespi. "Bobath Concept Versus Constraint-Induced Movement Therapy to Improve Arm Functional Recovery in Stroke Patients: A Randomized Controlled Trial." [In eng]. *Clin Rehabil* 26, no. 8 (Aug 2012): 705-15.  Kwakkel, G., J. M. Veerbeek, E. E. Van Wegen, and S. L. Wolf. “Constraint-induced movement therapy after stroke”. *Lancet Neurol* 14, no. 2 (Feb 2015):224-34.  Langhorne, P., F. Coupar, and A. Pollock. "Motor Recovery after Stroke: A Systematic Review." [In eng]. *Lancet Neurol* 8, no. 8 (Aug 2009): 741-54.  Lin, K. C., C. Y. Wu, and J. S. Liu. "A Randomized Controlled Trial of Constraint-Induced Movement Therapy after Stroke." [In eng]. *Acta Neurochir Suppl* 101 (2008): 61-4.  Lin, K. C., C. Y. Wu, J. S. Liu, Y. T. Chen, and C. J. Hsu. "Constraint-Induced Therapy Versus Dose-Matched Control Intervention to Improve Motor Ability, Basic/Extended Daily Functions, and Quality of Life in Stroke." [In eng]. *Neurorehabil Neural Repair* 23, no. 2 (Feb 2009b): 160-5.  Lin, K. C., C. Y. Wu, T. H. Wei, C. Y. Lee, and J. S. Liu. "Effects of Modified Constraint-Induced Movement Therapy on Reach-to-Grasp Movements and Functional Performance after Chronic Stroke: A Randomized Controlled Study." [In eng]. *Clin Rehabil* 21, no. 12 (Dec 2007): 1075-86.  Lin, K. C., Y. F. Chang, C. Y. Wu, and Y. A. Chen. "Effects of Constraint-Induced Therapy Versus Bilateral Arm Training on Motor Performance, Daily Functions, and Quality of Life in Stroke Survivors." [In eng]. *Neurorehabil Neural Repair* 23, no. 5 (Jun 2009a): 441-8.  McIntyre, A., R. Viana, S. Janzen, S. Mehta, S. Pereira, and R. Teasell. "Systematic Review and Meta-Analysis of Constraint-Induced Movement Therapy in the Hemiparetic Upper Extremity More Than Six Months Post Stroke." [In eng]. *Top Stroke Rehabil* 19, no. 6 (Nov-Dec 2012): 499-513.  Myint, J. M., G. F. Yuen, T. K. Yu, C. P. Kng, A. M. Wong, K. K. Chow, H. C. Li, and Wong Chun Por. "A Study of Constraint-Induced Movement Therapy in Subacute Stroke Patients in Hong Kong." [In eng]. *Clin Rehabil* 22, no. 2 (Feb 2008): 112-24.  Nijland, R., G. Kwakkel, J. Bakers, and E. van Wegen. "Constraint-Induced Movement Therapy for the Upper Paretic Limb in Acute or Sub-Acute Stroke: A Systematic Review." [In eng]. *Int J Stroke* 6, no. 5 (Oct 2011): 425-33.  Page, S. J., P. Levine, A. Leonard, J. P. Szaflarski, and B. M. Kissela. "Modified Constraint-Induced Therapy in Chronic Stroke: Results of a Single-Blinded Randomized Controlled Trial." [In eng]. *Phys Ther* 88, no. 3 (Mar 2008): 333-40.  Peurala, S. H., M. P. Kantanen, T. Sjogren, J. Paltamaa, M. Karhula, and A. Heinonen. "Effectiveness of Constraint-Induced Movement Therapy on Activity and Participation after Stroke: A Systematic Review and Meta-Analysis of Randomized Controlled Trials." [In eng]. *Clin Rehabil* 26, no. 3 (Mar 2012): 209-23.  Pollock, A., S. E. Farmer, M. C. Brady, P. Langhorne, G. E. Mead, J. Mehrholz, and F. van Wijck. "Interventions for Improving Upper Limb Function after Stroke." [In eng]. *Cochrane Database Syst Rev* 11 (2014): Cd010820.  Richards, L., L. J. Gonzalez Rothi, S. Davis, S. S. Wu, and S. E. Nadeau. "Limited Dose Response to Constraint-Induced Movement Therapy in Patients with Chronic Stroke." [In eng]. *Clin Rehabil* 20, no. 12 (Dec 2006): 1066-74.  Sawaki, L., A. J. Butler, X. Leng, P. A. Wassenaar, Y. M. Mohammad, S. Blanton, K. Sathian*, et al.* "Constraint-Induced Movement Therapy Results in Increased Motor Map Area in Subjects 3 to 9 Months after Stroke." [In eng]. *Neurorehabil Neural Repair* 22, no. 5 (Sep-Oct 2008): 505-13.  Shi, Y. X., J. H. Tian, K. H. Yang, and Y. Zhao. "Modified Constraint-Induced Movement Therapy Versus Traditional Rehabilitation in Patients with Upper-Extremity Dysfunction after Stroke: A Systematic Review and Meta-Analysis." [In eng]. *Arch Phys Med Rehabil* 92, no. 6 (Jun 2011): 972-82.  Smania, N., M. Gandolfi, S. Paolucci, M. Iosa, P. Ianes, S. Recchia, C. Giovanzana*, et al.* "Reduced-Intensity Modified Constraint-Induced Movement Therapy Versus Conventional Therapy for Upper Extremity Rehabilitation after Stroke: A Multicenter Trial." [In eng]. *Neurorehabil Neural Repair* 26, no. 9 (Nov-Dec 2012): 1035-45.  Stevenson, T., L. Thalman, H. Christie, and W. Poluha. "Constraint-Induced Movement Therapy Compared to Dose-Matched Interventions for Upper-Limb Dysfunction in Adult Survivors of Stroke: A Systematic Review with Meta-Analysis." [In eng]. *Physiother Can* 64, no. 4 (Fall 2012): 397-413.  Sun, S. F., C. W. Hsu, H. P. Sun, C. W. Hwang, C. L. Yang, and J. L. Wang. "Combined Botulinum Toxin Type a with Modified Constraint-Induced Movement Therapy for Chronic Stroke Patients with Upper Extremity Spasticity: A Randomized Controlled Study." [In eng]. *Neurorehabil Neural Repair* 24, no. 1 (Jan 2010): 34-41.  Takebayashi, T., T. Koyama, S. Amano, K. Hanada, M. Tabusadani, M. Hosomi, K. Marumoto, K. Takahashi, and K. Domen. "A 6-Month Follow-up after Constraint-Induced Movement Therapy with and without Transfer Package for Patients with Hemiparesis after Stroke: A Pilot Quasi-Randomized Controlled Trial." [In eng]. *Clin Rehabil* 27, no. 5 (May 2013): 418-26.  Taub, E., G. Uswatte, D. K. King, D. Morris, J. E. Crago, and A. Chatterjee. "A Placebo-Controlled Trial of Constraint-Induced Movement Therapy for Upper Extremity after Stroke." [In eng]. *Stroke* 37, no. 4 (Apr 2006): 1045-9.  Thrane, G., O. Friborg, A. Anke, and B. Indredavik. "A Meta-Analysis of Constraint-Induced Movement Therapy after Stroke." [In eng]. *J Rehabil Med* 46, no. 9 (Oct 2014): 833-42.  Treger, I., L. Aidinof, H. Lehrer, and L. Kalichman. “Modified Constraint-Induced Movement Therapy Improved Upper Limb Function in Subacute Poststroke Patients: A Small-Scale Clinical Trial”. [In sng.] *Topics in Stroke Rehabilitation* 19, no.4 (2012):287-93.  van Delden, A. L., C. L. Peper, K. N. Nienhuys, N. I. Zijp, P. J. Beek, and G. Kwakkel. "Unilateral Versus Bilateral Upper Limb Training after Stroke: The Upper Limb Training after Stroke Clinical Trial." [In eng]. *Stroke* 44, no. 9 (Sep 2013): 2613-6.  Wang, Q., J. L. Zhao, Q. X. Zhu, J. Li, and P. P. Meng. "Comparison of Conventional Therapy, Intensive Therapy and Modified Constraint-Induced Movement Therapy to Improve Upper Extremity Function after Stroke." [In eng]. *J Rehabil Med* 43, no. 7 (Jun 2011): 619-25.  Wolf, S. L., C. J. Winstein, J. P. Miller, E. Taub, G. Uswatte, D. Morris, C. Giuliani, K. E. Light, and D. Nichols-Larsen. "Effect of Constraint-Induced Movement Therapy on Upper Extremity Function 3 to 9 Months after Stroke: The Excite Randomized Clinical Trial." [In eng]. *Jama* 296, no. 17 (Nov 1 2006): 2095-104.  Wolf, S. L., P. A. Thompson, C. J. Winstein, J. P. Miller, S. R. Blanton, D. S. Nichols-Larsen, D. M. Morris*, et al.* "The Excite Stroke Trial: Comparing Early and Delayed Constraint-Induced Movement Therapy." [In eng]. *Stroke* 41, no. 10 (Oct 2010): 2309-15.  Wu, C. Y., C. L. Chen, S. F. Tang, K. C. Lin, and Y. Y. Huang. "Kinematic and Clinical Analyses of Upper-Extremity Movements after Constraint-Induced Movement Therapy in Patients with Stroke: A Randomized Controlled Trial." [In eng]. *Arch Phys Med Rehabil* 88, no. 8 (Aug 2007a): 964-70.  Wu, C. Y., C. L. Chen, W. C. Tsai, K. C. Lin, and S. H. Chou. "A Randomized Controlled Trial of Modified Constraint-Induced Movement Therapy for Elderly Stroke Survivors: Changes in Motor Impairment, Daily Functioning, and Quality of Life." [In eng]. *Arch Phys Med Rehabil* 88, no. 3 (Mar 2007b): 273-8.  Wu, C. Y., K. C. Lin, H. C. Chen, I. H. Chen, and W. H. Hong. "Effects of Modified Constraint-Induced Movement Therapy on Movement Kinematics and Daily Function in Patients with Stroke: A Kinematic Study of Motor Control Mechanisms." [In eng]. *Neurorehabil Neural Repair* 21, no. 5 (Sep-Oct 2007c): 460-6.  Wu, C. Y., L. L. Chuang, K. C. Lin, H. C. Chen, and P. K. Tsay. "Randomized Trial of Distributed Constraint-Induced Therapy Versus Bilateral Arm Training for the Rehabilitation of Upper-Limb Motor Control and Function after Stroke." [In eng]. *Neurorehabil Neural Repair* 25, no. 2 (Feb 2011): 130-9.  Wu, C. Y., Y. A. Chen, H. C. Chen, K. C. Lin, and I. L. Yeh. "Pilot Trial of Distributed Constraint-Induced Therapy with Trunk Restraint to Improve Poststroke Reach to Grasp and Trunk Kinematics." [In eng]. *Neurorehabil Neural Repair* 26, no. 3 (Mar-Apr 2012a): 247-55.  Wu, C. Y., Y. A. Chen, K. C. Lin, C. P. Chao, and Y. T. Chen. "Constraint-Induced Therapy with Trunk Restraint for Improving Functional Outcomes and Trunk-Arm Control after Stroke: A Randomized Controlled Trial." [In eng]. *Phys Ther* 92, no. 4 (Apr 2012b): 483-92.  Yen, J. G., R. Y. Wang, H. H. Chen, and C. T. Hong. "Effectiveness of Modified Constraint-Induced Movement Therapy on Upper Limb Function in Stroke Subjects." [In eng]. *Acta Neurol Taiwan* 14, no. 1 (Mar 2005): 16-20. | | | | | | | | | | |

| **Table 8: Movement observation** | | |  |  |  |  |  |  |  |  |
| --- | --- | --- | --- | --- | --- | --- | --- | --- | --- | --- |
| **Intervention** | **Dosage** | **Design** | **Control** | **Number of subjects (treatment / control)** | **Outcome measures** | **Results** | **Authors** | **Time post-stroke** | **Oxford evidence level** | **Pedro Score (/10)** |
| inpatient rehabilitation + movement observation (watch video footage showing 20 different daily routine task carried out with the upper limb), prepare to imitate the action and then try to perform the same movements (OT helps if needed) | 3h PT ( dexterity and gait training) + 2 x 15min of video watching [1 task/d] /d/5d wk/4wk | RCT | inpatient rehabilitation + "sham" action observation (static images displaying objects) and then try to perform the movements (OT helps if needed) | 102 (53/49) | FMA, FAT, BBT, MAS, FIMM | Both groups showed significant improvements on the FAT, FMA, BBT and the FIMM. No significant improvements were found on the MAS. A significant group x time interaction was found with regards to the BBT which was significantly higher in the movement observation group. No significant differences were found on the FMA FAT, and FIMM between groups. | Franceschini et al., 2012 | 30d (±7) | 1b | 6 |
| Franceschini, M., M. G. Ceravolo, M. Agosti, P. Cavallini, S. Bonassi, V. Dall'Armi, M. Massucci, F. Schifini, and P. Sale. "Clinical Relevance of Action Observation in Upper-Limb Stroke Rehabilitation: A Possible Role in Recovery of Functional Dexterity. A Randomized Clinical Trial." [In eng]. *Neurorehabil Neural Repair* 26, no. 5 (Jun 2012): 456-62. | | | | | | | | | | |

| **Table 9 : Mirror therapy** | | |  |  |  |  |  |  |  |  |
| --- | --- | --- | --- | --- | --- | --- | --- | --- | --- | --- |
| **Intervention** | **Dosage** | **Design** | **Control** | **Number of subjects (treatment / control)** | **Outcome measures** | **Results** | **Authors** | **Time post-stroke** | **Oxford evidence level** | **Pedro Score (/10)** |
| mirror therapy |  | SR | control treatment | 1 cochrane review (14 trials; 567 participants) | upper limb function and impairment outcomes [10 trials; 421 participants], ADLs outcomes [4 trials; 217 participants] | There is moderate-quality evidence of effect of mirror therapy on upper limb function and impairment and on ADLs outcomes. | Pollock et al., 2014 | miscellaneous | 1a | NA |
| mirror therapy |  | SR | other therapy; sham therapy or no therapy | 14 trials (12 RCTs/ 2 Rcross-overCT) (567 participants) | motor function [FMA, ARAT, WMFT, MAS, BS, MI, RMAS,...], ADLs [FIM, BI], pain [VAS, NRS] | Results showed evidence for the effectiveness of mirror therapy for improving motor function after stroke (more prominent effects when compared with sham intervention than with bilateral arm training). ADLs may also be improved using MT [but only 4 studies]. MT could be applied as an additional intervention (no clear conclusion if it could replace other interventions). | Thieme et al., 2012 | miscellaneous | 1a | 2-9 (median score: 6) |
| mirror therapy | MT as a long term treatment | SR | control therapy or no treatment | 21 trials (10 RCTs/ 7 patient series/ 4 single case-study) | upper limb function, pain, lower limb function, sensation | There was moderate-quality evidence that MT as an additional therapy improves recovery of arm function [6 RCTs). Low-quality evidence was found for MT on lower limb function [1 trial] and pain [2 trials]. | Rothgangel et al., 2011 | miscellaneous | 1a | NA |
| mirror therapy |  | SR | control treatment | 15 trials (only 5 focusing on stroke patients) | functional scales, motor function of the UE, | All studies showed a trend for mirror therapy to be effective in upper limb post-stroke treatment. | Ezendam et al., 2009 | miscellaneous | 1a | NA |
| multidisciplinary rehabilitation program (conventional OT, PT, ST) + mirror therapy | 6h/d/5d wk/3wk  + 1h/d/5d wk/3wk | RCT | mulitdisciplinary rehabilitation program (conventional OT, PT, ST) | 20 (10/10) | FMA (UE), BS (arm and hand), BBT, mAS | MT group showed significantly greater improvements on the FMA, BS, and BBT than the control group. No improvements were found for the mAS on both groups. | Samuelkamaleshkumar et al., 2014 | ≤ 6 mo | 1b | 6 |
| conventional physical therapy + asymmetric training program using virtual reality reflection equipment | 2x30min/d/5d wk/4wk + 30min/d/5d wk/4wk | RCT | conventional physical therapy + symmetric training program | 30 (15/15) | FMA, BBT, ROM, MAS, grip strength (Jamar dynamometer) | Significant improvements were observed in both groups on the FMA, BBT, ROM and grip strength. Significant group x time interactions were found in favor of the asymmetric training for the BBT, grip strength, FMA (only shoulder/elbow/forearm items) and the ROM (flexion-extension and radial-ulnar deviation). No improvements were found in any group for the MAS. | Lee et al., 2014 | ? | 1b | 5 |
| conventional rehabilitation + functional electrical stimulation with mirror therapy | 60min/d/5d wk/4wk + 30min/d/5d wk/4wk | RCT | conventional rehabilitation + functional electrical stimulation | 27 (14/13) | FMA, BS, MFT, BBT | Significant improvements were observed in both groups on the FMA, BS, MFT and the BBT. The MT group showed significantly greater improvements on the FMA (wrist and hand motor skills), BS and the MFT (hand function items). | Kim et al., 2014 | ≤ 6 mo | 1b | 6 |
| mirror therapy or mirror therapy + mesh-glove afferent stimulation | 1.5h/d/5d wk/4wk | RCT | control treatment (task-oriented principles) | 43 (14/14/15ctrl) | FMA, muscle tone (Myoton-3), BBT, MAL (AOU and QOM), ABILHAND | No significant group effects were found for the muscle tone, MAL (AOU and QOM) and ABIHLAND. BBT improved significantly more with the MT+MG and the control groups than with the MT group. FMA showed significantly greater improvements in the MT and MT+MG groups than the control group. | Lin et al., 2014 | ≥ 6 mo | 1b | 7 |
| multidisciplinary rehabilitation program PT mirror therapy | 2-5h/d/5d wk/3wk  + 15 min/d/5d wk/21 days |  | mulitdisciplinary rehabilitation program without mirror therapy | 60 (30/30) | Motor FIM, FAT, MSS, WFR | Both groups showed significant improvements on all outcomes but mirror therapy group showed significantly greater improvements on the WFR (activities of daily living). | Radajewska et al., 2013 | Mean: 9 wks | 1b | 4 |
| mirror therapy (60min MT + 30min task-oriented UE training) | 1.5h/d/5d wk/4wk | RCT | task-oriented upper extremity training | 33 (16/17) | FMA, MAL (AOU and QOM), ABILHAND, RNSA | MT group improved significantly more than control group on the FMA and RNSA. No significant differences were found for the ABILHAND and MAL (AOU and QOM). | Wu et al., 2013a | ≥ 6 mo | 1b | 6 |
| individual mirror therapy or group mirror therapy | 30min/d/total of 20 sessions/5wk | RCT | control intervention (with restricted view of the affected arm) | 60 (18 individual/ 21 group/21 ctrl) | FMA, ARAT, BI, SIS, MAS | Each group significantly improved on the FMA, BI, ARAT and the SIS with no statistical group x time interaction found for all tests. | Thieme et al., 2013 | ≤ 3 mo | 1b | 8 |
| conventional treatment + mirror therapy | 1h/d/5d wk + 30min/d/5d wk /4wk | RCT | conventional treatment + placebo | 26 (13/13) | ARAT, MI, FIM | All tests showed significant improvements in both groups but MT group improved significantly more than control group on all tests. | Invernizzi et al., 2013 | ≤ 4 wk | 1b | 5 |
| conventional treatment + mirror therapy | 25 min 2x/d 5d/wk during 4 wks | RCT | conventional reatment | 26 (13/13) | FMA, Brunnstrom, MFT | All test showed significant improvements in both groups but MT improved significantly more than control group on FMA, Brunnstorm and MFT. | Lee et al. 2012 | < 6 mo | 1b | 5 |
| home-based mirror therapy (bimanual exercises) | 1h/5d wk/6wk | RCT | bimanual exercises | 40 (20/20) | FMA, grip force (Jamar dynamometer), TS, ARAT, ABILHAND pain (VAS), hand-use in ADLs (using the stroke-ULAM), EQ-5D | Only the FMA showed significantly larger improvements for the MT than the control group but this significant difference disappeared at follow-up. | Michielsen et al., 2011 | ≥ 1 yr | 1b | 8 |
| standard therapy + mirror therapy | + 30min/d/5d wk/6wk | RCT | standard therapy + control therapy | 48 (24/24) | FMA, ARAT, FIM | No significant differences were found between groups on all tests. | Dohle et al., 2009 | ≤ 8 wk | 1b | 6 |
| conventional rehabilitation program + mirror therapy | 2-5h/d/5d wk + 30min/d/5dwk /4 wk | RCT | conventional rehabilitation program + sham therapy | 40 (20/20) | BS, MAS, FIM | MT group showed significantly greater improvements than control group in the FIM and the BS. No significant differences were found on the MAS. | Yavuzer et al., 2008 | ≤ 12 mo | 1b | 7 |
| Dohle, C., J. Pullen, A. Nakaten, J. Kust, C. Rietz, and H. Karbe. "Mirror Therapy Promotes Recovery from Severe Hemiparesis: A Randomized Controlled Trial." [In eng]. *Neurorehabil Neural Repair* 23, no. 3 (Mar-Apr 2009): 209-17.  Ezendam, D., R. M. Bongers, and M. J. Jannink. "Systematic Review of the Effectiveness of Mirror Therapy in Upper Extremity Function." [In eng]. *Disabil Rehabil* 31, no. 26 (2009): 2135-49.  Invernizzi, M., S. Negrini, S. Carda, L. Lanzotti, C. Cisari, and A. Baricich. "The Value of Adding Mirror Therapy for Upper Limb Motor Recovery of Subacute Stroke Patients: A Randomized Controlled Trial." [In eng]. *Eur J Phys Rehabil Med* 49, no. 3 (Jun 2013): 311-7.  Kim, H., G. Lee, and C. Song. "Effect of Functional Electrical Stimulation with Mirror Therapy on Upper Extremity Motor Function in Poststroke Patients." [In eng]. *J Stroke Cerebrovasc Dis* 23, no. 4 (Apr 2014): 655-61.  Lee, D., M. Lee, K. Lee, and C. Song. "Asymmetric Training Using Virtual Reality Reflection Equipment and the Enhancement of Upper Limb Function in Stroke Patients: A Randomized Controlled Trial." [In eng]. *J Stroke Cerebrovasc Dis* 23, no. 6 (Jul 2014): 1319-26.  Lee, M. M., H. Y. Cho, and C. H. Song. "The Mirror Therapy Program Enhances Upper-Limb Motor Recovery and Motor Function in Acute Stroke Patients." [In eng]. *Am J Phys Med Rehabil* 91, no. 8 (Aug 2012): 689-96, quiz 97-700.  Lin, K. C., P. C. Huang, Y. T. Chen, C. Y. Wu, and W. L. Huang. "Combining Afferent Stimulation and Mirror Therapy for Rehabilitating Motor Function, Motor Control, Ambulation, and Daily Functions after Stroke." [In eng]. *Neurorehabil Neural Repair* 28, no. 2 (Feb 2014): 153-62.  Michielsen, M. E., R. W. Selles, J. N. van der Geest, M. Eckhardt, G. Yavuzer, H. J. Stam, M. Smits, G. M. Ribbers, and J. B. Bussmann. "Motor Recovery and Cortical Reorganization after Mirror Therapy in Chronic Stroke Patients: A Phase Ii Randomized Controlled Trial." [In eng]. Neurorehabil Neural Repair 25, no. 3 (Mar-Apr 2011): 223-33.  Pollock, A., S. E. Farmer, M. C. Brady, P. Langhorne, G. E. Mead, J. Mehrholz, and F. van Wijck. "Interventions for Improving Upper Limb Function after Stroke." [In eng]. Cochrane Database Syst Rev 11 (2014): Cd010820.  Radajewska, A., J.A. Opara, C. Kucio, M. Blaszcyszyn, K. Mehlich, and J. Szczygiel. “The Effects of Mirror Therapy on Arm and Hand Function in Subacute Stroke in Patients”. Int J Rehabil Res 26 (2013): 268-74.  Rothgangel, A. S., S. M. Braun, A. J. Beurskens, R. J. Seitz, and D. T. Wade. "The Clinical Aspects of Mirror Therapy in Rehabilitation: A Systematic Review of the Literature." [In eng]. Int J Rehabil Res 34, no. 1 (Mar 2011): 1-13.  Samuelkamaleshkumar, S., S. Reethajanetsureka, P. Pauljebaraj, B. Benshamir, S. M. Padankatti, and J. A. David. "Mirror Therapy Enhances Motor Performance in the Paretic Upper Limb after Stroke: A Pilot Randomized Controlled Trial." [In eng]. Arch Phys Med Rehabil 95, no. 11 (Nov 2014): 2000-5.  Thieme, H., J. Mehrholz, M. Pohl, J. Behrens, and C. Dohle. "Mirror Therapy for Improving Motor Function after Stroke." [In eng]. *Cochrane Database Syst Rev* 3 (2012): Cd008449.  Thieme, H., M. Bayn, M. Wurg, C. Zange, M. Pohl, and J. Behrens. "Mirror Therapy for Patients with Severe Arm Paresis after Stroke--a Randomized Controlled Trial." [In eng]. *Clin Rehabil* 27, no. 4 (Apr 2013): 314-24.  Wu, C. Y., P. C. Huang, Y. T. Chen, K. C. Lin, and H. W. Yang. "Effects of Mirror Therapy on Motor and Sensory Recovery in Chronic Stroke: A Randomized Controlled Trial." [In eng]. *Arch Phys Med Rehabil* 94, no. 6 (Jun 2013a): 1023-30.  Yavuzer, G., R. Selles, N. Sezer, S. Sutbeyaz, J. B. Bussmann, F. Koseoglu, M. B. Atay, and H. J. Stam. "Mirror Therapy Improves Hand Function in Subacute Stroke: A Randomized Controlled Trial." [In eng]. *Arch Phys Med Rehabil* 89, no. 3 (Mar 2008): 393-8. | | | | | | | | | | |

| **Table 10 : Mental practice with motor Imagery** | | |  |  |  |  |  |  |  |  |
| --- | --- | --- | --- | --- | --- | --- | --- | --- | --- | --- |
| **Intervention** | **Dosage** | **Design** | **Control** | **Number of subjects (treatment / control)** | **Outcome measures** | **Results** | **Authors** | **Time post-stroke** | **Oxford evidence level** | **Pedro Score (/10)** |
| Motor imagery intervention (+ co-interventions) | 180-1800min of total MI time | SR and meta-analysis | no treatment or other intervention | 6 trials (5 RCTs/1 CT) | FMA, ARAT, MAL (AOU and QOM), MI, BI | No significantly larger effect was found for the motor imagery intervention on motor function of the upper limb (only a trend). | Kho et al., 2014 | miscellaneous | 1a | 5-10 |
| Motor imagery (mental practice) + conventional therapy |  | SR | conventional therapy or conventional therapy + placebo | 3 reviews (14 trials; 421 participants) (16 trials; 652 participants) (6 trials; 119 participants) | upper limb function, ADLs, upper limb impairment | Moderate-quality evidence exists regarding effect of motor imagery as an additional intervention on upper limb function and impairment. No beneficial effect was found on ADLs. | Pollock et al., 2014 | miscellaneous | 1a | NA |
| Motor imagery (alone or in addition to other rehabilitation treatment) |  | SR | placebo, control and/or usual care | 4 RCTs (74 subjects) | arm-function tests (ARAT, FAT, MAS), impairment scales (FMA, MI) | MI seems to be beneficial for arm function. MI should be used as an adjunct to conventional therapy. | Langhorne et al., 2009 | miscellaneous | 1a | NA |
| conventional therapy (PT and/ or OT) + motor imagery | 10min-1h/d /3-5d wk/3-6wk (motor imagery) | SR | conventional therapy (PT and/ or OT) | 4 RCTs | FMA, ARAT | MI group had better results on the ARAT [2 trials]. No significant differences were found on improvements of the FMA [3 trials]. | Zimmermann-Schlatter et al., 2008 | miscellaneous | 1a | NA |
| motor imagery (mental practice) + physical therapy/ rehabilitation therapy or motor imagery alone |  | SR | rehabilitation therapy or placebo or no treatment | 10 trials (4 RCTs/ 1 CT/ 2 patient series/ 3 case reports) | functional outcomes (ARAT, FMA,…) | No clearly significant outcomes but MI may be more suitable for patients still receiving PT or OT. | Braun et al., 2006 | miscellaneous | 1a | NA |
| conventional rehabilitation therapy + motor imagery | 3h/d/5d wk/4wk + 30min/d/5d wk/4wk | RCT | conventional rehabilitation therapy | 20 (10/10) | FMA (upper limb section) | The improvements on the FMA in the MI group were significantly greater than in the control group. | Sun et al., 2013 | 3-6 mo | 1b | 6 |
| standard stroke treatment + motor imagery of upper limb movements | 45min/d/3d wk/4wk + 45min/d/3d wk/4wk | RCT | standard stroke treatment + intensive non-motor mental rehearsal or standard stroke treatment | 121 (41 motor/ 39 non-motor/ 41normal care) | ARAT, grip strength (dynamometer), BI | Each group showed significant improvements on all tests. No significant differences were found between groups on any test. | Ietswaart et al., 2011 | 1-6 mo | 1b | 6 |
| usual task-specific PT and/or OT + motor imagery into therapy + outside therapy (motor imagery) | 3 times/wk/first 3 wk and 2 times/wk/last 2 wk (± 6.5h of total motor imagery) and 5 min/d of outside therapy | RCT | usual task-specific PT and/or OT + outside therapy (physical practice) | 30 (15/15) (not only stroke) | GAS, BI, RMI ARAT, NEADLS | Each groups significantly improved on all tests and no significant differences were observed between groups on any outcome measure. | Bovend'Eerdt et al., 2010 | ? | 1b | 7 |
| EEG-based MI-BCI with robotic feedback neurorehabilitation (using the MIT-Manus) | 1h/d/3d wk/4wk | RCT | robotic rehabilitation (using the MIT-Manus) | 25 (11/14) | FMA | Both groups significantly improved on the FMA and no differences between groups were reported. | Ang et al., 2010 | ? | 1b | 4 |
| physical therapy + motor imagery (mental practice) | 30min/d/2d wk/6wk + 30min of motor imagery/session | RCT | physical therapy + relaxation | 32 (16/16) | ARAT, FMA | MI group showed significantly greater changes on the ARAT and the FMA than the control group. | Page et al., 2007 | ≥ 12 mo | 1b | 5 |
| Ang, K. K., C. Guan, K. S. Chua, B. T. Ang, C. Kuah, C. Wang, K. S. Phua, Z. Y. Chin, and H. Zhang. "Clinical Study of Neurorehabilitation in Stroke Using Eeg-Based Motor Imagery Brain-Computer Interface with Robotic Feedback." [In eng]. *Conf Proc IEEE Eng Med Biol Soc* 2010 (2010): 5549-52.  Bovend'Eerdt, T. J., H. Dawes, C. Sackley, H. Izadi, and D. T. Wade. "An Integrated Motor Imagery Program to Improve Functional Task Performance in Neurorehabilitation: A Single-Blind Randomized Controlled Trial." [In eng]. *Arch Phys Med Rehabil* 91, no. 6 (Jun 2010): 939-46.  Braun, S. M., A. J. Beurskens, P. J. Borm, T. Schack, and D. T. Wade. "The Effects of Mental Practice in Stroke Rehabilitation: A Systematic Review." [In eng]. *Arch Phys Med Rehabil* 87, no. 6 (Jun 2006): 842-52.  Ietswaart, M., M. Johnston, H. C. Dijkerman, S. Joice, C. L. Scott, R. S. MacWalter, and S. J. Hamilton. "Mental Practice with Motor Imagery in Stroke Recovery: Randomized Controlled Trial of Efficacy." [In eng]. *Brain* 134, no. Pt 5 (May 2011): 1373-86.  Kho, A. Y., K. P. Liu, and R. C. Chung. "Meta-Analysis on the Effect of Mental Imagery on Motor Recovery of the Hemiplegic Upper Extremity Function." [In eng]. *Aust Occup Ther J* 61, no. 2 (Apr 2014): 38-48.  Langhorne, P., F. Coupar, and A. Pollock. "Motor Recovery after Stroke: A Systematic Review." [In eng]. *Lancet Neurol* 8, no. 8 (Aug 2009): 741-54.  Page, S. J., P. Levine, and A. Leonard. "Mental Practice in Chronic Stroke: Results of a Randomized, Placebo-Controlled Trial." [In eng]. Stroke 38, no. 4 (Apr 2007): 1293-7.  Pollock, A., S. E. Farmer, M. C. Brady, P. Langhorne, G. E. Mead, J. Mehrholz, and F. van Wijck. "Interventions for Improving Upper Limb Function after Stroke." [In eng]. *Cochrane Database Syst Rev* 11 (2014): Cd010820.  Sun, L., D. Yin, Y. Zhu, M. Fan, L. Zang, Y. Wu, J. Jia*, et al.* "Cortical Reorganization after Motor Imagery Training in Chronic Stroke Patients with Severe Motor Impairment: A Longitudinal Fmri Study." [In eng]. *Neuroradiology* 55, no. 7 (Jul 2013): 913-25.  Zimmermann-Schlatter, A., C. Schuster, M. A. Puhan, E. Siekierka, and J. Steurer. "Efficacy of Motor Imagery in Post-Stroke Rehabilitation: A Systematic Review." [In eng]. *J Neuroeng Rehabil* 5 (2008): 8. | | | | | | | | | | |

| **Table 11: Sensory electrical stimulation: High-frequency transcutaneous electrical stimulation and electroacupuncture** | | | | | | |  |  |  |  |
| --- | --- | --- | --- | --- | --- | --- | --- | --- | --- | --- |
| **Intervention** | **Dosage** | **Design** | **Control** | **Number of subjects (treatment / control)** | **Outcome measures** | **Results** | **Authors** | **Time post-stroke** | **Oxford evidence level** | **Pedro Score (/10)** |
| TENS |  | SR | usual care, control intervention sham TENS | 1 SR (15 articles, 446 participants) | upper limb function, upper limb impairment | Although the majority of studies reported significant effects on at least 1 outcome measure, effect sizes were generally small. | Laufer and Elboim-Gabyzon, 2011 | miscellaneous | 1a | 6 |
| TENS + task-related training | TENS: 100 Hz 30min (5xweek)/4weeks | RCT | Sham TENS + task-related training 30min (5xweek)/4weeks | 30 (15, 15) | FMA, MFT, BBT, mAS | Both groups showed significant improvements in FMA, MFT and BBT after intervention. TENS group showed greater improvements in FMA, MFT and BBT. Improvements in mAS only for TENS group. | Kim et al., 2013 | >6months | 1b | 7 |
| TENS + Todd-Davies exercises | TENS: 100 Hz 40 sessions/8weeks | RCT | Sham TENS + Todd-Davies exercises  40 sessions/8weeks | 60 (30, 30) | BI, AS | Statistically significant improvements in all parameters for TENS groups, but only in some items in Sham TENS group. The change in total score was significant in both groups but improvement was significantly higher in the TENS group. Spasticity was reduced in the TENS group. | Tekeoğlu et al., 1998 | 30-240days | 1b | 5 |
| Repetitive peripheral nerve sensory stimulation (RPSS) | suprasensory RPSS: 10 Hz 2h (3xweek)/4weeks | RCT | subsensory RPSS: 10 Hz 2h (3xweek)/4weeks | 22 (11, 11) | JTTHF, pinch force, FIM, corticomotor excitability | JTTHF improved significantly in the subsensory RPSS group, but not in suprasensory RPSS group at month 1. The difference between the 2 groups was reduced by months 2 to 3. | Conforto et al., 2010 | <2 months | 1b | 6 |
| Electrical somatosensory stimulation | electrical supra-threshold somatosensory: 10 Hz for 2h, one session | Crossover RCT | control condition (idle time) for 2h, one session | 12 | index finger and hand tapping movements | Somatosensory stimulation of median nerve of affected hand, but not a period of idle time, enhanced the frequency of index finger and hand tapping movements and improved the kinematics of reach-to- grasp movements performed with the affected hand, compared with baseline. There was no impact on motor performance of the unaffected hand. | Koesler et al., 2009 | >6 month | 1b | 6 |
| Electroacupuncture | Electroacupuncture: 2 Hz + strength training (2xweek)/6 weeks | Crossover RCT | strength training (2xweek)/6 weeks | 10 | FMA, wrist spasticity, active wrist extension range of motion, isometric wrist strength | After combined treatment, the quantitative spasticity level, active wrist extension range of motion and FMA changed significantly, but not for isometric wrist strength. Strength training alone gave no significant changes for any variable. | Liu et al., 2008 | >2 years | 1b | 4 |
| Electroacupuncture | Electroacupuncture: alternating 3 / 15 Hz during 8 sessions/ 1 month + conventional rehabilitation program | RCT | conventional rehabilitation program without electroacupuncture | 63 (30, 33) | FMA, FIM | FMA improvement was significantly greater for the study group than for the control group at 2 weeks, 4 weeks and 3 months. FMA scores improved significantly in upper-limb motor function for the study group. There were no statistically significant between-group differences in FIM. | Hsieh et al., 2007 | <2weeks | 1b | 4 |
| (A) electroacupuncture (B) low-frequency TENS (C) high-frequency TENS | (A) electroacupuncture : 2 Hz (B) Low-frequency TENS : 2 Hz 20 sessions/ 10 weeks | RCT | (C) high-frequency TENS: 80 Hz 20 sessions/ 10 weeks | 150 (48, 51, 51) | motor function, activities of daily living, walking ability, social activities, life satisfaction | At 3-month and 1-year follow-ups, no clinically important or statistically significant differences observed between groups for any of the outcome variables. | Johansson et al., 2001 | 5-10 days | 1b | 8 |
| Conforto, A. B., K. N. Ferreiro, C. Tomasi, R. L. dos Santos, V. L. Moreira, S. K. Marie, S. C. Baltieri, M. Scaff, and L. G. Cohen. "Effects of Somatosensory Stimulation on Motor Function after Subacute Stroke." [In eng]. *Neurorehabil Neural Repair* 24, no. 3 (Mar-Apr 2010): 263-72.  Hsieh, R. L., L. Y. Wang, and W. C. Lee. "Additional Therapeutic Effects of Electroacupuncture in Conjunction with Conventional Rehabilitation for Patients with First-Ever Ischaemic Stroke." [In eng]. *J Rehabil Med* 39, no. 3 (Apr 2007): 205-11.  Johansson B. B., E. Haker, M. von Arbin, M. Britton, G. Langstrom, A. Terent, D. Ursing, K. Asplund and Swedish Collaboration on Sensory Stimulation After Stroke. “Acupuncture and transcutaneous nerve stimulation in stroke rehabilitation: a randomized, controlled trial.” *Stroke* 32, no.3 (Mar 2001): 707-13  Kim, T. H., T. S. In, and H. Y. Cho. "Task-Related Training Combined with Transcutaneous Electrical Nerve Stimulation Promotes Upper Limb Functions in Patients with Chronic Stroke." [In eng]. *Tohoku J Exp Med* 231, no. 2 (2013): 93-100.  Koesler, I. B., M. Dafotakis, M. Ameli, G. R. Fink, and D. A. Nowak. "Electrical Somatosensory Stimulation Improves Movement Kinematics of the Affected Hand Following Stroke." [In eng]. *J Neurol Neurosurg Psychiatry* 80, no. 6 (Jun 2009): 614-9.  Laufer, Y., and M. Elboim-Gabyzon. "Does Sensory Transcutaneous Electrical Stimulation Enhance Motor Recovery Following a Stroke? A Systematic Review." [In eng]. *Neurorehabil Neural Repair* 25, no. 9 (Nov-Dec 2011): 799-809.  Liu, W., M. Mukherjee, C. Sun, H. Liu, and L. K. McPeak. "Electroacupuncture May Help Motor Recovery in Chronic Stroke Survivors: A Pilot Study." [In eng]. *J Rehabil Res Dev* 45, no. 4 (2008): 587-95.  Tekeoglu, Y., B. Adak, and T. Goksoy. "Effect of Transcutaneous Electrical Nerve Stimulation (Tens) on Barthel Activities of Daily Living (Adl) Index Score Following Stroke." [In eng]. *Clin Rehabil* 12, no. 4 (Aug 1998): 277-80. | | | | | | | | | | |

| **Table 12: Muscle electrical stimulation : Passive and electromyography-triggered neuromuscular electrical stimulation** | | | | | | |  |  |  |  |
| --- | --- | --- | --- | --- | --- | --- | --- | --- | --- | --- |
| **Intervention** | **Dosage** | **Design** | **Control** | **Number of subjects (treatment / control)** | **Outcome measures** | **Results** | **Authors** | **Time post-stroke** | **Oxford evidence level** | **Pedro Score (/10)** |
| Electrical stimulation (neuromuscular stimulation, EMG-triggered stimulation, muscle strengthening stimulation) |  | SR | cyclical electrical stimulation or other control treatment | 3 reviews (18 trials; 706 participants) (8 trials; 157 participants) (16 trials; 638 participants) | upper limb function, ADLs, upper limb impairment | Low-quality evidence exists regarding the effectiveness of electrical stimulation. There is insufficient evidence to support any change in current clinical practice. | Pollock et al., 2014 | miscellaneous | 1a | NA |
|  |  |  |  |  |  |  |  |  |  |  |
| Electrostimulation |  | SR and Meta-analysis | placebo, control and/or usual care | 1 review (10 RCTs) and 5 RCTs. [Arm function: 13 trials; 331 participants Hand function: 5 trials; 126 participants] | upper limb function: arm function tests (ARAT, FAT,…), impairment scales (FMA, MI,…) | Electrostimulation is likely to be beneficial on arm function, but effectiveness on hand function is still unknown | Langhorne et al., 2009 | miscellaneous | 1a | NA |
| EMG-NMES |  | SR (all RCTs 1998- 2007) | usual care | 157 | B&B, FMA, ARAT, reaction time | No significant differences between groups. | Meillink et al., 2008 | 8 studies, 6 in chronic, 2 in acute | 1a | 2 to 6 |
| Electrostimulation to the peripheral neuromuscular system |  | SR RCT or quasi-RCTs | no treatment or placebo electrostimulation  or conventional therapy | 24 studies (835 subjects) | Muscle function, BBT | Muscle function and BBT improved more with NMES. FMA improved more in no treatment group. Muscle function and JTTHF improved more with NMES (1 study). NMES vs conventional therapy: FMA improved more with NMES. | Pomeroy et al., 2006 | from 9,4 days to 4,3 years | 1a | N/A |
| Low frequency – TENS Follow up - 3 years | TENS: 1.7 Hz + physiotherapy  60 min(5x/wk)/3months | RCT | Physiotherapy  60 min(5x/wk)/3months | 28 (18, 10) | FMA, mAS, BI | The motor function of the paretic arm deteriorated in both treatment and control groups. Increased spasticity was seen in both groups. ADL score remained at a similar level in the low TENS group, whereas control had deteriorated during the same time period. | Sonde et al., 2000 | 6–12 months | 1b | 4 |
| Low frequency - TENS + physiotherapy | TENS: 1.7 Hz + physiotherapy  60 min(5x/wk)/3months | RCT | Physiotherapy  60 min(5x/wk)/3months | 44 (26, 18) | FMA, mAS, BI | Motor function increased significantly in the TENS group, compared to controls. The low frequency- TENS did not decrease pain or spasticity. | Sonde et al., 1998 | 5–12 months | 1b | 4 |
| NMES | 40 min/day/ 5days/4 wks  + repetitive exercise | RCT | repetitive exercise alone OR same intensity NMES alone | 27 (9/9/9) | FMA | NMES+RE improves more than NMES alone, but not than RE alone. RE alone tends to improve more than NMES alone. | Shimodozono et al., 2014 | 3-13 weeks | 1b | 7 |
| NMES | arm stretch positioning + NMES to the shoulder  45mins / 5x per week / 8 weeks | RCT | sham arm stretch positioning + sham NMES | 39 (17, 22) | passive ROM of arm, presence of pain in the hemiplegic shoulder | Multilevel regression analysis did not show significant group effects, nor time × group interactions of passive ROM of arm. The relative risk of shoulder pain in the experimental group was non-significant. | de Jong et al., 2013 | 2-8 weeks | 1b | 8 |
| NMES | NMES to the forearm 2x30 min/workday/6 weeks + standard upper limb therapy | RCT | standard upper limb therapy | 90 (45/45) | ARAT, BI, active ROM, grip strength; | Significant effects on grip strength and ROM (wrist extension). | Rosewilliam et al., 2012 | 0 - 6 weeks no active mvmt at all | 1b | 7 |
| NMES | 30 min/day/5days wk/ 3 wks  + standard treatment 30min OT/30min PT 5 days/wk | RCT | only standard treatment 30min OT/30min PT 5 days/wk | 46 (23/23) | mAS, FMA, mBI | Larger changes after 1, 3 and 6 months for intervention group on FMA and mBI but not mAS. | Lin & Yan, 2011 | <3months | 1b | 5 |
| NMES | NMES to the shoulder  1h / 3x per day /4 weeks | RCT | sham NMES | 176 (90/86) | ARAT, FAT | No differences between groups after intervention. Results favor control group at 3 months follow-up. In severely affected stroke patients, NMES may worsen motor outcome. | Church et al., 2006 | 5 days (3-7) | 1b | 8 |
| NMES | 3 x 30min / day / 8 wks  + standard rehab | RCT | standard rehab | 60 (30/30) | ARAT, isometric strength wrist extensors grip strength, 9HPT | Significant increase of strength in wrist extensors in NMES. Effect is not maintained at 32 weeks. | Powell et al., 1999 | 2 to 4 weeks | 1b | 7 |
| NMES | 15 sessions 1h/day + routine OT/PT | RCT | placebo 15 sessions 1h/day + routine OT/PT | 46 (23/23) | FMA, Brunnstrom, FIM | Larger gains in NMES group for FMA but not for FIM. | Chae et al., 1998 | < 4 weeks | 1b | 5 |
|  |  |  |  |  |  |  |  |  |  |  |
| NMES high-frequency | 40Hz / thenar muscle / 20 min 24000 pulses 4 x wk / 4 weeks |  | NMES low frequency 20Hz / thenar muscle / 40 min 24000 pulses 4 x wk / 4 weeks | 16 | Grip strength, pinch strength, MMDT | No significant differences between groups. No pre-post effect. High functioning patients only showed improvement on MMDT and strength with high frequency. | Doucet and Griffin, 2010 | > 6 months | 2b | 4 |
| Contralaterally controlled functional electrical stimulation + therapist session | 90 min therapist (15 min control task + 75 functional tasks paretic hand) 2x/wk + home-based CCFES 55 min 2x / day during 6 weeks | RCT | 90 min therapist (15 min control task + 75 functional tasks paretic hand) 2x/wk + home-based NMES 75 min 2x / day during 6 weeks | 21 with functional hand opening in response to electrical stimulation | FMA, BBT, AMAT, max finger ext angle | Larger improvement for CCFES in all assessments, till 3 months of follow-up. | Knutson et al., 2012 | > 6 months | 1b | 6 |
| Combined Afferent Stimulation + task-specific training | motor point stimulation hand  9 sessions / 3weeks  + task-specific training | RCT | sham stimulation  9 sessions / 3weeks  + task-specific training | 37 (17, 20) | changes in dexterity, grip-lift task | The stimulation group exhibited significantly greater improvements in this task than the sham-stimulation group. No significant change in corticospinal excitability in either group. | McDonnell et al., 2007 | 1-8months | 1b | 8 |
| EMG-NEMS + mirror therapy | 2 x 20min /day / 5days/wk / 4 wks in addition to OT+PT | RCT + cross-over | OT + PT (traditional) 1 h/ day each / 4 wks | 13 | FMA, ROM, WMFT, MAL | Larger gain on FMA in EMG-NMES+MT. NB: No dissociation of EMG-NMES and MT effect. | Kojima et al., 2014 | 30 to 180 days | 1b | 6 |
| EMG-NMES | 5 x 45min /wk, 3 wk during exercise program | RCT | Passive (no EMG) NEMS or sham stimulation during exercise program | 31 (11/10/10) | FMA, MAL, self-care of FIM, ROM | All outcomes improved in active NMES. Active ROM and FMA better in both NEMS groups than in sham. No difference between EMG-NMES vs passive NMES. | Boyaci et al., 2013 | chronic | 1b | 7 |
| Hybrid assistive neuromuscular dynamic stimulation (EMG-NMES) with IVES & splint, | 8h/day for 3 weeks in addition to standard therapy (1h OT/day, 5 days/wk, 3wks) | RCT | wrist splint without HANDS + same standard therapy | 24 (12/12), unable to perform finger extension | FMA, ARAT, MAL HANDS | Greater improvement in FMA and ARAT in the intervention group. | Shindo et al., 2011 | <60 days moderate to severe hand impairment | 1b | 4 |
| EMG-NMES + bilateral movements | long term 10 protocols 90min/week/4 weeks (10 times) | RCT | short term 1 protocol 90min/week/4 weeks (once) | 19 (11/7) | BBT, fractioned reaction time, sustained force production | Superior improvement in all outcome measures in the long-term intervention group | Cauraugh et al., 2011 | > 9 months | 1b | 5 |
| EMG-NMES + bilateral movements | 3 x 30min/day / 4days wk / 2wks | RCT | EMG-triggered NMES & unilateral mvmt 3*30min/day / 4days wk / 2wks control (no ES) | 25 (10/10/5) | BBT, reaction times | Greater improvement in EMG-NMES+ bilateral movements group than other groups. EMG-NMES + unilateral movements group greater improvement than control group. | Cauraugh and Kim, 2002 | 39,1 months | 1b | 4 |
| Boyaci, A., O. Topuz, H. Alkan, M. Ozgen, A. Sarsan, N. Yildiz, and F. Ardic. "Comparison of the Effectiveness of Active and Passive Neuromuscular Electrical Stimulation of Hemiplegic Upper Extremities: A Randomized, Controlled Trial." [In eng]. *Int J Rehabil Res* 36, no. 4 (Dec 2013): 315-22.  Cauraugh, J. H., and S. Kim. "Two Coupled Motor Recovery Protocols Are Better Than One: Electromyogram-Triggered Neuromuscular Stimulation and Bilateral Movements." [In eng]. Stroke 33, no. 6 (Jun 2002): 1589-94.  Cauraugh, J. H., S. K. Naik, N. Lodha, S. A. Coombes, and J. J. Summers. "Long-Term Rehabilitation for Chronic Stroke Arm Movements: A Randomized Controlled Trial." [In eng]. Clin Rehabil 25, no. 12 (Dec 2011): 1086-96.  Chae, J., F. Bethoux, T. Bohine, L. Dobos, T. Davis, and A. Friedl. "Neuromuscular Stimulation for Upper Extremity Motor and Functional Recovery in Acute Hemiplegia." [In eng]. *Stroke* 29, no. 5 (May 1998): 975-9.  Church, C., C. Price, A. D. Pandyan, S. Huntley, R. Curless, and H. Rodgers. "Randomized Controlled Trial to Evaluate the Effect of Surface Neuromuscular Electrical Stimulation to the Shoulder after Acute Stroke." [In eng]. *Stroke* 37, no. 12 (Dec 2006): 2995-3001.  de Jong, L. D., P. U. Dijkstra, J. Gerritsen, A. C. Geurts, and K. Postema. "Combined Arm Stretch Positioning and Neuromuscular Electrical Stimulation During Rehabilitation Does Not Improve Range of Motion, Shoulder Pain or Function in Patients after Stroke: A Randomised Trial." [In eng]. *J Physiother* 59, no. 4 (Dec 2013): 245-54.  Doucet, B. M., and L. Griffin. "High-Versus Low-Frequency Stimulation Effects on Fine Motor Control in Chronic Hemiplegia: A Pilot Study." [In eng]. *Top Stroke Rehabil* 20, no. 4 (Jul-Aug 2013): 299-307.  Knutson, J. S., M. Y. Harley, T. Z. Hisel, S. D. Hogan, M. M. Maloney, and J. Chae. "Contralaterally Controlled Functional Electrical Stimulation for Upper Extremity Hemiplegia: An Early-Phase Randomized Clinical Trial in Subacute Stroke Patients." [In eng]. *Neurorehabil Neural Repair* 26, no. 3 (Mar-Apr 2012): 239-46.  Kojima, K., K. Ikuno, Y. Morii, K. Tokuhisa, S. Morimoto, and K. Shomoto. "Feasibility Study of a Combined Treatment of Electromyography-Triggered Neuromuscular Stimulation and Mirror Therapy in Stroke Patients: A Randomized Crossover Trial." [In eng]. *NeuroRehabilitation* 34, no. 2 (2014): 235-44.  Langhorne, P., F. Coupar, and A. Pollock. "Motor Recovery after Stroke: A Systematic Review." [In eng]. *Lancet Neurol* 8, no. 8 (Aug 2009): 741-54.  Lin, Z., and T. Yan. "Long-Term Effectiveness of Neuromuscular Electrical Stimulation for Promoting Motor Recovery of the Upper Extremity after Stroke." [In eng]. *J Rehabil Med* 43, no. 6 (May 2011): 506-10.  McDonnell, M. N., S. L. Hillier, T. S. Miles, P. D. Thompson, and M. C. Ridding. "Influence of Combined Afferent Stimulation and Task-Specific Training Following Stroke: A Pilot Randomized Controlled Trial." [In eng]. *Neurorehabil Neural Repair* 21, no. 5 (Sep-Oct 2007): 435-43.  Meilink A., B. Hemmen, H.A. Seelen, and G. Kwakkel. “Impact of EMG-triggered neuromuscular stimulation of the wrist and finger extensors of the paretic hand after stroke: a systematic review of the literature.” *Clin Rehabil* 22, no.4 (Apr 2008):291-305.  Pollock, A., S. E. Farmer, M. C. Brady, P. Langhorne, G. E. Mead, J. Mehrholz, and F. van Wijck. "Interventions for Improving Upper Limb Function after Stroke." [In eng]. *Cochrane Database Syst Rev* 11 (2014): Cd010820.  Pomeroy, V. M., L. King, A. Pollock, A. Baily-Hallam, and P. Langhorne. "Electrostimulation for Promoting Recovery of Movement or Functional Ability after Stroke." [In eng]. *Cochrane Database Syst Rev* 2 (2006): Cd003241.  Powell, J., A. D. Pandyan, M. Granat, M. Cameron, and D. J. Stott. "Electrical Stimulation of Wrist Extensors in Poststroke Hemiplegia." [In eng]. Stroke 30, no. 7 (Jul 1999): 1384-9.  Rosewilliam, S., S. Malhotra, C. Roffe, P. Jones, and A. D. Pandyan. "Can Surface Neuromuscular Electrical Stimulation of the Wrist and Hand Combined with Routine Therapy Facilitate Recovery of Arm Function in Patients with Stroke?" [In eng]. Arch Phys Med Rehabil 93, no. 10 (Oct 2012): 1715-21. DOI: [10.1016/j.apmr.2012.05.017](http://dx.doi.org/10.1016/j.apmr.2012.05.017)  Shimodozono, M., T. Noma, S. Matsumoto, R. Miyata, S. Etoh, and K. Kawahira. "Repetitive Facilitative Exercise under Continuous Electrical Stimulation for Severe Arm Impairment after Sub-Acute Stroke: A Randomized Controlled Pilot Study." [In eng]. *Brain Inj* 28, no. 2 (2014): 203-10.  Shindo, K., T. Fujiwara, J. Hara, H. Oba, F. Hotta, T. Tsuji, K. Hase, and M. Liu. "Effectiveness of Hybrid Assistive Neuromuscular Dynamic Stimulation Therapy in Patients with Subacute Stroke: A Randomized Controlled Pilot Trial." [In eng]. *Neurorehabil Neural Repair* 25, no. 9 (Nov-Dec 2011): 830-7.  Sonde, L., C. Gip, S. E. Fernaeus, C. G. Nilsson, and M. Viitanen. "Stimulation with Low Frequency (1.7 Hz) Transcutaneous Electric Nerve Stimulation (Low-Tens) Increases Motor Function of the Post-Stroke Paretic Arm." [In eng]. *Scand J Rehabil Med* 30, no. 2 (Jun 1998): 95-9.  Sonde, L., H. Kalimo, S. E. Fernaeus, and M. Viitanen. "Low Tens Treatment on Post-Stroke Paretic Arm: A Three-Year Follow-Up." [In eng]. *Clin Rehabil* 14, no. 1 (Feb 2000): 14-9. | | | | | | | | | | |

| **Table 13 : Repetitive transcranial magnetic stimulation** | | | |  |  |  |  |  |  |  |
| --- | --- | --- | --- | --- | --- | --- | --- | --- | --- | --- |
| **Intervention** | **Dosage** | **Design** | **Control** | **Number of subjects (treatment / control)** | **Outcome measures** | **Results** | **Authors** | **Time post-stroke** | **Oxford evidence level** | **Pedro Score (/10)** |
| rTMS |  | SR | sham rTMS and/or other intervention | 1 review (19 trials; 588 participants) | upper limb function, ADLs, upper limb impairment | Low-quality evidence of the impact of rTMS on upper limb function. Insufficient evidence to support any change in current clinical practice. | Pollock et al., 2014 | miscellaneous | 1a | NA |
| rTMS |  | Meta Analysis | sham rTMS and/or other intervention | 1 meta analysis (273 participants, 8 articles) | hand function recovery, finger ability | Positive effects of rTMS on finger motor ability and hand function. Changes of neurophysiologic measurements were not significant in the included studies. | Le et al., 2014 | miscellaneous | 1a | ≥6 |
| rTMS |  | SR | sham rTMS and/or other intervention | 1 review (19 trials; 588 participants) |  | rTMS treatment was not associated with improved activities of daily living + no statistically significant effect on motor function. There was insufficient evidence to support rTMS for the treatment of stroke. | Hao et al., 2013 | miscellaneous | 1a | NA |
| rTMS |  | Meta Analysis | sham rTMS and/or other intervention | 1 meta analysis (392 participants, 18 articles) | upper limb motor function | rTMS had a positive effect on motor recovery in patients with stroke, especially for those with subcortical stroke. Low-frequency rTMS over the unaffected hemisphere was more beneficial than high-frequency rTMS over the affected hemisphere. iTBS over the affected hemisphere may be a useful intervention. | Hsu et al., 2012 | miscellaneous | 1a | NA |
| rTMS |  | SR | sham rTMS and/or other intervention | 1 review (508 participants; 37 studies) | excitability of corticospinal pathways, movement control, functional motor ability | For healthy adults, the effects of rTMS on corticospinal pathway excitability varied within each frequency. After stroke there was a trend for recovery of MEPs after 10 daily sessions of 3 Hz rTMS. | Hiscock et al., 2008 | miscellaneous | 1a | NA |
| rTMS | rTMS (A) HF-rTMS group (10 Hz rTMS on ipsilesional hemisphere) during 5days (B) LF-rTMS group (1 Hz rTMS on contralesional hemisphere) during 5days | RCT | Sham rTMS | 29 (9, 11, 9) | Grip strength, tapping frequency | HF-rTMS and LF-rTMS elicited a significant increase in grip strength and tapping frequency. There was a more significant increase in HF-rTMS compared to sham. No differences were observed between LF-rTMS and sham. | Sasaki et al., 2013 | ≤30 days | 1b | 7 |
| rTMS | 1 Hz rTMS contralesional 1500pulses/25mins/day during 2weeks | RCT | Sham rTMS | 30 (15, 15) | JTTHF, FMA, AS, mRS, Force of lateral pinch paretic hand | Significant improvement in performance JTTHF and pinch force in the active group, but not in the sham group. | Conforto et al., 2012 | 5–45 days | 1b | 8 |
| rTMS | 10 sessions of 1 Hz rTMS of AH (1000 pulses) during 10 days | RCT | Sham rTMS | 21 (11, 10) | Sequential Finger Motor Task (mT, MA) | MA was significantly improved in the intervention group. There was a significant interaction effect in the sensorimotor cortex, thalamus, and caudate. | Chang et al., 2012 | ≥3 months | 1b | 7 |
| rTMS | (A) 3Hz rTMS/750 pulses on unaffected hemisphere (B) 10Hz rTMS/750 pulses on unaffected hemisphere | RCT | (C) Sham rTMS | 48 (16, 16, 16) | Hand Grip, Shoulder abduction, Dorsiflexion of toes, Hip flexion, NIHSS, mRS | Real rTMS produced a greater improvement than sham, which was evident even at one year follow-up. Improvements were associated with changes in cortical excitability over the period of treatment. | Khedr et al., 2010 | 5–15 days | 1b | 7 |
| rTMS | 1 Hz rTMS (900pulses) 1x/day during 5days or 3 Hz rTMS (900pulses) 1x/day during 5days | RCT | Sham rTMS | 36 (12, 12, 12) | Strength of hand grip, BI, PPT, Keyboard Tapping | Both rTMS groups improved on outcome measures. 1Hz performed better than 3Hz. 1Hz reduced the excitability of the non-stroke hemisphere and excitability of the stroke hemisphere. 3Hz only increased excitability of the stroke hemisphere. | Khedr et al., 2009 | acute | 1b | 7 |
| rTMS | Bilateral rTMS: 1Hz rTMS unaffected hemisphere + 10Hz rTMS affected hemisphere | RCT | 1Hz rTMS unaffected hemisphere + Sham 10Hz rTMS affected hemisphere or Sham 1Hz rTMS unaffected hemisphere + 10Hz rTMS affected hemisphere | 30 (10, 10, 10) | Acceleration, Pinch Force, FMA | Bilateral rTMS improved acceleration of paretic hand. Effect of motor training lasted for 1 week 10 Hz. There was no effect on motor function. | Takeuchi et al., 2009 | >6 months | 2b | 7 |
| rTMS | 8 sessions of 10 Hz rTMS (20 pulses, 10Hz/2secs, 8mins/ 58secs interval) + Finger Motor Task during 7 days | Crossover CT | Sham rTMS | 15 | Finger Motor Task (mT, MA) | Real rTMS induced a significantly larger increase in MEP amplitude. Plastic change was positively associated with enhanced MA. | Kim et al., 2006 | >3 months | 2b | 5 |
| rTMS | 1 Hz rTMS on contralesional M1 (25mins) | RCT | Sham rTMS | 20 (10, 10) | Acceleration, Pinch Force | rTMS reduced the MEP amplitudes of contralesional M1 and the TCI duration. rTMS improved pinch acceleration of affected hand even if a plateau was reached in previous motor training. There was an improvement in motor function after rTMS which was significantly correlated with a reduced TCI duration. | Takeuchi et al., 2005 | >6 months | 1b | 7 |
| rTMS | 3 sessions of 1 Hz rTMS 3 sessions (600 pulses) | Crossover RCT | Sham rTMS | 10 | sRT, cRT, PPT, Finger tapping | Real rTMS group showed a significant decrease in SRT and cRT and improved performance in PPT with the affected hand. | Mansur et al., 2005 | <12 months | 2b | 6 |
| rTMS | 10 session of 3 Hz rTMS (10secs) during 10days | RCT | Sham rTMS | 52 (26, 26) | SSS, NIHSS, BI, RMT | Real rTMS produced a larger improvement than sham rTMS. Real rTMS induced a better SSS and BI. Patients with massive infarcts were unaffected by rTMS. | Khedr et al., 2005 | >2 weeks | 2b | 7 |
|  |  |  |  |  |  |  |  |  |  |  |
|  |  |  |  |  |  |  |  |  |  |  |
| rTMS + OT (=NEURO) | LF-rTMS + intensive OT + self-exercise (1200 1Hz pulses on UH primary motor area) 22 sessions/15 days (20min LF-rTMS + 60min OT + 60 min self-exercises /day) | RCT | CIMT  6h/d/6d wk/15d | 66 (44, 22) | FMA, WMFT, FAS | Both groups showed significant improvements on all tests but LF-rTMS + intensive OT group showed significantly larger improvements than CIMT group. | Abo et al., 2014 | ≥12 months | 1b | 6 |
| rTMS + PT | 10 sessions of 1 HzrTMS (1500 pulses) 3 days/week + PT | RCT | Sham rTMS + PT | 20 (10, 10) | mAS, FMA, FIM, SSQOL | PT + rTMS is efficient in reducing upper limb spasticity. Experimental group: decrease ≥1 in mAS score. No differences for any other outcome measures. | Barros Galvão et al., 2014 | ≥6 months | 1b | 7 |
| rTMS + PT | 1 Hz rTMS (25 mins - 2 x /day) + PT 45 mins upper limb exercise during 10 days or PT 45 mins upper limb exercise + 1 Hz rTMS (25 mins - 2 x /day) during 10 days | RCT | Sham rTMS + PT or  PT + Sham rTMS | 30 (8, 8, 14) | JTTHF, 9HPT, BBT, Grip Force | Real rTMS group: increased excitability in the 2 hemispheres and reduction of interhemispheric inhibition. Better behavioral and neurophysiologic outcomes in group receiving rTMS than PT. | Avenanti et al,. 2012 | >6 months | 1b | 8 |
| rTMS + PT | 10 sessions of 5Hz ipsilesional rTMS/750 pulses/session) during 10 days or 10 sessions of 1Hz contralesional rTMS/750pulses/session) during 10 days | RCT | Sham rTMS + PT | 60 (30, 30) | FT, AI, mRS | Both ispsilesional rTMS (5Hz) and contralesional rTMS (1Hz) led to a statistically significant improvements on FT test, AI scores and mRS. The effects were sustained 12 weeks later at follow-up. | Emara et al., 2010 | > 1 month | 1b | 9 |
| rTMS + EMG-FNMS | EMG-FNMS (20 min) + 1 Hz rTMS (15 min) during 10 consecutive working days | RCT | sham rTMS + EMG-FNMS | 24 (12, 12) | WMFT, Tardieu scale | Motor function + spasticity of affected hand improved significantly with both interventions. There were no changes of the unaffected hand. Improvement of motor function of the affected hand correlated with cortical excitability after EMG-FNMS preceded by rTMS. | Theilig et al., 2011 | 2 weeks - 58 months | 1b | 8 |
| rTMS + Motor Training | 30 mins rTMS 1Hz contralesional M1 + 45 motor training/ 3weeks | RCT | Sham rTMS | 40 (20, 20) | WMFT | No statistically significant differences between the groups in WMFT or at the level of neurological deficits. | Seniów et al., 2012 | ≤3 months | 1b | 7 |
| rTMS + Motor Practice | 22 sessions of 10 Hz rTMS 1000 pulses/20min/M1 affected hemisphere) + Motor Practice during 10 days | CT | Sham rTMS + Motor practice | 28 (14, 14) | MI, FMA, Grip Strenght, BBT | Motor function improved in both groups Group with rTMS had additional improvement in motor function. | Chang et al., 2010 | <1 month | 1b | 7 |
| rTMS + Motor Training | One session 1 Hz rTMS (25 minutes) | RCT | Sham rTMS + Motor Training | 20 (10, 10) | Acceleration, Pinching Task | rTMS increase in excitability of affected motor cortex and improvement in acceleration of the affected hand. The effect of training on pinch force. Was enhanced by rTMS. Effects lasted one week after rTMS and motor training. | Takeuchi et al., 2008 | >6 months | 1b | 7 |
| rTMS + CIMT | 20 Hz rTMS (40 stimuli, 20Hz/2sec/28sec interval) during 14 days + CIMT 50 sessions | RCT | sham rTMS + CIMT | 19 (9, 10) | WMFT, BBT | No significant effect of rTMS as an adjuvant to CIMT. The decrease in motor threshold for subjects receiving rTMS produced a change in the excitability of the motor system. | Malcolm et al., 2007 | ≥1 year | 1b | 6 |
| TBS | Intermittent TBS of AH M1 (600 stimuli) (iTBSiM1) or continuous TBS of UH M1 (600 stimuli) (cTBScM1) | Crossover CT | Sham TBS | 13 | Grip Assessment (sensory, precision), ARAT | After iTBSiM1, there were improvements in paretic grip-lift performance + facilitation of ipsilesional M1 excitability + increase ipsilesional SAI in training. Precision grip-lift improved after cTBScM1 and training. There were no effects on sensory performance. | Ackerley et al., 2014 | ≥6 months | 1b | 8 |
| rTMS + TBS | (A)10 sessions of 1 Hz rTMS of UH (10min/day) followed by 10 sessions of intermittent TBS of AH (190sec/day) during 4weeks or (B) 10 sessions of intermittent TBS of AH (190sec/day) followed by 10 sessions 1 Hz rTMS of UH (10min/day) during 4weeks | RCT (cross-over intervention) | Sham rTMS (C) | 48 (A:17, B:15, C:16) | FMA, WMFT, MRC | Group A had larger improvement than Group B on FMA, WMF, and MCR. Effects persisted for at least 3 months. | Wang et al., 2014 | 2–6 months | 1b | 9 |
| rTMS + TBS | (A)20 sessions of 1 Hz rTMS of UH M1 + intermittent TBS of AH M1 during 4 weeks or (B) 20 sessions of sham rTMS of UH + intermittent TBS during 4 weeks or (C) 20 sessions of 1Hz rTMS of UH + sham intermittent TBS) during 4 weeks | Crossover RCT | Bilateral sham control (D) | 54 (A:15, B:12, C:13, D:14) | WMFT, upper extremity FMA, Finger flexor MRC Scale, RT, FT | (A) greater muscle strength, FMA, RT, compared to (B) or (C). Correlation analyses in group A revealed a close relationship between contralesional map area decrement and WMFT, and between ipsilesional map area increment and RT decrement. | Sung et al., 2013 | 3 - 12 months | 1b | 9 |
| TBS + PT | contralesional TBS (3 stimuli 50Hz + 3 stimuli 5Hz/600 stimuli) + PT arm during 10days or ipsilesional TBS (3 stimuli 50Hz+3 stimuli 5Hz/600 stimuli) + PT arm during 10days | CT | Sham contralesional TBS + PT or  Sham ipsilesional TBS + PT | 41 (12, 12, 13, 12) | 9HPT, JTTHF, Dynamometry | No differences between groups for any outcome measure. All patients achieved small sustainable improvements. | Talelli et al., 2012 | ≥1 year | 2b | 6 |
| TBS + Motor Training | TBS on contralesional M1 or TBS on contralesional M1 | Crossover RCT | Sham TBS | 10 | ARAT | Training after real TBS improved paretic hand grip-lift kinetics. Ipsilesional M1 excitability increased after intermittent TBS of the ipsilesional M1, but decreased after continuous TBS of the contralesional M1. | Ackerley et al., 2010 | ≥6 months | 1b | 7 |
| Abo, M., W. Kakuda, R. Momosaki, H. Harashima, M. Kojima, S. Watanabe, T. Sato*, et al.* "Randomized, Multicenter, Comparative Study of Neuro Versus Cimt in Poststroke Patients with Upper Limb Hemiparesis: The Neuro-Verify Study." [In eng]. *Int J Stroke* 9, no. 5 (Jul 2014): 607-12.  Ackerley, S. J., C. M. Stinear, P. A. Barber, and W. D. Byblow. "Combining Theta Burst Stimulation with Training after Subcortical Stroke." [In eng]. *Stroke* 41, no. 7 (Jul 2010): 1568-72.  Ackerley, S. J., C. M. Stinear, P. A. Barber, and W. D. Byblow. "Priming Sensorimotor Cortex to Enhance Task-Specific Training after Subcortical Stroke." [In eng]. *Clin Neurophysiol* 125, no. 7 (Jul 2014): 1451-8.  Avenanti, A., M. Coccia, E. Ladavas, L. Provinciali, and M. G. Ceravolo. "Low-Frequency Rtms Promotes Use-Dependent Motor Plasticity in Chronic Stroke: A Randomized Trial." [In eng]. *Neurology* 78, no. 4 (Jan 24 2012): 256-64.  Barros Galvao, S. C., R. Borba Costa dos Santos, P. Borba dos Santos, M. E. Cabral, and K. Monte-Silva. "Efficacy of Coupling Repetitive Transcranial Magnetic Stimulation and Physical Therapy to Reduce Upper-Limb Spasticity in Patients with Stroke: A Randomized Controlled Trial." [In eng]. *Arch Phys Med Rehabil* 95, no. 2 (Feb 2014): 222-9.  Chang, W. H., Y. H. Kim, O. Y. Bang, S. T. Kim, Y. H. Park, and P. K. Lee. "Long-Term Effects of Rtms on Motor Recovery in Patients after Subacute Stroke." [In eng]. *J Rehabil Med* 42, no. 8 (Sep 2010): 758-64.  Chang, W. H., Y. H. Kim, W. K. Yoo, K. H. Goo, C. H. Park, S. T. Kim, and A. Pascual-Leone. "Rtms with Motor Training Modulates Cortico-Basal Ganglia-Thalamocortical Circuits in Stroke Patients." [In eng]. Restor Neurol Neurosci 30, no. 3 (2012): 179-89. DOI:[10.3233/RNN-2012-110162](http://dx.doi.org/10.3233/RNN-2012-110162)  Conforto, A. B., S. M. Anjos, G. Saposnik, E. A. Mello, E. M. Nagaya, W. Santos, Jr., K. N. Ferreiro*, et al.* "Transcranial Magnetic Stimulation in Mild to Severe Hemiparesis Early after Stroke: A Proof of Principle and Novel Approach to Improve Motor Function." [In eng]. *J Neurol* 259, no. 7 (Jul 2012): 1399-405.  Emara, T. H., R. R. Moustafa, N. M. Elnahas, A. M. Elganzoury, T. A. Abdo, S. A. Mohamed, and M. A. Eletribi. "Repetitive Transcranial Magnetic Stimulation at 1hz and 5hz Produces Sustained Improvement in Motor Function and Disability after Ischaemic Stroke." [In eng]. *Eur J Neurol* 17, no. 9 (Sep 2010): 1203-9.  Hao, Z., D. Wang, Y. Zeng, and M. Liu. "Repetitive Transcranial Magnetic Stimulation for Improving Function after Stroke." [In eng]. *Cochrane Database Syst Rev* 5 (2013): Cd008862.  Hiscock, A., S. Miller, J. Rothwell, R. C. Tallis, and V. M. Pomeroy. "Informing Dose-Finding Studies of Repetitive Transcranial Magnetic Stimulation to Enhance Motor Function: A Qualitative Systematic Review." [In eng]. *Neurorehabil Neural Repair* 22, no. 3 (May-Jun 2008): 228-49.  Hsu, W. Y., C. H. Cheng, K. K. Liao, I. H. Lee, and Y. Y. Lin. "Effects of Repetitive Transcranial Magnetic Stimulation on Motor Functions in Patients with Stroke: A Meta-Analysis." [In eng]. *Stroke* 43, no. 7 (Jul 2012): 1849-57.  Khedr, E. M., A. E. Etraby, M. Hemeda, A. M. Nasef, and A. A. Razek. "Long-Term Effect of Repetitive Transcranial Magnetic Stimulation on Motor Function Recovery after Acute Ischemic Stroke." [In eng]. *Acta Neurol Scand* 121, no. 1 (Jan 2010): 30-7.  Khedr, E. M., M. A. Ahmed, N. Fathy, and J. C. Rothwell. "Therapeutic Trial of Repetitive Transcranial Magnetic Stimulation after Acute Ischemic Stroke." [In eng]. *Neurology* 65, no. 3 (Aug 9 2005): 466-8.  Khedr, E. M., M. R. Abdel-Fadeil, A. Farghali, and M. Qaid. "Role of 1 and 3 Hz Repetitive Transcranial Magnetic Stimulation on Motor Function Recovery after Acute Ischaemic Stroke." [In eng]. *Eur J Neurol* 16, no. 12 (Dec 2009): 1323-30.  Kim, Y. H., S. H. You, M. H. Ko, J. W. Park, K. H. Lee, S. H. Jang, W. K. Yoo, and M. Hallett. "Repetitive Transcranial Magnetic Stimulation-Induced Corticomotor Excitability and Associated Motor Skill Acquisition in Chronic Stroke." [In eng]. *Stroke* 37, no. 6 (Jun 2006): 1471-6.  Le, Q., Y. Qu, Y. Tao, and S. Zhu. "Effects of Repetitive Transcranial Magnetic Stimulation on Hand Function Recovery and Excitability of the Motor Cortex after Stroke: A Meta-Analysis." [In eng]. *Am J Phys Med Rehabil* 93, no. 5 (May 2014): 422-30.  Malcolm, M. P., W. J. Triggs, K. E. Light, L. J. Gonzalez Rothi, S. Wu, K. Reid, and S. E. Nadeau. "Repetitive Transcranial Magnetic Stimulation as an Adjunct to Constraint-Induced Therapy: An Exploratory Randomized Controlled Trial." [In eng]. *Am J Phys Med Rehabil* 86, no. 9 (Sep 2007): 707-15.  Mansur, C. G., F. Fregni, P. S. Boggio, M. Riberto, J. Gallucci-Neto, C. M. Santos, T. Wagner*, et al.* "A Sham Stimulation-Controlled Trial of Rtms of the Unaffected Hemisphere in Stroke Patients." [In eng]. *Neurology* 64, no. 10 (May 24 2005): 1802-4.  Pollock, A., S. E. Farmer, M. C. Brady, P. Langhorne, G. E. Mead, J. Mehrholz, and F. van Wijck. "Interventions for Improving Upper Limb Function after Stroke." [In eng]. *Cochrane Database Syst Rev* 11 (2014): Cd010820.  Sasaki, N., S. Mizutani, W. Kakuda, and M. Abo. "Comparison of the Effects of High- and Low-Frequency Repetitive Transcranial Magnetic Stimulation on Upper Limb Hemiparesis in the Early Phase of Stroke." [In eng]. *J Stroke Cerebrovasc Dis* 22, no. 4 (May 2013): 413-8.  Seniow, J., M. Bilik, M. Lesniak, K. Waldowski, S. Iwanski, and A. Czlonkowska. "Transcranial Magnetic Stimulation Combined with Physiotherapy in Rehabilitation of Poststroke Hemiparesis: A Randomized, Double-Blind, Placebo-Controlled Study." [In eng]. *Neurorehabil Neural Repair* 26, no. 9 (Nov-Dec 2012): 1072-9.  Sung, W. H., C. P. Wang, C. L. Chou, Y. C. Chen, Y. C. Chang, and P. Y. Tsai. "Efficacy of Coupling Inhibitory and Facilitatory Repetitive Transcranial Magnetic Stimulation to Enhance Motor Recovery in Hemiplegic Stroke Patients." [In eng]. *Stroke* 44, no. 5 (May 2013): 1375-82.  Takeuchi, N., T. Chuma, Y. Matsuo, I. Watanabe, and K. Ikoma. "Repetitive Transcranial Magnetic Stimulation of Contralesional Primary Motor Cortex Improves Hand Function after Stroke." [In eng]. *Stroke* 36, no. 12 (Dec 2005): 2681-6.  Takeuchi, N., T. Tada, M. Toshima, T. Chuma, Y. Matsuo, and K. Ikoma. "Inhibition of the Unaffected Motor Cortex by 1 Hz Repetitive Transcranical Magnetic Stimulation Enhances Motor Performance and Training Effect of the Paretic Hand in Patients with Chronic Stroke." [In eng]. *J Rehabil Med* 40, no. 4 (Apr 2008): 298-303.  Takeuchi, N., T. Tada, M. Toshima, Y. Matsuo, and K. Ikoma. "Repetitive Transcranial Magnetic Stimulation over Bilateral Hemispheres Enhances Motor Function and Training Effect of Paretic Hand in Patients after Stroke." [In eng]. *J Rehabil Med* 41, no. 13 (Nov 2009): 1049-54.  Talelli, P., A. Wallace, M. Dileone, D. Hoad, B. Cheeran, R. Oliver, M. VandenBos*, et al.* "Theta Burst Stimulation in the Rehabilitation of the Upper Limb: A Semirandomized, Placebo-Controlled Trial in Chronic Stroke Patients." [In eng]. *Neurorehabil Neural Repair* 26, no. 8 (Oct 2012): 976-87.  Theilig, S., J. Podubecka, K. Bosl, R. Wiederer, and D. A. Nowak. "Functional Neuromuscular Stimulation to Improve Severe Hand Dysfunction after Stroke: Does Inhibitory Rtms Enhance Therapeutic Efficiency?" [In eng]. *Exp Neurol* 230, no. 1 (Jul 2011): 149-55.  Wang, C. P., P. Y. Tsai, T. F. Yang, K. Y. Yang, and C. C. Wang. "Differential Effect of Conditioning Sequences in Coupling Inhibitory/Facilitatory Repetitive Transcranial Magnetic Stimulation for Poststroke Motor Recovery." [In eng]. CNS Neurosci Ther 20, no. 4 (Apr 2014): 355-63. | | | | | | | | | | |
|  |  |  |  |  |  |  |  |  |  |  |

| **Table 14 : Transcranial direct current stimulation** | | |  |  |  |  |  |  |  |  |
| --- | --- | --- | --- | --- | --- | --- | --- | --- | --- | --- |
| **Intervention** | **Dosage** | **Design** | **Control** | **Number of subjects (treatment / control)** | **Outcome measures** | **Results** | **Authors** | **Time post-stroke** | **Oxford evidence level** | **Pedro Score (/10)** |
| tDCS (anodal/cathodal/dual) |  | SR | sham tDCS, no intervention or conventional therapy | 1 review (15 trials; 455 participants) | upper limb function, ADLs, upper limb impairment | Moderate-quality evidence exists of effect of tDCS on upper limb impairment [7 trials; 304 participants]. High-quality evidence indicates the absence of benefit of tDCS for ADLs outcomes compared with placebo or conventional therapy. | Pollock et al., 2014 | miscellaneous | 1a | NA |
| tDCS (anodal/cathodal/dual) |  | SR | sham tDCS and/or other intervention | 1 review (455 participants, 15 studies) | upper limb function, ADLs | Evidence of low quality is available on the effectiveness of tDCS (anodal/cathodal/dual) vs. control (sham/other intervention) for improving ADL performance and function after stroke. | Elsner et al., 2013 | miscellaneous | 1a | NA |
| tDCS (anodal M1) |  | SR Meta-Analysis | sham tDCS, no intervention or conventional therapy | 1 review (8 studies) | upper limb motor impairment (JTTHF, FMA, PS, BBT, RT, GS) | A pooled analysis showed a significant increase in scores in favor of a-tDCS. A similar effect was observed between a-tDCS and sham. | Butler et al., 2013 | miscellaneous | 1a | 7 to 9 |
| tDCS (anodal) |  | SR Meta-Analysis | sham tDCS, no intervention or conventional therapy | 1 review (14 studies) | upper limb motor impairment (JTTHF, BBT) | Results indicate that the efficacy of anodal-tDCS is dependent on current density and duration of application. A-tDCS increases corticomotor excitability in both healthy individuals and subjects with stroke. The results also show a trend in favor of motor function improvement following a-tDCS. | Bastani & Jaberzadeh 2012 | miscellaneous | 1a | 7 to 9 |
| tDCS anode - ipsilesional M1 cathode - contralesional M1 + rehabilitation treatment | 1 mA dual tDCS (20 min) + rehabilitation therapy 10 sessions during 2 weeks | RCT | Sham tDCS | 19 (10, 9) | Upper extremity FMA | FMA improved in both groups, without differences between groups. Online evaluation of tDCS was significantly higher than sham group. EEG coefficients of tDCS group were significantly higher than the sham group. | Ang et al., 2015 | ≥9 months | 2b | 5 |
| tDCS anode - ipsilesional M1 cathode - contralesional M1 + motor skill learning | 1mA dual tDCS/30 min + motor skill learning | Crossover RCT | Sham tDCS | 19 | PPT, MaxHF, ABILHAND scale, NIHSS | Dual tDCS enhanced learning with paretic limb during 1 week, compared to the sham series. | Lefebvre et al., 2015 | >6 months | 2b | 7 |
| tDCS anode - ipsilesional M1 cathode - contralesional M1 | tDCS (20min): cathodal, anodal or sham (5 days interval between sessions) | Crossover RCT | Sham tDCS | 10 | PPT, Stroop test, Pinch grip strength | Cathodal tDCS to nonlesioned M1 significantly improved affected hand dexterity (Purdue pegboard) and selective attention (Stroop), but not pinch strength. The outcomes were not improved with anodal tDCS to lesioned M1 or sham tDCS. | Au-Yeung et al., 2014 | ≥1 year | 1b | 8 |
| tDCS anode - ipsilesional M1 catode - contralateral M1 + precision grip | dual tDCS (20mins) + 10 precision grip + PTT before, during and after tDCS session | Crossover RCT | Sham tDCS | 19 | precision grip movements, PPT | Precision grip with the paretic hand improved 20mins after tDCS. The dexterity of the paretic hand improved during tDCS and culminated 20 minutes after the end of tDCS session. | Lefebvre et al., 2014 | >6 months | 1b | 8 |
| tDCS anode – ipsilesional cathode - contralesional | 2 mA tDCS (25min) : cathodal or anodal during 6 days | RCT | Sham tDCS | 40 (14, 13, 13) | NIHSS, OMCASS, BI, MRC | At 3-month follow-up, all groups had improved on all scales. Improvement was equal in the Anodal and Cathodal groups. In treated groups combined and compared with Sham, there were significant interactions for the OMCASS and BI. There was increased cortical excitability of the affected hemisphere in all groups (changes being greater in the real versus sham groups). There were borderline significant improvements in muscle strength. | Khedr et al., 2013 | acute (<1month) | 1b | 8 |
| tDCS anode – ipsilesional M1 + rehabilitation | 1 day tDCS + 1 day sham tDCS or 1 day sham tDCS +1 day tDCS | Crossover RCT | Sham tDCS + rehabilitation | 16 | Hand dexterity, manual force | Anodal and sham stimulation plus rehabilitation significantly improved manual dexterity. Significant stimulation effect existed only for anodal tDCS and not for sham tDCS. | Fusco et al., 2014 | 2 - 12 weeks | 1b | 7 |
| tDCS anode – ipsilesional + robotic therapy | tDCS (20 min) followed by robotic practice (20min) or tDCS (20 min) during robotic practice (20 min) or robotic practice (20 min) followed by tDCS (20 min) with 1 week interval between conditions | Crossover RCT | Sham tDCS + robotic therapy | 12 | movement kinematics | Movement speed increased after motor training (sham tDCS). Movement smoothness improved when tDCS was delivered before motor practice. TDCS delivered during practice did not offer any benefit, whereas it reduced speed when delivered after practice. | Giacobbe et al., 2013 | ≥6 months | 1b | 8 |
| tDCS anode – ipsilesional cathode – contralesional + arm robot | (A)30 sessions anodal tDCS (20mins) during 6weeks +AT (B)30 sessions cathodal tDCS(20mins)/6weeks +AT | RCT | (C) Sham tDCS | 96 (32, 32, 32) | FMA, UL muscle strength, BI, BBT, MRC, mAS | FMA improved in all patients at 6 weeks. No between-group differences existed with regards to initial versus finish FMA scores. No significant changes were observed between groups at 3 months. | Hesse et al., 2011 | 3 - 8 weeks | 1b | 6 |
| tDCS anode - ipsilesional M1 catode - contralateral M1 + physiotherapy/occupational therapy | 1.5 mA dual tDCS (30min) + PT/OT (60min) during 5 days | RCT | Sham tDCS | 20 (10, 10) | Upper extremity FMA, WMFT | The improvement on motor function tests was significantly greater in the real stimulation group compared to sham group. Effects outlasted the stimulation by at least 1 week. Real-stimulation group showed stronger activation of ipsilesional motor regions during paced movements of the affected limb found after intervention. There were significant activation changes in the control group. | Lindenberg et al., 2010 | >5months | 2b | 9 |
| tDCS anode - ipsilesional M1 catode - contralateral orbital region + virtual reality | 15 sessions of 2mA tDCS (13min) + 1h VRT | RCT | Sham tDCS + virtual reality | 20 (10, 10) | FMA, WMFT, mAS, grip strength, SSQOL | Both groups demonstrated gains in all evaluated areas, except for the SSQOL-UL domain. Differences between groups were only observed in wrist spasticity levels in the experimental group. | Viana et al., 2014 | <6 months | 2b | 7 |
| tDCS cathode - contralesional M1 anode - contralateral supraorbital + virtual reality | (A) 15 sessions of 2mA tDCS (30min) during 3 wks (C) Combined 15 sessions of 2mA tDCS (30min) during 3 wks + VR | RCT | (B) VR | 59 (19, 20, 20) | mAS, MMT, MFT, FMA, BBT, K-MBI | All groups improved on MMT, MFT, FMA, K-MBI scores. Improvement in MFT and FMA scores in group C was significantly higher than that of the other 2 groups. | Lee & Chun, 2014 | ≤1 month | 1b | 6 |
| tDCS anode - ipsilesional M1 catode - contralateral supraorbital region | 2 mA tDCS (20min during 5 days | RCT | Sham tDCS | 50 (25, 25) | FMA, NIHSS, BI, Rankin Scale | A significant improvement in NIHSS and FMA scores did not significantly differ when comparing tDCS and sham. | Rossi et al., 2013 | 2days | 2b | 5 |
| tDCS cathode - ipsilesional SM1 + physiotherapy | tDCS (20min/4weeks) + PT | RCT | Sham tDCS + therapy | 90 (45, 45) | mAS, FMA, BI | There were significantly more patients in the real tDCS group who improved after treatment and at 4-week follow-up. | Wu D. et al., 2013 | >2 months | 1b | 8 |
| tDCS cathode - contralesional M1 anode - contralateral supraorbital | 1mA tDCS (20mins) | Crossover RCT | Sham tDCS | 12 | acquisition & retention of complex sequential finger movement task | tDCS facilitated the acquisition of a new motor skill compared with sham & improved task retention results. Significant correlation existed between tDCS-induced improvement in training & tDCS-induced changes of intracortical inhibition. | Zimerman et al., 2012 | miscellaneous first ever stroke | 1b | 8 |
| Ang, K. K., C. Guan, K. S. Phua, C. Wang, L. Zhao, W. P. Teo, C. Chen, Y. S. Ng, and E. Chew. "Facilitating Effects of Transcranial Direct Current Stimulation on Motor Imagery Brain-Computer Interface with Robotic Feedback for Stroke Rehabilitation." [In eng]. *Arch Phys Med Rehabil* 96, no. 3 Suppl (Mar 2015): S79-87.  Au-Yeung, S. S., J. Wang, Y. Chen, and E. Chua. "Transcranial Direct Current Stimulation to Primary Motor Area Improves Hand Dexterity and Selective Attention in Chronic Stroke." [In eng]. *Am J Phys Med Rehabil* 93, no. 12 (Dec 2014): 1057-64.  Bastani, A., and S. Jaberzadeh. "Does Anodal Transcranial Direct Current Stimulation Enhance Excitability of the Motor Cortex and Motor Function in Healthy Individuals and Subjects with Stroke: A Systematic Review and Meta-Analysis." [In eng]. *Clin Neurophysiol* 123, no. 4 (Apr 2012): 644-57.  Butler, A. J., M. Shuster, E. O'Hara, K. Hurley, D. Middlebrooks, and K. Guilkey. "A Meta-Analysis of the Efficacy of Anodal Transcranial Direct Current Stimulation for Upper Limb Motor Recovery in Stroke Survivors." [In eng]. *J Hand Ther* 26, no. 2 (Apr-Jun 2013): 162-70; quiz 71.  Elsner, B., J. Kugler, M. Pohl, and J. Mehrholz. "Transcranial Direct Current Stimulation (Tdcs) for Improving Function and Activities of Daily Living in Patients after Stroke." [In eng]. *Cochrane Database Syst Rev* 11 (2013): Cd009645.  Fusco, A., M. Iosa, V. Venturiero, D. De Angelis, G. Morone, L. Maglione, M. Bragoni*, et al.* "After Vs. Priming Effects of Anodal Transcranial Direct Current Stimulation on Upper Extremity Motor Recovery in Patients with Subacute Stroke." [In eng]. *Restor Neurol Neurosci* 32, no. 2 (2014): 301-12.  Giacobbe, V., H. I. Krebs, B. T. Volpe, A. Pascual-Leone, A. Rykman, G. Zeiarati, F. Fregni*, et al.* "Transcranial Direct Current Stimulation (Tdcs) and Robotic Practice in Chronic Stroke: The Dimension of Timing." [In eng]. *NeuroRehabilitation* 33, no. 1 (2013): 49-56.  Hesse, S., A. Waldner, J. Mehrholz, C. Tomelleri, M. Pohl, and C. Werner. "Combined Transcranial Direct Current Stimulation and Robot-Assisted Arm Training in Subacute Stroke Patients: An Exploratory, Randomized Multicenter Trial." [In eng]. *Neurorehabil Neural Repair* 25, no. 9 (Nov-Dec 2011): 838-46.  Khedr, E. M., O. A. Shawky, D. H. El-Hammady, J. C. Rothwell, E. S. Darwish, O. M. Mostafa, and A. M. Tohamy. "Effect of Anodal Versus Cathodal Transcranial Direct Current Stimulation on Stroke Rehabilitation: A Pilot Randomized Controlled Trial." [In eng]. *Neurorehabil Neural Repair* 27, no. 7 (Sep 2013): 592-601.  Lee, S. J., and M. H. Chun. "Combination Transcranial Direct Current Stimulation and Virtual Reality Therapy for Upper Extremity Training in Patients with Subacute Stroke." [In eng]. *Arch Phys Med Rehabil* 95, no. 3 (Mar 2014): 431-8.  Lefebvre, S., J. L. Thonnard, P. Laloux, A. Peeters, J. Jamart, and Y. Vandermeeren. "Single Session of Dual-Tdcs Transiently Improves Precision Grip and Dexterity of the Paretic Hand after Stroke." [In eng]. *Neurorehabil Neural Repair* 28, no. 2 (Feb 2014): 100-10.  Lefebvre, S., L. Dricot, P. Laloux, W. Gradkowski, P. Desfontaines, F. Evrard, A. Peeters, J. Jamart, and Y. Vandermeeren. "Neural Substrates Underlying Stimulation-Enhanced Motor Skill Learning after Stroke." [In eng]. *Brain* 138, no. Pt 1 (Jan 2015): 149-63.  Lindenberg, R., V. Renga, L. L. Zhu, D. Nair, and G. Schlaug. "Bihemispheric Brain Stimulation Facilitates Motor Recovery in Chronic Stroke Patients." [In eng]. *Neurology* 75, no. 24 (Dec 14 2010): 2176-84.  Pollock, A., S. E. Farmer, M. C. Brady, P. Langhorne, G. E. Mead, J. Mehrholz, and F. van Wijck. "Interventions for Improving Upper Limb Function after Stroke." [In eng]. *Cochrane Database Syst Rev* 11 (2014): Cd010820.  Rossi, C., F. Sallustio, S. Di Legge, P. Stanzione, and G. Koch. "Transcranial Direct Current Stimulation of the Affected Hemisphere Does Not Accelerate Recovery of Acute Stroke Patients." [In eng]. *Eur J Neurol* 20, no. 1 (Jan 2013): 202-4.  Viana, R. T., G. E. Laurentino, R. J. Souza, J. B. Fonseca, E. M. Silva Filho, S. N. Dias, L. F. Teixeira-Salmela, and K. K. Monte-Silva. "Effects of the Addition of Transcranial Direct Current Stimulation to Virtual Reality Therapy after Stroke: A Pilot Randomized Controlled Trial." [In eng]. NeuroRehabilitation 34, no. 3 (2014): 437-46. DOI:[10.3233/NRE-141065](http://dx.doi.org/10.3233/NRE-141065)  Wu, D., L. Qian, R. D. Zorowitz, L. Zhang, Y. Qu, and Y. Yuan. "Effects on Decreasing Upper-Limb Poststroke Muscle Tone Using Transcranial Direct Current Stimulation: A Randomized Sham-Controlled Study." [In eng]. *Arch Phys Med Rehabil* 94, no. 1 (Jan 2013): 1-8.  Zimerman, M., K. F. Heise, J. Hoppe, L. G. Cohen, C. Gerloff, and F. C. Hummel. "Modulation of Training by Single-Session Transcranial Direct Current Stimulation to the Intact Motor Cortex Enhances Motor Skill Acquisition of the Paretic Hand." [In eng]. *Stroke* 43, no. 8 (Aug 2012): 2185-91. | | | | | | | | | | |

| **Table 15 : Antidepressants** | |  |  |  |  |  |  |  |  |  |
| --- | --- | --- | --- | --- | --- | --- | --- | --- | --- | --- |
| **Intervention** | **Dosage** | **Design** | **Control** | **Number of subjects (treatment / control)** | **Outcome measures** | **Results** | **Authors** | **Time post-stroke** | **Oxford evidence level** | **Pedro Score (/10)** |
| Selective serotonin reuptake inhibitors |  | SR | usual care or placebo | 52 RCTs (4060 subjects) | upper limb disability measures (BI, FIM, ADL scores,…), and motor deficits (FMA,,…) | The SMD for results in motor deficits was not significant (-0.33) [2 trials; 145 participants]. The SMD in disability scores was 0.92 [22 trials; 1310 participants] with high heterogeneity between trials. Larger effects were found if included participants were depressed at recruitment and where time post-stroke was not reported (smallest effect size if time post stroke was 3-6 mo). | Mead et al., 2012 | miscellaneous | 1a | NA |
| Antidepressant therapy in post-stroke depressed patients |  | Meta-Analysis (RCTs) | placebo (only post-stroke depressed patient) | 16 RCTs (1320 subjects) | FIM, BI, SNSS, CSS | The antidepressant group was significantly better than placebo on BI (3 RCTs) but no significant differences were found on FIM (2 RCTs). The antidepressant group improved significantly more than the placebo group on CSS (3 RCTs), but no significant differences were found on SNSS (3 RCTs). | Chen et al., 2006 | miscellaneous | 1a | NA |
|  |  |  |  |  |  |  |  |  |  |  |
| Fluoxetin or Nortriptyline + rehabilitation treatment | 10mg/day (wks1-3) 20mg/day (4-6) 30mg/day (wks 7-9) 40mg/ day (wks 10-12) or 25mg/day (wk 1) + 50mg/day (wk2) + 3.75mg/day (wks 3-6) + 100mg/ day (wks 7-12) | RCT | placebo  + rehabilitation treatment | 83 (32fl/22no/29pl) | MRS, FIM, NIHSS | MRS improved more in fluoxetine and nortriptyline groups than in placebo group. NIHSS and FIM improved in all groups (no significant time x treatment interaction). | Mikami et al., 2011 | ≤ 6 months | 1b | 6 |
| Fluoxetin + rehabilitation treatment | 20 mg/day/90 days | RCT | placebo + rehabilitation treatment | 118 (59/59) | FMA, NIHSS, MRS | The fluoxetin group showed significant larger improvements in FMA and MRS than the placebo group. | Chollet et al., 2011 | 5-10 days | 1b | 8 |
| Depressed patients: Fluoxetin or Paroxetin or Other (Amitriptyllin or Mianserin)+ rehabilitation treatment | 1x/day Fluoxetin (20mg; 10mg the first week; 40mg for 9.9% of patients) or Paroxetin (20mg; 10mg the first week) or Other (amitriptyline or mianserin) | case-control study | Non-depressed patients: rehabilitation treatment | 508 (120fl/16p/ 9other/363) | CNS, BI, RMI | The baseline BI in depressed patients was lower than in non-depressed patients. Upon discharge: depressed patients showed a significantly greater disability on BI and RMI than non-depressed patients. Both groups showed significant improvements on BI and RMI. | Paolucci et al., 2001 | at hospital admission | 2a | 6 |
| Treated depressed patients Fluoxetine/Amytriptillin or Untreated depressed patients | 20-40mg/day | CT | Untreated non- depressed patients | 64 (49 (24T/25NT) /15) | BI, CNS, RMI | Untreated non-depressed patients and treated depressed patients showed significantly larger improvements on all tests compared to untreated DP. A significant group x treatment interaction between treated and untreated DP showed larger improvements in the treated DP group for the RMI and BI. | Gainotti et al., 2001 | 1- 4 mo | 2a | 4 |
| Fluoxetin or Maprotilin + rehabilitation treatment | 1x/day/3 months Fluoxetin 20mg or Maprotiline 150mg | RCT | placebo + rehabilitation treatment | 52 (18fl/17mp/17pl) | BI, HSS (total + motor and gait subscores) | BI and HSS improved in all groups.BI and HSS were significantly better in FL group vs MP, but not significantly better vs placebo. Significantly more patients (in %) had a "good" recovery in the FL compared to the MP and placebo groups. | Dam et al., 1996 | from 1 to 6 months | 1b | 7 |
| Lithium carbonate + routine stroke treatment | 2x 300 mg/day/30 days | RCT | placebo + routine stroke treamtment | 66 (32/34) | FMA, NIHSS | FMA and NIHSS improved more with lithium carbonate than with placebo (only for patients with cortical lesions). | Mohammadianinejad et al., 2014 | 2 days | 1b | 7 |
| Reboxetine | Reboxetine 6 mg followed by placebo one week later or Placebo followed by reboxetine one week later | Crossover RCT | placebo | 22 (11/11) | ARAT, hand grip strength, maximum index finger-tapping frequency, rapid pointing movements between 2 targets (30cm/15sec) | ARAT, Grip strength and Tapping performance improved significantly with RBX and not with PBO (for the affected upper limb). Older lesions had less improvement in grip strength by RBX than younger lesions. | Wang L. E. et al., 2011 | Intervention: ≥ 4 weeks | 1b | 7 |
| Citalopram + rehabilitation treatment based on Bobath | 10 mg/day/at least 4 months | RCT | placebo + rehabilitation treatment based on Bobath | 20 (10/10) | NIHSS, BI, LS | All groups improved in NIHSS, BI and LS. NIHSS was significantly better in the citalopram vs placebo group. | Acler et al, 2009 | ≤ 10 days | 1b | 10 |
| Acler, M., E. Robol, A. Fiaschi, and P. Manganotti. "A Double Blind Placebo Rct to Investigate the Effects of Serotonergic Modulation on Brain Excitability and Motor Recovery in Stroke Patients." [In eng]. *J Neurol* 256, no. 7 (Jul 2009): 1152-8.  Chen, Y., J. J. Guo, S. Zhan, and N. C. Patel. "Treatment Effects of Antidepressants in Patients with Post-Stroke Depression: A Meta-Analysis." [In eng]. *Ann Pharmacother* 40, no. 12 (Dec 2006): 2115-22.  Chollet, F., J. Tardy, J. F. Albucher, C. Thalamas, E. Berard, C. Lamy, Y. Bejot*, et al.* "Fluoxetine for Motor Recovery after Acute Ischaemic Stroke (Flame): A Randomised Placebo-Controlled Trial." [In eng]. *Lancet Neurol* 10, no. 2 (Feb 2011): 123-30.  Dam, M., P. Tonin, A. De Boni, G. Pizzolato, S. Casson, M. Ermani, U. Freo, L. Piron, and L. Battistin. "Effects of Fluoxetine and Maprotiline on Functional Recovery in Poststroke Hemiplegic Patients Undergoing Rehabilitation Therapy." [In eng]. *Stroke* 27, no. 7 (Jul 1996): 1211-4.  Gainotti, G., G. Antonucci, C. Marra, and S. Paolucci. "Relation between Depression after Stroke, Antidepressant Therapy, and Functional Recovery." [In eng]. *J Neurol Neurosurg Psychiatry* 71, no. 2 (Aug 2001): 258-61.  Mead, G. E., C. F. Hsieh, R. Lee, M. A. Kutlubaev, A. Claxton, G. J. Hankey, and M. L. Hackett. "Selective Serotonin Reuptake Inhibitors (Ssris) for Stroke Recovery." [In eng]. *Cochrane Database Syst Rev* 11 (2012): Cd009286.  Mikami, K., R. E. Jorge, H. P. Adams, Jr., P. H. Davis, E. C. Leira, M. Jang, and R. G. Robinson. "Effect of Antidepressants on the Course of Disability Following Stroke." [In eng]. *Am J Geriatr Psychiatry* 19, no. 12 (Dec 2011): 1007-15.  Mohammadianinejad, S. E., N. Majdinasab, S. A. Sajedi, F. Abdollahi, M. M. Moqaddam, and F. Sadr. "The Effect of Lithium in Post-Stroke Motor Recovery: A Double-Blind, Placebo-Controlled, Randomized Clinical Trial." [In eng]. *Clin Neuropharmacol* 37, no. 3 (May-Jun 2014): 73-8.  Paolucci, S., G. Antonucci, M. G. Grasso, D. Morelli, E. Troisi, P. Coiro, D. De Angelis, F. Rizzi, and M. Bragoni. "Post-Stroke Depression, Antidepressant Treatment and Rehabilitation Results. A Case-Control Study." [In eng]. *Cerebrovasc Dis* 12, no. 3 (2001): 264-71.  Wang, L. E., G. R. Fink, S. Diekhoff, A. K. Rehme, S. B. Eickhoff, and C. Grefkes. "Noradrenergic Enhancement Improves Motor Network Connectivity in Stroke Patients." [In eng]. *Ann Neurol* 69, no. 2 (Feb 2011): 375-88. | | | | | | | | | | |

| **Table Table 16: Botulinum toxin** | |  |  |  |  |  |  |  |  |  |
| --- | --- | --- | --- | --- | --- | --- | --- | --- | --- | --- |
| **Intervention** | **Dosage** | **Design** | **Control** | **Number of subjects (treatment / control)** | **Outcome measures** | **Results** | **Authors** | **Time post-stroke** | **Oxford evidence level** | **Pedro Score (/10)** |
| Botulinum Toxin | AbobotulinumtoxinA | SR | Placebo | 12 RCTs | mAS, ROM, ADLs, GAS, clinical impression, caregiver burden | mAS was significantly better in most of the studies (9/12). No functional improvements were reported in most of the studies. | Dashtipour et al., 2015 | miscellaneous | 1a | NA |
| Botulinum toxin |  | SR | placebo and/or usual rehabilitation treatment | 4 reviews (11 studies; 782 participants) (54 studies; 2327 participants) (3 studies; 91 participants) (6 studies; 164 participants) | spasticity, ROM, pain | Low-quality evidence exists regarding the effectiveness of pharmacological (Botulinum toxin) therapies. | Pollock et al., 2014 | miscellaneous | 1a | NA |
| Multidisciplinary rehabilitation programs following BT injections |  | SR | BT injections + routinely available local services or lower levels of intervention or Multidisciplinary rehabilitation programs in different settings, of different types or at different intensities | 3 RCTs (92 subjects) | passive function (LASIS, DAS, AAm), active function upper extr (MAL, ARAT), pain (VAS, etc), ROM, spasticity (mAS, TS), QoL measures | There was no evidence for recommending optimal MD rehabilitation interventions after BT for post-stroke spasticity on passive function, community participation, goal achievement, caregiver burden and QoL | Demetrios et al., 2013 | miscellaneous | 1a | NA |
| Botulinum Toxin |  | SR | placebo or nonpharmacologic treatment | 10 RCTs (1000 subjects) | upper extremity function (using the DAS, ARAT, MAL, RMA, MAS), upper extremity disability (using the BI, FIM) | Higher improvements were found for passive but not for active functions in groups treated with BT compared to placebo/control groups. | Foley et al., 2013 | miscellaneous | 1a | NA |
| Botulinum Toxin | Rimabotulinum toxin B (5000U /ml) 1 injection session after a screening visit) Group high-dose: 10000 U total dose (2500 U in elbow flexors) orGroup low-dose: 15000 U total dose (5000 U in elbow flexors) | RCT | saline placebo 2mL (0.5mL in elbow flexors) or 3mL (1mL in elbow flexors) | 24 (8BT/8BT/8pl) | ROM (active elbow extension), TS, MFS, GSA, AS | Both BT groups showed significantly better effects than placebo in ROM 1 month post-injection. High-dose BT group improved more in GSA at 1 month than Low-dose BT and placebo. No improvement on MFS, AS, TS. | Gracies et al., 2014 | ≥18 mo | 1b | 8 |
| Botulinum Toxin | one injection of standard dose = exact dose calculated with estimation of muscles mass group A : half standard dose or group B: quarter standard dose | RCT | placebo | 30 (9A/10B/11) | ARAT, spasticity (using sEMG), ROM , JAMAR | No difference between groups. | Cousins et al, 2010 | ≤ 3 wk | 1b | 6 |
| Botulinum Toxin type A | 1 injection session at beginning into each of the flexor pollicis longus and adductor pollicis Group high-dose: 200U in 4ml) or Group low-dose: 120U in 2.4mL) | RCT | placebo Group High dose (4ml) or Group Low dose (2.4mL) | 109 (51BTh/21BTl/ 26plh/11pll) | mAS, DAS, CGI | BT high-dose group showed significantly better effects on mAS than placebo group. BT low-dose did not exhibit significant differences in mAS comparing with low dose placebo. DAS significantly improved in both BT and placebo groups. CGI significantly better in high dose BT group than in high dose placebo group. | Kaji et al., 2010 | ≥6 mo | 1b | 9 |
| Botulinum Toxin | BT 4 ml injection at baseline and second injection session at 12 wk Group low-dose: 90U or Group middle-dose: 180U or Group high-dose: 360U | RCT | placebo (4 mL) | 91 (21low/23mid /21high/26pl) | mAS, FIM, SF-36, pain (frequency and severity scales), functional disability | All BT groups had significantly better scores on mAS than placebo (only for the wrist flexor in Low-dose group). No differences were observed between groups on functional disability, pain assessments, FIM and SF-36. | Childers et al., 2004 | ≥6 wk | 1b | 9 |
| Botulinum Toxin type A | Botox® total dose 200 -240U (50U in 4 wrist and finger muscles + 2x20U optional thumb muscles injections) | RCT | placebo : Botulinum toxin A vehicle | 126 (64/62) | DAS, AS, GAS | Significantly more subjects had a one-point improvement in DAS in the BT group. BT group scored significantly better than placebo on AS and GAS. | Brashear et al., 2002 | ≥6 mo | 1b | 7 |
| Botulinum toxin type A + stretching or taping | OnabotulinumtoxinA maximum 600 U one injection session + Adhesive taping for 10 days | RCT | OnabotulinumtoxinA maximum 600 U one injection session + manual stretching and passive mobilization 1x/day and palmar splint for 10 days | 70 (35/35) | mAS, DAS | Adhesive taping group significantly better on mAS and DAS at one month compared to control group. | Santamato et al. 2014 | ≥6 mo | 1b | 8 |
| Botulinum Toxin + High intensity therapy (HIT) (goal directed, individualized rehabilitation program) | individualized BT injection at baseline + HI (≥3x1h/wk/±10wk) or LI (≤2x1h/wk/±10wk) | CT | Botulinum Toxin + Low intensity therapy (LIT) (goal directed, individualized rehabilitation program) | 59 (28/31) | GAS, mAS, ArmA, Global assessment scale, SRB | No significant difference in GAS but a strong trend in favor of HIT group. HIT group better on mAS than LIT group. No significant differences for ArmA, SRB, and GAS. | Demetrios et al., 2014 | ≥3 mo | 2a | 5 |
| Botulinum Toxin + standard of care | OnabotulinumtoxinA 800U maximum dose 1 injection session (+ an optional second injection session after 12-24 wk) | RCT | placebo + standard of care | 274 (139/135) | GAS, REPAS (rated according to the MAS) | Similar improvements were found for the GAS and REPAS in both groups. Patients in the BT group showed significantly larger improvements in passive goals than the placebo group. | Ward et al., 2014 | ≥3 mo | 1b | 7 |
| Botulinum Toxin + standard rehabilitation program + other antispasticity medication already in place (no dose adjustment) | Dysport® (500U) 2.5mL 1 injection session at baseline | RCT | placebo 2.5mL + standard rehabilitation program + other antispasticity medication already in place (no dose adjustment) | 163 (80/83) | mAS, FMAS, BI, MRS, ROM (active and passive) | Significantly higher improvements in mAS and passive ROM were found for the BT vs the placebo group. No differences were found for the FMA, BI, MRS and active ROM between groups. | Rosales et al., 2012 | 2 - 12 wk | 1b | 9 |
| Botulinum Toxin type A + rehabilitation therapy | BT 100U/ml 1 injection session within the 37 days after the first evaluation.Up to 300U administered according to physician's impression + 3 sessions/wk started 1 month after injections until 12-16 sessions | RCT | placebo (1mL syringes) + rehabilitation therapy | 25 (12/13) | WMFT, SIS, mAS, AROM | No significant improvement or difference was found in WMFT in both groups. BT group showed significantly better results on mAS than placebo. No significant difference was found for SIS and AROM outcomes between both groups. | Wolf et al., 2012 | 3 - 24 mo | 1b | 6 |
| Botulinum Toxin type A + multiprofessional motor rehabilitation | BT 150U/5 ml 1 injection session at beginning. 100U in deep and superficial finger flexors + 50U in flexor carpi radialis and ulnaris | RCT | multiprofessional motor rehabilitation | 18 (9/9) | mAS, REPAS, FMA, stiffness related disabilities of the fingers and the wrist | mAS and stiffness disability score showed significant higher improvements for the BT group vs control group. Significantly better scores in REPAS was found for the BT group only after 4 weeks. FMA showed no differences between groups. | Hesse et al., 2012 | 4 - 6 wk | 1b | 7 |
| Botulinum Toxin Type A + upper limb evidence based therapy (1h/day/2 days wk/4 weeks) | BT 100 U or 200 U/ml injection at 3-6-9 mo (if necessary) | RCT | upper limb evidence based therapy (1h/day/2 days wk/4 weeks) | 333 (170/163) | mAS,GS (dynamometer), MI, ARAT, pegT, BI, UL activities (Lickert scale) | BT > control (at 1 mo) on mAS. BT = control (1-3-9 mo) for GS, ARAT, pegT, BI. BT > control for UE activities (except ability to use cutlery). | Shaw et al, 2011 | < 1 mo | 1b | 6 |
| Botulinum Toxin + routine rehabilitation treatment | Dysport® 500-1000U injections on the arm/forearm at baseline and after 12 wks | RCT | placebo + routine rehabilitation treatment | 90 (52/38) | GAS (GAS attainment level + T-score), mAS, AQoL | BT group showed significant improvements in GAS and T-score at weeks 8 and 20. There was a significant group interaction on both GAS and T-score at week 20. mAS was improved in the BT group. | Turner-Stokes et al., 2010 | ≥6 mo | 1b | 9 |
| Botulinum Toxin type A) + mCIMT | Dyspor®t 200U/mL 1 injection session at baseline with 1000U injected per affected UE + mCIMT 2h/d/3d/wk + 5h/d of restraining nonaffected UE /3 mo | RCT | Botulinum Toxin type A (200U/mL) + conventional rehabilitation (neurodevelopmental techniques) 1h PT+ 1h OT/d/3d wk/3 mo | 32 (16/16) | mAS, MAL, ARAT | No difference in mAS improvements between groups (similar significant improvements). BT+mCIMT group had significant better outcome than BT+control on MAL (AOU and QOM) and on ARAT. | Sun et al., 2010 | ≥ 1 yr | 1b | 7 |
|  |  |  |  |  |  |  |  |  |  |  |
|  |  |  |  |  |  |  |  |  |  |  |
|  |  |  |  |  |  |  |  |  |  |  |
|  |  |  |  |  |  |  |  |  |  |  |
|  |  |  |  |  |  |  |  |  |  |  |
| Botulinum Toxin type A + rehabilitation and occupational therapy | BT 100U/ mL syringes. first injection session ±300-400U/subject with BT type A or placebo and second crossed-over injection session (12wk later) ±300-400U/subject BT type A or placebo + rehabilitation and OT 1h/day/2d wk/24 wk | RCcross-overT | placebo preservative-free saline solution (1mL syringes) + rehabilitation and OT | 21 (11/10) | MAL (AOU and QOM), AS (only 5 points for UE), BI, KBADLS, MOS-36, | MAL was significantly better during BT+therapy than placebo+therapy (significant difference for QOM and not for AOU. AS showed similar improvements in both groups and periods. No statistical changes were found in MOS-36, BKBADLS total score, BI (trend in favor of BT+therapy). | Meythaler et al., 2009 | ≥6 mo | 1b | 7 |
| Botulinum Toxin type A + concomitant treatment (not documented) | Dysport® first injection session 750-1000U total dose injected into principal UE spastic muscles + second injection session (12wk later) 500-1000U total dose injected into principal UE spastic muscles | RCT | placebo + concomitant treatment (not documented) | 96 (54/42) | AQoL, GAS, mAS, PDS, CBS, MMAS, VAS, PFOM | BT group significantly better on mAS, PFOM and GAS (at 20 wk) than placebo. No improvements were found on AQoL, MMAS, PDS, CBS. | McCrory et al., 2009 | ≥6 mo | 1b | 9 |
| Botulinum Toxin type A + PT sessions | Botox® first injection session 80U total dose injected in wrist and finger flexors (20U in each) + PT 1h/d/2d wk/3 mo | RCT | placebo + PT sessions | 52 (27/25) | mAS, BI, EuroQol-5D, VAS | BT group showed significantly better effect on mAS than placebo. No significant differences were found in BI, VAS and EuroQol-5D between both groups. | Jahangir et al., 2007 | ≥ 1 yr | 1b | 7 |
| Botulinum Toxin type A + intraarticular injections + physiotherapy | Botox®, Alergen® (total dose ≤ 100U) intramuscular injections (max 25U, 2 injections/muscle) + 4 mL saline intraarticular injections + PT ≥2d wk/6 wk | RCT | Saline intramuscular injections + Triamcinolone Acétonide 40 mg intraarticular injection + physiotherapy | 29 (16/13) | NRS, ROM (passive), FMA, mAS, | Both groups significantly improved in NRS, ROM and FMA (tendency in favor of BT group for NRS and ROM but no significant difference.) No improvement on mAS. | Lim et al., 2008 | ≤24 mo | 1b | 8 |
| Brashear, A., M. F. Gordon, E. Elovic, V. D. Kassicieh, C. Marciniak, M. Do, C. H. Lee, S. Jenkins, and C. Turkel. "Intramuscular Injection of Botulinum Toxin for the Treatment of Wrist and Finger Spasticity after a Stroke." [In eng]. *N Engl J Med* 347, no. 6 (Aug 8 2002): 395-400.  Childers, M. K., A. Brashear, P. Jozefczyk, M. Reding, D. Alexander, D. Good, J. M. Walcott*, et al.* "Dose-Dependent Response to Intramuscular Botulinum Toxin Type a for Upper-Limb Spasticity in Patients after a Stroke." [In eng]. *Arch Phys Med Rehabil* 85, no. 7 (Jul 2004): 1063-9.  Cousins, E., A. Ward, C. Roffe, L. Rimington, and A. Pandyan. "Does Low-Dose Botulinum Toxin Help the Recovery of Arm Function When Given Early after Stroke? A Phase Ii Randomized Controlled Pilot Study to Estimate Effect Size." [In eng]. *Clin Rehabil* 24, no. 6 (Jun 2010): 501-13.  Dashtipour, K., J. J. Chen, H. W. Walker, and M. Y. Lee. "Systematic Literature Review of Abobotulinumtoxine in Clinical Trials for Adult Upper Limb Spasticity." [In eng]. *Am J Phys Med Rehabil* 94, no. 3 (Mar 2015): 229-38.  Demetrios, M., A. Gorelik, J. Louie, C. Brand, I. J. Baguley, and F. Khan. "Outcomes of Ambulatory Rehabilitation Programmes Following Botulinum Toxin for Spasticity in Adults with Stroke." [In eng]. *J Rehabil Med* 46, no. 8 (Sep 2014): 730-7.  Demetrios, M., F. Khan, L. Turner-Stokes, C. Brand, and S. McSweeney. "Multidisciplinary Rehabilitation Following Botulinum Toxin and Other Focal Intramuscular Treatment for Post-Stroke Spasticity." [In eng]. *Cochrane Database Syst Rev* 6 (2013): Cd009689.  Foley, N., S. Pereira, K. Salter, M. M. Fernandez, M. Speechley, K. Sequeira, T. Miller, and R. Teasell. "Treatment with Botulinum Toxin Improves Upper-Extremity Function Post Stroke: A Systematic Review and Meta-Analysis." [In eng]. *Arch Phys Med Rehabil* 94, no. 5 (May 2013): 977-89.  Gracies, J. M., N. Bayle, S. Goldberg, and D. M. Simpson. "Botulinum Toxin Type B in the Spastic Arm: A Randomized, Double-Blind, Placebo-Controlled, Preliminary Study." [In eng]. *Arch Phys Med Rehabil* 95, no. 7 (Jul 2014): 1303-11.  Hesse, S., H. Mach, S. Frohlich, S. Behrend, C. Werner, and I. Melzer. "An Early Botulinum Toxin a Treatment in Subacute Stroke Patients May Prevent a Disabling Finger Flexor Stiffness Six Months Later: A Randomized Controlled Trial." [In eng]. *Clin Rehabil* 26, no. 3 (Mar 2012): 237-45.  Jahangir, A. W., H. J. Tan, M. I. Norlinah, W. Y. Nafisah, S. Ramesh, B. B. Hamidon, and A. A. Raymond. "Intramuscular Injection of Botulinum Toxin for the Treatment of Wrist and Finger Spasticity after Stroke." [In eng]. *Med J Malaysia* 62, no. 4 (Oct 2007): 319-22.  Kaji, R., Y. Osako, K. Suyama, T. Maeda, Y. Uechi, and M. Iwasaki. "Botulinum Toxin Type a in Post-Stroke Upper Limb Spasticity." [In eng]. Curr Med Res Opin 26, no. 8 (Aug 2010): 1983-92.  Lim, J. Y., J. H. Koh, and N. J. Paik. "Intramuscular Botulinum Toxin-a Reduces Hemiplegic Shoulder Pain: A Randomized, Double-Blind, Comparative Study Versus Intraarticular Triamcinolone Acetonide." [In eng]. *Stroke* 39, no. 1 (Jan 2008): 126-31.  McCrory, P., L. Turner-Stokes, I. J. Baguley, S. De Graaff, P. Katrak, J. Sandanam, L. Davies, M. Munns, and A. Hughes. "Botulinum Toxin a for Treatment of Upper Limb Spasticity Following Stroke: A Multi-Centre Randomized Placebo-Controlled Study of the Effects on Quality of Life and Other Person-Centred Outcomes." [In eng]. *J Rehabil Med* 41, no. 7 (Jun 2009): 536-44.  Meythaler, J. M., L. Vogtle, and R. C. Brunner. "A Preliminary Assessment of the Benefits of the Addition of Botulinum Toxin a to a Conventional Therapy Program on the Function of People with Longstanding Stroke." [In eng]. *Arch Phys Med Rehabil* 90, no. 9 (Sep 2009): 1453-61.  Pollock, A., S. E. Farmer, M. C. Brady, P. Langhorne, G. E. Mead, J. Mehrholz, and F. van Wijck. "Interventions for Improving Upper Limb Function after Stroke." [In eng]. *Cochrane Database Syst Rev* 11 (2014): Cd010820.  Rosales, R. L., K. H. Kong, K. J. Goh, W. Kumthornthip, V. C. Mok, M. M. Delgado-De Los Santos, K. S. Chua*, et al.* "Botulinum Toxin Injection for Hypertonicity of the Upper Extremity within 12 Weeks after Stroke: A Randomized Controlled Trial." [In eng]. *Neurorehabil Neural Repair* 26, no. 7 (Sep 2012): 812-21.  Santamato, A., M. F. Micello, F. Panza, F. Fortunato, A. Baricich, C. Cisari, A. Pilotto, et al. "Can Botulinum Toxin Type a Injection Technique Influence the Clinical Outcome of Patients with Post-Stroke Upper Limb Spasticity? A Randomized Controlled Trial Comparing Manual Needle Placement and Ultrasound-Guided Injection Techniques." [In eng]. J Neurol Sci 347, no. 1-2 (Dec 15 2014): 39-43.  Shaw, L. C., C. I. Price, F. M. van Wijck, P. Shackley, N. Steen, M. P. Barnes, G. A. Ford, L. A. Graham, and H. Rodgers. "Botulinum Toxin for the Upper Limb after Stroke (Botuls) Trial: Effect on Impairment, Activity Limitation, and Pain." [In eng]. *Stroke* 42, no. 5 (May 2011): 1371-9.  Sun, S. F., C. W. Hsu, H. P. Sun, C. W. Hwang, C. L. Yang, and J. L. Wang. "Combined Botulinum Toxin Type a with Modified Constraint-Induced Movement Therapy for Chronic Stroke Patients with Upper Extremity Spasticity: A Randomized Controlled Study." [In eng]. *Neurorehabil Neural Repair* 24, no. 1 (Jan 2010): 34-41.  Turner-Stokes, L., I. J. Baguley, S. De Graaff, P. Katrak, L. Davies, P. McCrory, and A. Hughes. "Goal Attainment Scaling in the Evaluation of Treatment of Upper Limb Spasticity with Botulinum Toxin: A Secondary Analysis from a Double-Blind Placebo-Controlled Randomized Clinical Trial." [In eng]. *J Rehabil Med* 42, no. 1 (Jan 2010): 81-9.  Ward, A. B., J. Wissel, J. Borg, P. Ertzgaard, C. Herrmann, J. Kulkarni, K. Lindgren*, et al.* "Functional Goal Achievement in Post-Stroke Spasticity Patients: The Botox(R) Economic Spasticity Trial (Best)." [In eng]. *J Rehabil Med* 46, no. 6 (Jun 2014): 504-13.  Wolf, S. L., S. B. Milton, A. Reiss, K. A. Easley, N. V. Shenvi, and P. C. Clark. "Further Assessment to Determine the Additive Effect of Botulinum Toxin Type a on an Upper Extremity Exercise Program to Enhance Function among Individuals with Chronic Stroke but Extensor Capability." [In eng]. *Arch Phys Med Rehabil* 93, no. 4 (Apr 2012): 578-87. | | | | | | | | | | |

| **Table 17: Robot-assisted therapy** | |  |  |  |  |  |  |  |  |  |
| --- | --- | --- | --- | --- | --- | --- | --- | --- | --- | --- |
| **Intervention** | **Dosage** | **Design** | **Control** | **Number of subjects (treatment / control)** | **Outcome measures** | **Results** | **Authors** | **Time post-stroke** | **Oxford evidence level** | **Pedro Score (/10)** |
| Robot-assisted training |  | SR | control treatment, placebo or no treatment | 2 reviews (19 trials; 666 participants) (12 trials; 383 participants) | upper limb impairment, ADLs | Moderate-quality evidence exists regardin the beneficial effect on upper limb impairment of RAT compared with other therapies, but no beneficial effect exists when compared with same duration therapies. Moderate-quality evidence exists regarding beneficial effect of RAT on ADLs. | Pollock et al., 2014 | miscellaneous | 1a | NA |
| Electromechanical and Robot- Assisted arm training |  | SR with RCTs and RC cross-over T (first period analyzed) in the Cochrane Stroke Group until July 2011 | other devices, other rehabilitation, placebo no treatment | 19 studies 2 cross-over design 17 parallel-group design 666 subjects | BI, FIM, FMA, MI | Electromechanical and RAT vs. No intervention: better ADL in acute phase but not in chronic phase. | Mehrholz et al., 2012 | miscellaneous | 1a | ≥5 |
| Robot-Assisted therapy: or RAT + Conventional therapy | REHAROB, MIT-MANUS, MIME, ARM-Guide, T-WREX, NeReBot | SR all RCTs until 2010 PEDro ≥5 | conventional therapy | 11 studies (+ 1 follow-up of an included RCT) 5 in CT+RAT >< CT 6 in RAT >< CT 326 subjects | FMA, FIM, MPS, MSC | Conventional therapy + RAT showed significantly better motor recovery and proximal motor control improvements than conventional therapy alone. No difference in activities of daily living. No significant difference between RAT alone and conventional therapy alone. | Norouzi-Gheidari et al., 2012 | miscellaneous | 1a | ≥5 |
| Robot-assisted training |  | SR and Meta-analysis | placebo, control and/or usual care | 1 review (10 RCTs) | upper limb function: arm function tests (ARAT, FAT,…), impairment scales (FMA, MI,…) | Results showed beneficial effects of RAT on arm function [10 trials; 295 participants] but effectiveness on hand function is still unknown [7 trials; 215 participants]. | Langhorne et al., 2009 | miscellaneous | 1a | NA |
| Electromechanical and Robotic- Assisted arm training |  | SR RCTs and cross-over studies until 2007 | Other interventions (other devices, other rehabilitation intervention or no treatment) | 11 studies (10 RCT and 1 RCcross-overT) 328 subjects | Activities of daily living (BI and FIM if available) Motor function (FMA if available) Motor strength (MI if available) | RAT did not improve activities of daily living compared to control. Motor strength and motor function are more likely to improve with RAT. | Mehrholz et al., 2008 | miscellaneous | 1a | 2 - 7 |
|  |  |  |  |  |  |  |  |  |  |  |
| Robot-assisted therapy |  | SR (RCTs until 2008) | Conventional treatment forms | 10 RCTs 218 subjects | Motor recovery (using FMA, Chedoke McMaster), ADL (using FIM) | No significant effect of RAT on ADL. Significantly better effect of RAT on motor function. | Kwakkel et al., 2008 | miscellaneous | 1a | ≥4 (4-8) |
| Assisted mvt and muscle vibration + FB with torque | AMES 30 sessions over 8-10 wks | RCT | assisted mvt and muscle vibration AMES + FB EMG | 46 (23/23) | FMA, BBT, SIS | Significant increase in all outcomes, no difference between groups. | Cordo et al., 2013 | > 1 yr severe impairment | 1b | 5 |
| Active robot-assisted/individual finger / full term intervention (FTI) | 20 sessions / 4 weeks | RCT | 2 wks early passive therapy + active robot-assisted/individual finger / half term intervention (HTI) 10 sessions/2 wks | 17 (9/8) | JTTHF, FMA, active mvmt 2nd MCP, grasp and punch power. | Both groups improved for all outcomes, larger improvement for FTI than HTI in JTTHF and wrist and hand of FMA | Hwang et al., 2012 | > 3 mo | 1b | 5 |
| Robot-assisted therapy | Pneu-Wrex 1 hour/day/+-3 days wk/ 8-9 weeks | RCT | conventional PT therapy | 27 (13/14) | FMA, MAL, BBT, RFTUE | Both groups improved on FMA and MAL. Robot-assisted therapy group improved on BBT and control on Rancho Level. | Reinkensmeyer et al., 2012 | ≥ 3 mo | 1b | 6 |
|  |  |  |  |  |  |  |  |  |  |  |
| Robot-assisting therapy | Puma 560 Robot (A) Low-dose group: 60min/day/5 days wk/3 weeks or (B) High-dose group: 120min/day/5 days wk/3 weeks | RCT | conventional therapy 60min/day/5 days wk/3 weeks | 54 (l19/h17/ct18) | FMA, MRCMP granding scale FIM, mAS, WMFT | FMA increased significantly more in high-dose RAT than in control group at posttreatment. At 6 months, high group RAT showed significantly higher improvements in mAS than low-dose RAT. | Burgar et al., 2011 | 7 - 21 d | 1b | 6 |
| Robot-assisted therapy | InMotion2.0 Shoulder/Arm Robot (2 degrees of freedom) or InMotion Linear Sshoulder/Elbow Robot (3 degrees of freedom): 60min/day/3days wk/ 6 weeks | RCT | intensive conventional arm exorcise (ICAE) | 62 (20plan/21plan-v/ 21ICAE) | FMA, WMFT, SIS | No difference between groups on FMA and WMFT. SIS better in 3 degrees of freedom-RAT than in ICAE. | Conroy et al., 2011 | ≥6 mo (if ischemic)  ≥12 mo (if hemorrhagic) | 1b | 5 |
| Robotic-therapy with assistance but without object manipulation (reaching) |  | CT | Robotic-therapy with assistance and actual object manipulation Robotic-therapy with assistance and virtual object as a goal | 47 (32reach/10act/5virt) | FMA | No difference of total score between groups. Shoulder and elbow subcomponents improved "more in group ""reaching" while wrist and hand" improved more in groups with manipulation. | Krebs et al., 2008 | 1 - 5 yr | 2a | 4 |
| Robot-assisted therapy | MIME system 50min/day (24 sessions in 2 months) + 5min tone normalization before and after sessions | RCT | conventional treatment neurodevelopmental therapy | 30 (13/14) | FMA, BI, FIM strength performed with MVCs | RAT better on FMA after 2 months but no difference at 6 months follow-up. No difference after 2 months in FIM but RAT better at 6 months follow-up. | Lum et al., 2002 | ≥ 6 mo | 1b | 7 |
| Robot-assisted therapy + 3h/day PT | Amadeo Robotic System 40min/day/4-5 days wk/4-5 wk (20 sessions) | RCT | Occupational Therapy (OT) 40min/day/5days wk/4 wks (20 sessions) + 3h/day PT | 20 (11/9) | FMA, BBT, mAS, MI, MRC, BI | Both improved on FMA, BBT, MI, MRC. Only RAT improved on mAS. | Sale et al., 2014a | 30 d (±7 d) | 1b | 6 |
| Robot-assisted task-oriented therapy (RAT) + usual care | Haptic Master 2x30min/day/4 days wk/8 weeks | RCT | Task oriented non-robotic therapy +usual care | 22 (11/11) | FMA, (ARAT), MAL, EuroQuol-5D, SF-36 | No between-group differences in FMA, ARAT and MAL No improvement on FMA. ARAT improved in robot-assisted treatment group and MAL in both groups but better in control group. | Timmermans et al., 2014 | ≥12 mo | 1b | 8 |
|  |  |  |  |  |  |  |  |  |  |  |
| Robotic-assisted therapy + 3h/day PT | MIT-MANUS/InMotion2 45min/day /5days wk / 6 wks | RCT | Control (conventional) therapy + 3h/day PT | 53 (26/27) | FMA, mAS, passive ROM, MI | FMA increased in both groups. | Sale et al., 2014b | 30 d (±7 d) | 1b | 8 |
| Robot-assisted therapy (RAT) shoulder-elbow + antigravity + wrist + grasp-hand units 4 blocks of 3 weeks (increasing number units used) or intensive therapy structured protocol using conventional rehabilitation techniques | 60min/day/3days wk/12 weeks | RCT | usual care | 127 (49RAT/50int/28ctrl) | FMA, WMFT, SIS | Non-significant trend towards RAT better than usual care but less than intensive therapy on FMA. | Lo et al., 2010 | ≥ 6 mo | 1b | 6 |
| Robot-assisted therapy+ conventional therapy | MIT-Manus + conventional therapy: 4-5h/week | RCT | conventional therapy | 20 (10/10) | FIM, FMA, motor status scale (MSS) | No difference between conventional and conventional + RAT for FIM and FMA. MSS showed better in conventional + RAT in portions on the limb directly involved in the RAT. | Aisen et al., 1997 | 3 wk (±1 wk) | 1b | 7 |
| Aisen, M. L., H. I. Krebs, N. Hogan, F. McDowell, and B. T. Volpe. "The Effect of Robot-Assisted Therapy and Rehabilitative Training on Motor Recovery Following Stroke." [In eng]. *Arch Neurol* 54, no. 4 (Apr 1997): 443-6.  Burgar, C. G., P. S. Lum, A. M. Scremin, S. L. Garber, H. F. Van der Loos, D. Kenney, and P. Shor. "Robot-Assisted Upper-Limb Therapy in Acute Rehabilitation Setting Following Stroke: Department of Veterans Affairs Multisite Clinical Trial." [In eng]. *J Rehabil Res Dev* 48, no. 4 (2011): 445-58.  Conroy, S. S., J. Whitall, L. Dipietro, L. M. Jones-Lush, M. Zhan, M. A. Finley, G. F. Wittenberg, H. I. Krebs, and C. T. Bever. "Effect of Gravity on Robot-Assisted Motor Training after Chronic Stroke: A Randomized Trial." [In eng]. *Arch Phys Med Rehabil* 92, no. 11 (Nov 2011): 1754-61.  Cordo, P., S. Wolf, J. S. Lou, R. Bogey, M. Stevenson, J. Hayes, and E. Roth. "Treatment of Severe Hand Impairment Following Stroke by Combining Assisted Movement, Muscle Vibration, and Biofeedback." [In eng]. *J Neurol Phys Ther* 37, no. 4 (Dec 2013): 194-203.  Hwang, C. H., J. W. Seong, and D. S. Son. "Individual Finger Synchronized Robot-Assisted Hand Rehabilitation in Subacute to Chronic Stroke: A Prospective Randomized Clinical Trial of Efficacy." [In eng]. *Clin Rehabil* 26, no. 8 (Aug 2012): 696-704.  Krebs, H. I., S. Mernoff, S. E. Fasoli, R. Hughes, J. Stein, and N. Hogan. "A Comparison of Functional and Impairment-Based Robotic Training in Severe to Moderate Chronic Stroke: A Pilot Study." [In eng]. *NeuroRehabilitation* 23, no. 1 (2008): 81-7.  Langhorne, P., F. Coupar, and A. Pollock. "Motor Recovery after Stroke: A Systematic Review." [In eng]. *Lancet Neurol* 8, no. 8 (Aug 2009): 741-54.  Lo, A. C., P. D. Guarino, L. G. Richards, J. K. Haselkorn, G. F. Wittenberg, D. G. Federman, R. J. Ringer*, et al.* "Robot-Assisted Therapy for Long-Term Upper-Limb Impairment after Stroke." [In eng]. *N Engl J Med* 362, no. 19 (May 13 2010): 1772-83.  Mehrholz, J., A. Hadrich, T. Platz, J. Kugler, and M. Pohl. "Electromechanical and Robot-Assisted Arm Training for Improving Generic Activities of Daily Living, Arm Function, and Arm Muscle Strength after Stroke." [In eng]. *Cochrane Database Syst Rev* 6 (2012): Cd006876.  Mehrholz, J., T. Platz, J. Kugler, and M. Pohl. "Electromechanical and Robot-Assisted Arm Training for Improving Arm Function and Activities of Daily Living after Stroke." [In eng]. *Cochrane Database Syst Rev* 4 (2008): Cd006876.  Norouzi-Gheidari, N., P. S. Archambault, and J. Fung. "Effects of Robot-Assisted Therapy on Stroke Rehabilitation in Upper Limbs: Systematic Review and Meta-Analysis of the Literature." [In eng]. J Rehabil Res Dev 49, no. 4 (2012): 479-96.  Pollock, A., S. E. Farmer, M. C. Brady, P. Langhorne, G. E. Mead, J. Mehrholz, and F. van Wijck. "Interventions for Improving Upper Limb Function after Stroke." [In eng]. *Cochrane Database Syst Rev* 11 (2014): Cd010820.  Reinkensmeyer, D. J., E. T. Wolbrecht, V. Chan, C. Chou, S. C. Cramer, and J. E. Bobrow. "Comparison of Three-Dimensional, Assist-as-Needed Robotic Arm/Hand Movement Training Provided with Pneu-Wrex to Conventional Tabletop Therapy after Chronic Stroke." [In eng]. *Am J Phys Med Rehabil* 91, no. 11 Suppl 3 (Nov 2012): S232-41.  Kwakkel, G., B. J. Kollen, and H. I. Krebs. "Effects of Robot-Assisted Therapy on Upper Limb Recovery after Stroke: A Systematic Review." [In eng]. *Neurorehabil Neural Repair* 22, no. 2 (Mar-Apr 2008): 111-21.  Lum, P. S., C. G. Burgar, P. C. Shor, M. Majmundar, and M. Van der Loos. "Robot-Assisted Movement Training Compared with Conventional Therapy Techniques for the Rehabilitation of Upper-Limb Motor Function after Stroke." [In eng]. *Arch Phys Med Rehabil* 83, no. 7 (Jul 2002): 952-9.  Sale, P., M. Franceschini, S. Mazzoleni, E. Palma, M. Agosti, and F. Posteraro. « Effects of Upper Limb Robot-Assisted Therapy on Motor Recovery in Subacute Stroke Patients.” *J Neuroeng Rehabil* 11 (Jun 2014b):104  Sale, P., S. Mazzoleni, V. Lombardi, D. Galafate, M. P. Massimiani, F. Posteraro, C. Damiani, and M. Franceschini. “Recovery of Hand Function with Robot-Assisted Therapy in Acute Stroke Patients: a Randomized-controlled Trial”. *Int J Rehabil Res* 37, no.3 (Sep 2014a): 236-42.  Timmermans, A. A., R. J. Lemmens, M. Monfrance, R. P. Geers, W. Bakx, R. J. Smeets, and H. A. Seelen. "Effects of Task-Oriented Robot Training on Arm Function, Activity, and Quality of Life in Chronic Stroke Patients: A Randomized Controlled Trial." [In eng]. *J Neuroeng Rehabil* 11 (2014): 45. | | | | | | | | | | |
|  |  |  |  |  |  |  |  |  |  |  |

| **Table 18 : Virtual reality** | | | | |  |  |  |  |  |  |
| --- | --- | --- | --- | --- | --- | --- | --- | --- | --- | --- |
| **Intervention** | **Dosage** | **Design** | **Control** | **Number of subjects (treatment / control)** | **Outcomemeasures** | **Results** | **Authors** | **Time post-stroke** | **Oxford evidence level** | **Pedro Score (/10)** |
| virtual reality |  | SR | control treatment/intervention | 1 review (19 trials; 565 participants) | upper limb function, ADLs, upper limb impairment, grip strength | Moderate-quality evidence shows a beneficial effect of virtual reality intervention on upper limb function and impairment [7 trials; 205 participants]. No benefit was found on grip strength [2 trials; 44 participants]. | Pollock et al., 2014 | miscellaneous | 1a | NA |
| Virtual reality & Interactive video gaming |  | SR | control treatment/intervention | 1 SR (19 trials, 565 participants) | usual care, control intervention | Study sample sizes were small and outcome measures varied, limiting the ability in which studies could be compared. Primary outcomes: results were statistically significant for arm function No statistically significant effects were observed for grip strength or gait speed. It was impossible to determine the effect on global motor function due to insufficient numbers of comparable studies. Few adverse events were reported across studies. | Laver et al., 2011 | miscellaneous | 1a | NA |
| Virtual reality |  | SR | control treatment/intervention | 1 Meta-Analysis (12 trials; 195 participants) | upper limb function, ADLs, upper limb impairment, grip strength | 11 of 12 studies showed a significant benefit towards VR on selected outcomes. No significant differences were observed for BBT or motor function. 14. There was a 7% improvement in motor impairment and 20.1% improvement in motor function after VR. | Saposnik & Levin, 2011 | miscellaneous | 1a | NA |
| Virtual reality |  | SR | control treatment/intervention | SR (6 studies) | usual care, control intervention | Level 1b evidence suggesting an advantage to training in immersive VR versus no therapy in UE rehabilitation, and level 5 evidence for training in immersive VR versus conventional therapy. There is level 4 evidence showing conflicting results for training in non-immersive VR versus no therapy, and level 2b evidence for training in non-immersive VR versus conventional therapy. | Henderson et al., 2007 | miscellaneous | 1a | NA |
|  |  |  |  |  |  |  |  |  |  |  |
| Virtual Reality Training Using Xbox Kinect | Virtual reality training using Xbox Kinect  1h/day (3xweek)/6weeks | RCT | conventional occupational therapy  1h/day (3xweek)/6weeks | 40 (20, 20) | FMA, BBT, AROM | After intervention, there were significant improvements from baseline values in range of motion of the upper extremity, FMA and BBT. At follow-up, significant differences were observed between the groups in range of motion, FMA and BBT. | Sin & Lee, 2013 | >6 months | 1b | 7 |
| Virtual reality | Virtual reality  9 sessions/ 3weeks | RCT | Conventional therapy  9 sessions/ 3weeks | 18 | upper limb Motricity Index, ARAT | Both groups demonstrated small, but non-significant, changes of their UE impairment and activity levels. Compliance was high; only 2 people reported side-effects from VR. | Crosbie et al., 2012 | 6–12 months | 1b | 8 |
| Virtual Reality | Virtual reality with  (A) Vision-based tracking  (B) Combined visual and haptic feedback  35mins (5xweek)/4weeks | RCT | (C) Passive exoskeleton 35mins (5xweek)/4weeks | 44 (17, 15, 16) | BI, Motricity Index, CAHAI, FM, mAS, BBT | Significant within-subject improvements existed on most of standard clinical evaluation scales for all groups. The beneficial effects of VR-based training are modulated by the use/nonuse of compensatory movement strategies and the specific sensorimotor contingencies presented to the user, that is, visual feedback versus combined visual haptic feedback. | Cameirão et al., 2012 | >1 year | 1b | 6 |
| Virtual Reality (Nintendo Wii gaming) | Virtual reality Nintendo Wii gaming  8 sessions (60mins)/2weeks | RCT | Recreational therapy  8 sessions (60mins)/2weeks | 22 (11, 11) | WMFT, BBT, SIS | Relative to the recreational therapy group, participants in the VR-Wii arm had a significant improvement in mean motor function of 7 seconds, after adjustment for age, baseline functional status and stroke severity. | Saposnik et al., 2010 | <6 months | 1b | 5 |
| Virtual reality | Virtual reality 1h/day (5xweek)/4weeks | RCT | Physical therapy 1h/day (5xweek)/4weeks | 36 (18, 18) | FMA-UE, ABILHAND, Ashworth scales | Both interventions significantly improved outcome scores after treatment, but only the FMA-UE showed significant differences in the comparison between groups. | Piron et al., 2009 | 7–32 months | 1b | 7 |
| Reinforced feedback in virtual environment | Reinforced feedback in virtual environment 2hours (5xweek)/4weeks | RCT | Conventional therapy  2hours (5xweek)/4weeks | 44 (23, 21) | FMA-UE, FIM, kinematics parameters | The FMA-UE, FIM, time and peak were significantly higher in RFVE group after treatment, but not speed. The patients with hemorrhagic stroke significantly improved on FIM, time and peak, whereas patients with ischemic stroke improved significantly only on speed in RFVE treatment group. | Kiper et al., 2014 | >1year | 1b | 5 |
| Virtual Reality Environment | 3D virtual environment  12 sessions/ 4weeks | RCT | Physical environment  12 sessions/ 4weeks | 32 (13, 12) | Kinematics, clinical arm motor impairment, activity level, arm use | Overall performance and activity increased in both groups. Only participants in VE group improved shoulder horizontal adduction and flexion. After VE training, the mildly affected group increased elbow extension. The moderately-to-severely affected group in VE increased arm use and reaching ability. The moderately-to-severely affected group in PE increased reaching ability earlier and both elbow extension and arm use, but these changes were accompanied by increased compensatory trunk displacement. | Subramanian et al., 2013 | 6-60 months | 1b | 6 |
| Virtual reality + Physical therapy | VR + PT 30mins (5xweek)/4weeks | RCT | Physical therapy 30mins (5xweek)/4weeks | 24 (12, 12) | FMA, BBT, ROM, mAS, Grip Strength | Both groups significantly improved the upper limb function, excepting spasticity, after intervention. A significant group–time interaction was shown only for shoulder/ elbow/wrist items of FMA, BBT, grip strength, and ROM of wrist flexion, extension, and ulnar deviation. | Lee et al., 2014 | miscellaneous av 9 months | 1b | 5 |
| Virtual reality + conventional therapy | Virtual reality + conventional therapy 2hours (5xweek)/4weeks | RCT | Conventional therapy 2hours (5xweek)/4weeks | 376 (263, 113) | FIM, FM-UE | Both treatments significantly improved FM- UE and FIM scores, but the improvement obtained with VR was significantly greater than the one achieved with conventional therapy alone. | Turolla et al., 2013 | miscellaneous | 1b | 6 |
| VR + tDCS | (B) VR 15 sessions 30min 5xweek)/3weeks (C) Simultaneously VR 15 sessions + 2mA tDCS 30min(5xweek)/3weeks | RCT | (A) 15 sessions of 2mA tDCS 30min(5xweek)/3weeks | 59 (19, 20, 20) | mAS, MMT, MFT, FMS, BBT, K-MBI | All groups improved in MMT, MFT, FMS, K-MBI scores. Improvements in MFT and FMS in group C were significantly higher than in the other 2 groups. | Lee & Chun, 2014 | ≤1 month | 1b | 6 |
| Cameirao, M. S., S. B. Badia, E. Duarte, A. Frisoli, and P. F. Verschure. "The Combined Impact of Virtual Reality Neurorehabilitation and Its Interfaces on Upper Extremity Functional Recovery in Patients with Chronic Stroke." [In eng]. *Stroke* 43, no. 10 (Oct 2012): 2720-8.  Crosbie, J. H., S. Lennon, M. C. McGoldrick, M. D. McNeill, and S. M. McDonough. "Virtual Reality in the Rehabilitation of the Arm after Hemiplegic Stroke: A Randomized Controlled Pilot Study." [In eng]. *Clin Rehabil* 26, no. 9 (Sep 2012): 798-806.  Henderson, A., N. Korner-Bitensky, and M. Levin. "Virtual Reality in Stroke Rehabilitation: A Systematic Review of Its Effectiveness for Upper Limb Motor Recovery." [In eng]. *Top Stroke Rehabil* 14, no. 2 (Mar-Apr 2007): 52-61.  Kiper, P., and M. Agostini. "Reinforced Feedback in Virtual Environment for Rehabilitation of Upper Extremity Dysfunction after Stroke: Preliminary Data from a Randomized Controlled Trial." Biomed Res Int (2014): 752128.  Laver, K. E., S. George, S. Thomas, J. E. Deutsch, and M. Crotty. "Virtual Reality for Stroke Rehabilitation." [In eng]. *Cochrane Database Syst Rev* 9 (2011): Cd008349.  Lee, D., M. Lee, K. Lee, and C. Song. "Asymmetric Training Using Virtual Reality Reflection Equipment and the Enhancement of Upper Limb Function in Stroke Patients: A Randomized Controlled Trial." [In eng]. *J Stroke Cerebrovasc Dis* 23, no. 6 (Jul 2014): 1319-26.  Lee, S. J., and M. H. Chun. "Combination Transcranial Direct Current Stimulation and Virtual Reality Therapy for Upper Extremity Training in Patients with Subacute Stroke." [In eng]. *Arch Phys Med Rehabil* 95, no. 3 (Mar 2014): 431-8.  Piron, L., A. Turolla, M. Agostini, C. Zucconi, F. Cortese, M. Zampolini, M. Zannini*, et al.* "Exercises for Paretic Upper Limb after Stroke: A Combined Virtual-Reality and Telemedicine Approach." [In eng]. *J Rehabil Med* 41, no. 12 (Nov 2009): 1016-102.  Pollock, A., S. E. Farmer, M. C. Brady, P. Langhorne, G. E. Mead, J. Mehrholz, and F. van Wijck. "Interventions for Improving Upper Limb Function after Stroke." [In eng]. *Cochrane Database Syst Rev* 11 (2014): Cd010820.  Saposnik, G., and M. Levin. "Virtual Reality in Stroke Rehabilitation: A Meta-Analysis and Implications for Clinicians." [In eng]. Stroke 42, no. 5 (May 2011): 1380-6.  Saposnik, G., R. Teasell, M. Mamdani, J. Hall, W. McIlroy, D. Cheung, K. E. Thorpe, L. G. Cohen, and M. Bayley. "Effectiveness of Virtual Reality Using Wii Gaming Technology in Stroke Rehabilitation: A Pilot Randomized Clinical Trial and Proof of Principle." [In eng]. *Stroke* 41, no. 7 (Jul 2010): 1477-84.  Sin, H., and G. Lee. "Additional Virtual Reality Training Using Xbox Kinect in Stroke Survivors with Hemiplegia." [In eng]. *Am J Phys Med Rehabil* 92, no. 10 (Oct 2013): 871-80.  Subramanian, S. K., C. B. Lourenco, G. Chilingaryan, H. Sveistrup, and M. F. Levin. "Arm Motor Recovery Using a Virtual Reality Intervention in Chronic Stroke: Randomized Control Trial." [In eng]. *Neurorehabil Neural Repair* 27, no. 1 (Jan 2013): 13-23.  Turolla, A., M. Dam, L. Ventura, P. Tonin, M. Agostini, C. Zucconi, P. Kiper, A. Cagnin, and L. Piron. "Virtual Reality for the Rehabilitation of the Upper Limb Motor Function after Stroke: A Prospective Controlled Trial." [In eng]. *J Neuroeng Rehabil* 10 (2013): 85. | | | | | | | | | | |

| **Table 19 : Music therapy** | | | |  |  |  |  |  |  |  | |
| --- | --- | --- | --- | --- | --- | --- | --- | --- | --- | --- | --- |
| **Intervention** | **Design** | **Dosage** | **Control** | **Number of subjects (treatment / control)** | **Outcome measures** | **Results** | **Authors** | **Time post-stroke** | **Oxford evidence level** | **Pedro Score (/10)** |  |
| Music therapy |  | SR |  | 1 review (2 trials; 41 participants) | upper limb recovery | There is a lack of trial evidence to permit any conclusion related to the effectiveness of music therapy. | Pollock et al., 2014 | miscellaneous | 1a | NA |  |
| Improvisation/electronic device | 2/week for 10 weeks RAS (1 session) | (SR including 2 studies on UE) | physical exercise with non-cued repetitive training | 20 (10/10), 21 (self-ctrl) | Shoulder ROM | No significant differences between groups. | Bradt et al.,, 2010 | Chronic: 93,4 (+-49,5) days Acute: 16,1 (+-4) days | 1a | N/A |  |
| MusicGlove | (A) MusicGlove (B) Isotrainer 6 sessions of 1 hour 3 days/2 week per condition | Cross-over RCT | conventional hand therapy same dosage | 12 | BBT, 9HPT, one month follow-up | Larger improvements in music glove group than control group for all outcomes, persisting at follow-up. | Friedman et al.,2014 | > 6 months | 1c | 5 |  |
| Music-supported therapy | 15 sessions/30 min/3 weeks in addition to conventional | RCT | conventional intervention same dosage | 62 (32/30) | ARAT, BBT, 9HPT | Better outcomes in MST vs conventional therapy alone on speed, smoothness and precision of movements | Altenmüller et al. , 2009 | 1,9 month (+-1,3) | 1c | 4 |  |
| Altenmuller, E., J. Marco-Pallares, T. F. Munte, and S. Schneider. "Neural Reorganization Underlies Improvement in Stroke-Induced Motor Dysfunction by Music-Supported Therapy." [In eng]. *Ann N Y Acad Sci* 1169 (Jul 2009): 395-405.  Bradt, J., W. L. Magee, C. Dileo, B. L. Wheeler, and E. McGilloway. "Music Therapy for Acquired Brain Injury." [In eng]. *Cochrane Database Syst Rev* 7 (2010): Cd006787.  Friedman, N., V. Chan, A. N. Reinkensmeyer, A. Beroukhim, G. J. Zambrano, M. Bachman, and D. J. Reinkensmeyer. "Retraining and Assessing Hand Movement after Stroke Using the Musicglove: Comparison with Conventional Hand Therapy and Isometric Grip Training." [In eng]. *J Neuroeng Rehabil* 11 (2014): 76.  Pollock, A., S. E. Farmer, M. C. Brady, P. Langhorne, G. E. Mead, J. Mehrholz, and F. van Wijck. "Interventions for Improving Upper Limb Function after Stroke." [In eng]. *Cochrane Database Syst Rev* 11 (2014): Cd010820. | | | | | | | | | | |  |
