# Supplementary material for: Rehabilitation of Motor Function after Stroke: A Multiple Systematic Review Focused on Techniques to Stimulate Upper Extremity Recovery
Source: Front Hum Neurosci. 2016 Sep 13;10:442. doi: 10.3389/fnhum.2016.00442 (PMC5020059; doi:10.3389/fnhum.2016.00442)
Supplement: Supplementary file 3 [file Table2.docx]

**List of abbreviations (in the tables)**

10HPT= 10 Hole Peg Test

16HPT= 16-Hole Peg Test

2-PDT= 2-Point Discrimination Test

9HPT = 9-Hole Peg Test

Aam= Arm Activity Measure

AAUT= Actual Amount of Use Test

ADL = Activities of Daily Living

AETT= Augmented Exercise Therapy Time

AH = Affected Hemisphere

AI = Activity Index

AOU= Amount of Use

AQoL= Assessment of Quality of Life

AR = Arm Robot

ARAT = Action Research Arm Test

ArmA= Arm Activity measure

AROM= Active Range Of Motion

AS = Ashworth Scale

AT= Antidepressant Therapy

AutoCITE= Automated Constraint-Induced Therapy Extension

BAT = Bilateral Arm Training

BATRAC = Bilateral Arm Training with Rhythmic Auditory Cueing

BBS= Berg Balance Scale

BBT = Box and Blocks Test

BFM = Brunnström-Fugl-Meyer

BI = Barthel Index

BIT = Bilateral Isokinematic Training

BoNTA = Botulinum Neurotoxin Type A

BS= Brunnstrom stages of motor recovery

BT= Botulinum Toxin

CAHAI = Chedoke Arm and Hand Activity Inventory

CBS= Carer Burden Scale

CERB = Custom-Designed Computerized Evaluation and Re-Education Biofeedback

CGI= Clinical Global Impression

CIMT = Constraint-Induced Movement Therapy

CMII= Chedoke McMaster Impairment Inventory

CNS= Canadian Neurological Scale

CR= Conventional Rehabilitation

cRT = Four-Choice Reaction Time

CSS= Chinese Stroke Scale

CT = Control Treatment

d = day(s)

DAS= Disability Assessment Scale

dCIT = Distributed Constraint-Induced Therapy

dCIT-TR= Distributed Constraint-Induced Therapy combined with Trunk Restraint

DMCT = Dose-Matched Conventional Treatment

DMTE = Dose-Matched Therapeutic Exercises

EADLS= Extended Activities of Daily Living Scale

EMF= Emory Function Test

EMG-FNMS = Electromyography-Triggered Functional Neuromuscular Stimulation

EmNSA= Erasmus Modification of the Nottingham Sensory Assessment

EQ-5D= European Quality of Life-5 Dimensions

extADLS= Extended Activities of Daily Living Scale

FAI= Frenchay Activities Index

FAS = Functional Abillity Score

FAT= Frenchay Arm Test

FES = Functional Electric Stimulation

FIM= Functional Independence Measure

FIMM= Functional Independance Measure Motor items

FMA Sh/El = Fugl-Meyer Scale for Shoulder/Elbow

FMA UE = Fugl–Meyer Upper Extremity

FMA W/H = Fugl-Meyer Scale for Wrist/Hand

FMA = Fugl Meyer Assessment

FT = Finger Tapping Task

FTT= Force Tracking Test

FU= Forced Use Therapy

GAS= Goal Attainment Scale

GAscale= Global Assessment Scale

GPT= Grooved Pegboard Test

GS = Grip Strengh

GSA= Global Self-Assessment

GWMFT= Graded Wolf Motor Function Test

h= hour(s)

HF= High Frequency

HI-CIMT= High-Intensity Constraint-Induced Movement Therapy

IADL = Instrumental Activities of Daily Living

ICR= Intensive Conventional Rehabilitation

iTBS = Intermittent Theta Burst Stimulation

JAMAR= JAMAR dynamometer

JMTT= Joint Movement Tracking Test

JTTHF = Jebsen-Taylor Test of Hand Function

K-MBI = Korean-Modified Barthel Index

KBADLS= Klein-Bell Activities of Daily Living Scale

KFET= Kocaeli Functional Evaluation Test

LASIS= Leeds Arm Spasticity Impact Scale

LBSMT = Learning-Based Sensorimotor Training

LF-rTMS= Low-Frequency Repetitive Transcranial Magnetic Stimulation

LF= Low Frequency

LI-CIMT= Low-Intensity Constraint-Induced Movement Therapy

LS= Lindmark Scale

M1 = Primary Motor Cortex

MA = Movement Accuracy

MAL = Motor Activity Log

MAL-28= Motor Activity Log-28

mAS = Modified Ashworth Scale

MAS = Motor Assessment Scale

MaxHF = Maximal Hand Force

mBAT= Modified Bilateral Arm Training

mBATRAC = Modified Bilateral Arm Training with Rhythmic Auditory Cueing

mBI= Modified Barthel Index

mCIMT = Modified Constraint-Induced Movement Therapy

MCP= Metacarpophalangeal

MD= Multidisciplinary

MEP = Motor-Evoked Potential

MESASP= Motor Evaluation Scale for Arm in Stroke Patients

MFS= Modified Frenchay Scale

MFT = Manual Function Test

MG= Mesh-Glove Afferent Stimulation

MI = Motricity Index

min= minute(s)

MMAS= Modified Motor Assessment Scale

MMDT= Minnesota Manual Dexterity Test

MMT = Manual Muscle Test

mo= month(s)

MoAS= Motor Assessment Scale

MORO= Modified Opposition Restriction Orthosis

MOS-36= MOS-36 Item Short-Form Health Status Survey

MPE= Motor Power Examination

MPS= Motor Power Scale

MRC = Medical Research Council scale, measuring muscle power

MRP= Motor Relearning Programme

mRS = Modified Rankin Scale

MSL= Motor Skill Learning

MSS = Motor Status Scale

mT = Movement Time

MT= Mirror Therapy

MTST= Meaningful Task Specific Training

MVCs= Maximum Voluntary Contractions

NEADL= Nottingham Extended Activities of Daily Living

NEURO = NovEl Intervention Using Repetitive TMS and Intensive Occupational Therapy

NHP= Nottingham Health Profile

NIHSS= National Institute of Health Stroke Scale

NMES = Neuromuscular Electrical Stimulation

NRS= Numeric Rating Scale

NSA= Nottingham Sensory Assessment

OMCASS = Orgogozo’s MCA Scale

OT = Occupational Therapy

PDS= Patient Disability Scale

PE = Physical Environment

PNF= Proprioceptive Neuromuscular Facilitation

PNS = Peripheral Nerve Stimulation

PPT= Purdue Pegboard Test

PS = Pinch Strength

PT = Physical Therapy/Physiotherapy

QoL= Quality of life

QOM= Quality Of Movement

RAI= Ritchie Articular Index

RAP= Rehabilitation Activities Profile

RAT= Robot Assissted Therapy

RBAT = Robot-Assisted Bilateral Arm Training

REPAS= Resistance to passive movements

RFI= "Repty" Functional Index

RFTUE= Rancho Functional Test for the hemiplegic/paretic Upper Extremity

RFVE = Reinforced Feedback in Virtual Environment

RGS = Rehabilitation Gaming System

RMA= Rivermead Motor Assessment

RMI= Rivermead mobility index

RMT = Resting Motor Threshold

RNSA= Revised Nottingham Sensory Assessment

ROM = Range of Motion

RPSS = Repetitive Peripheral Nerve Sensory Stimulation

RS= Rankin Scale

RT = Reaction Time

RTP= Repetitive Task Practice

S(N)SS= Scandinavian (Neurological) Stroke Scale

SAI = Short Latency Afferent Inhibition

sEMG= Surface Electromyography

SF-36= Medical Outcomes Study 36-Item Short-Form Health Survey

SHFT= Sollerman Hand Function Test

SIAS= Stroke Impairment Assessment Set

SIS = Stroke Impact Scale

SIS-16 = Stroke Impact Scale-16

SM1 = Primary Sensorimotor Cortex

SMES= Sodring Motor Evaluation Scale

SRB= Self-Rated Burden

sRT = Simple Reaction Time

SSQOL = Stroke Specific Quality of Life Scale

SSS = Scandinavian Stroke Scale

ST= Sollerman's Test

sT= Speech Therapy

TCI = Transcallosal Inhibition

TENS = Transcutaneous Electrical Nerve Stimulator

TMS= Transcranial Magnetic Stimulation

TOT = Task-Oriented Training

TOT_ST = Task-Oriented Therapy with Personalized Resistance

TR = Traditional Rehabilitation

TRT = Task-Related Training

TS= Tardieu Scale

UE = Upper Extremity

UH = Unaffected Hemisphere

UL= Upper Limb(s)

URT = Unilateral Robot-Assisted Arm Training

VAS= Visual Analog Scale

VE = Virtual Environments

VMC = Voluntary Muscle Contraction

VR = Virtual Reality

VRS= Visual Ranking Score

wk= week(s)

WMFT= Wolf Motor Function Test

yr= year(s)
